# Intramolecular Hydrogen Bond Driven Conformational Selectivity of Coumarin Derivatives of Resorcin[4]arene

**DOI:** 10.3390/ijms21176160

**Published:** 2020-08-26

**Authors:** Anna Szafraniec, Waldemar Iwanek

**Affiliations:** 1 Faculty of Chemistry, Adam Mickiewicz University, Uniwersytetu Poznańskiego 8, 60-614 Poznań, Poland; szafraniec.anna0203@gmail.com; 2 Faculty of Chemical Technology and Engineering, UTP, University of Science and Technology, Seminaryjna 3, 85-326 Bydgoszcz, Poland

**Keywords:** resorcin[4]arene, conformations, hydrogen bond, DFT calculations, energy

## Abstract

In this study, the synthesis and structure of 4-aminocoumarin derivatives of resorcin[4]arene were investigated. Spectroscopic analysis and quantum mechanical calculations showed that this molecule undertakes a crown-*in* conformation in chloroform. The conformations of the aminocoumarin derivative of resorcin[4]arene were compared with a hydroxycoumarin derivative of resorcin[4]arene, and the effect of the substituent on the conformational selectivity of the coumarin derivatives of resorcin[4]arene was demonstrated. Both UV-VIS and fluorescence spectroscopy for the coumarin derivative of resorcin[4]arene (**3**) were performed, and a strong fluorescence quenching of derivative **3** compared to 4-aminocoumarin was observed.

## 1. Introduction

New methods for designing and synthesizing supramolecular systems that can be applied to highly functional materials are desirable and applied to macrocyclic compounds, such as cyclodextrins [1], crown ethers [2], pillararenes [3], cucurbitulirs [4], and calixarenes [5]. Calixarenes, which are rigid vase-like molecules, are widespread throughout supramolecular chemistry. Their large-scale synthesis is relatively simple and requires cheap starting materials. They can be selectively functionalised at different positions which render them attractive starting materials from a synthetic standpoint.

Classified as calixarenes, resorcin[4]arenes are particularly noteworthy due to their presence in numerous reactive centres [6,7]. Resorcin[4]arenes can be functionalised, especially at their upper rim, to provide selective structural, physical, and chemical properties [8]. Functionalised resorcin[4]arenes have been used in supramolecular host–guest (H–G) chemistry [9], nanoparticle synthesis [10], optical [11], chemosensors [12], and for separation applications [13]. Larger supramolecular systems with attractive molecular architecture, such as cavitands [14], carcaplexes [15], dimers [16], and hexamers, have also been constructed [17].

The reaction most frequently used to modify the structure of the crown conformation in resorcin[4]arenes is the Mannich reaction [18]. Recently, we showed that a Michael reaction can also be used to modify the structure of resorcin[4]arenes [19]. The product achieved is a derivative where the main skeleton of the resorcin[4]arene is connected to the introduced substituents using a methylene bridge. Such a resorcin[4]arene derivative structure is possible as, depending on the type of the substituent, the substituents can be placed outside (*out*) or above (*in*) the cavity of the resorcin[4]arene skeleton [20].

Hydrogen bonds are fundamental intermolecular interactions for controlling the organisation of biological structures in living organisms. In this context, they control the synthesis of spatially developed systems, containing biologically important heterocyclic units in their conformationally rigid or labile structure, that can interact with important biological structures at the molecular level. By selecting appropriate proton-donor-acceptor substitutes and the type of solvent and temperature, one can use hydrogen bonds to determine the selectivity and rigidity of a molecular form of a specific type of resorcin[4]arene conformer. Such a possibility is interesting for modelling applications and for creating enzyme mimics using calixarenes [21].

**Scheme 1:**
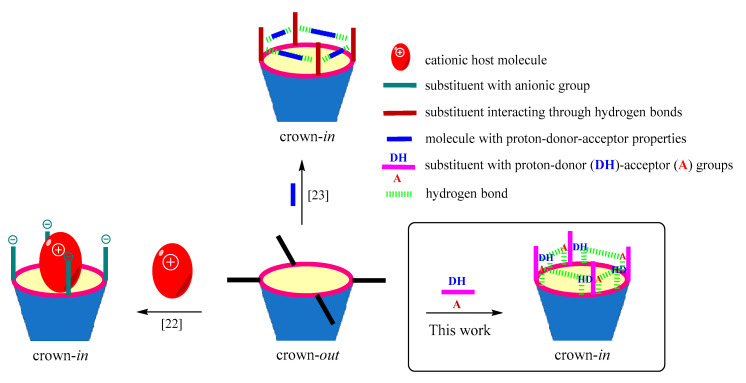
A general scheme showing the synthesis of crown-*in* conformation in resorcin[4]arenes.

Scheme 1 shows the possibilities for controlling the crown-*in*-*out* conformations depending on the type of substituent and guest or helper particle introduced. The introduction of a substituent with ionic groups into the molecule favours the complexation of the cationic guest molecule causing a simultaneous conformation change [22]. Functionalization of resorcin[4]arenes with substituents able to interact through hydrogen bonds, using a helper molecule which exhibits proton-donor-acceptor ability, forces and stiffens the crown-*in* resorcin[4]arene conformation [23].

**Scheme 2:**
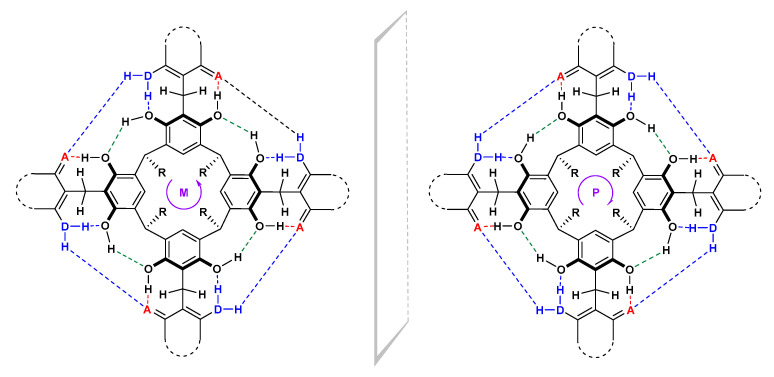
A general scheme showing crown-*in* chiral enantiomers (M, P) of resorcin[4]arene derivatives.

The concept of selective formation of crown-*in* conformers of resorcin[4]arene derivatives, which is based on the introduction of substituents into the upper rim which have proton-donor(**DH**)-acceptor(**A**) properties, is new, and there are no references to the literature (Scheme 1 and Scheme 2). This opens up new possibilities for the synthesis of resorcin[4]arene derivatives with interesting structural, chirality, and spectroscopic properties.

The presented work shows the possibilities of selective control in crown-*in*-*out* conformers of resorcin[4]arenes by substituting in coumarin derivatives attached to the resorcin[4]arene platform. The conformational variability of their structure, along with their fluorescence properties, may be of interest in enzyme mimics.

The crown-*in* conformational architecture of the coumarin derivative of resorcin[4]arene presented below is similar (not identical) to that of cavitands. It is formed through a system of intramolecular hydrogen bonds of coumarin units attached to the methylene bridge, and not through the covalent modification of hydroxyl groups as seen in cavitands. The conformational change of cavitands is quite well known and can be triggered by different stimuli, such as solvent nature [24], temperature [25], pH [26], electric charge [27], or light [28].

## 2. Results and Discussion

**Scheme 3:**
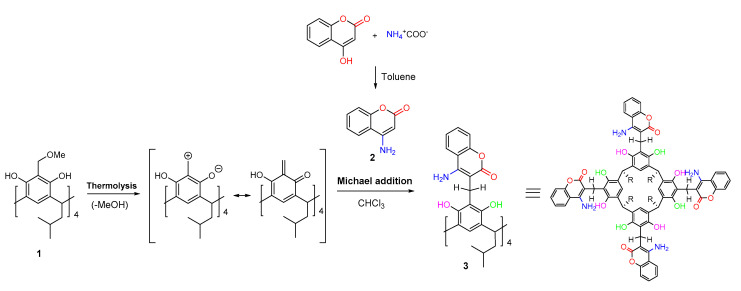
A general scheme for the synthesis of the aminocoumarin derivative of resorcin[4]arene **3**.

For the synthesis of coumarin derivatives of resorcin[4]arene, we used an *o*-quinomethide intermediate of resorcin[4]arene in the Michael 1,4 addition reaction. Scheme 3 shows the cascade reaction by a thermolysis/1,4-Michael addition of the methoxy derivative of resorcin[4]arene (**1**) with 4-aminocoumarin (**2**) in CHCl_3_. The 4-aminocoumarin was synthesised by heating 4-hydroxycoumarin with ammonium acetate in toluene for 3 h with an azeotropic cap. The obtained product was crystallised from ethyl acetate. Then, 100 mg of compound **1** and 73 mg (4eq) of 4-aminocoumarin (**2**) were weighed, dissolved in 5 mL of chloroform, and placed in a reaction vessel. The syntheses were performed using a Monowave 50 Reactor, conducting the reaction at 160 °C for 15 min. This reaction led to the product coumarin[4]arene (**3**) with a yield of 89%, after washing several times with a series of solvents to give a spectrally pure product. The resulting product had low solubility in organic solvents (e.g., insoluble in DMSO), which significantly limited the possibilities for testing its receptor properties. The following conformational analysis of compound **3** is based on the 1D- and 2D-NMR spectra of a saturated solution of compound **3** in CDCl_3_.

**Figure 1:**
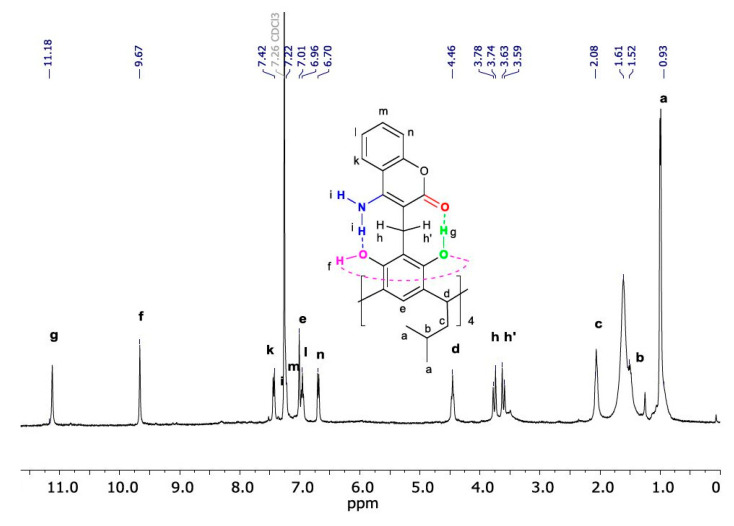
The ^1^H NMR spectrum (400 MHz) of derivative **3** in CDCl_3_ at 298 K.

The ^1^H nuclear magnetic resonance (NMR) spectrum of the 4-aminocoumarin derivative of resorcin[4]arene (**3**) in chloroform shows the spin-spin coupling of the diastereotopic protons in the methylene group -CH_2_(**h**,**h’**) (Figure 1). This suggests that the structure of the molecule in this solvent becomes rigid, forming strong intramolecular hydrogen bonds. Diastereotopic protons of the methylene group were assigned using a ^1^H-^13^C HMBC experiment (Figure S1). Due to the lower intensity of *cis*-^3^*J* signals compared to the *trans*-^3^*J* coupling [29], it was concluded that proton (**h**) is closer to the amine NH_2_ group of coumarin unit, whilst proton (**h’**) is closer to the carbonyl group.

**Figure 2:**
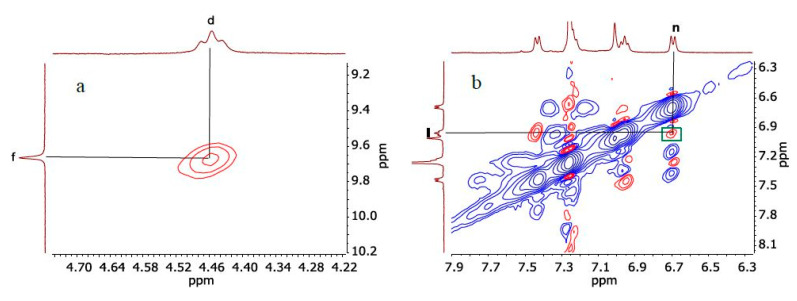
(**a**) A section of the ROESY spectrum (400 MHz, CDCl_3_, 298 K) of derivative **3,** with a signal coming from interactions of the methine proton CH(**d**) with the hydroxyl group OH(**f**) proton; (**b**) An expansion of the ROESY spectrum (400 MHz, CDCl_3_, 298 K) for the aromatic proton range of compound **3**—the green resonance coming from proton interaction (**n** and **l**).

Protons forming intramolecular hydrogen bonds (**g**, **f**, **i**) have a high ppm shift *δ*_iso_ of 7.2 to 11.2 ppm. A proton at a chemical shift *δ*_iso_ of 11.18 ppm was assigned to the proton of the OH(**g**) hydroxyl group that forms hydrogen bonds with the carbonyl group in the coumarin part of resorcin[4]arene. A δ_iso_ of 9.67 ppm was assigned to the proton of the hydroxyl group OH(**f**) in the resorcin[4]arene, which formed hydrogen bonds with the hydroxyl group OH(**g**) of the neighbouring resorcin unit in the resorcin[4]arene. Moreover, using a ROESY experiment, the proton in the hydroxyl group OH(**f**) of the resorcin[4]arene was shown to be located in the direct vicinity of the proton of the methine group (**d**) in the lower rim of the resorcin[4]arene (Figure 2a). Analysis of the 1D and 2D NMR spectra indicated that the proton signals of the NH_2_(**i**) amine group in derivative **3** were obscured by the chloroform proton signal. The crown-*in* conformation of compound **3** was also confirmed by the ROESY spectrum, where the coumarin proton (**n**) of one coumarin unit was close in space to the coumarin proton (**l**) of the adjacent coumarin unit, which further supports a crown-*in* conformation (Figure 2b). For the crown-*out* conformation of derivative **3**, the interaction of these protons was not possible due to their large through-space distance. Moreover, for the hydroxycoumarin derivative of resorcin[4]arene, such an interaction was not observed.

**Figure 3:**
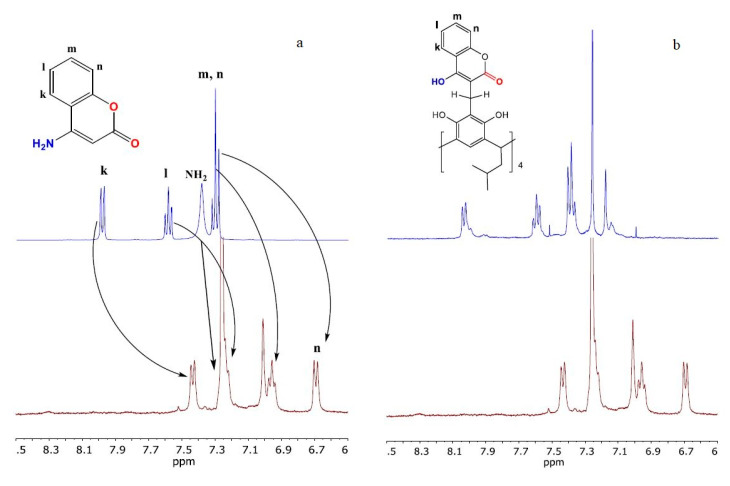
(**a**) A comparison of fragments of the ^1^H NMR (400 MHz) 4-aminocoumarin spectra (top, blue) and derivative **3** (bottom, brown) in CDCl_3_ at 298 K.; (**b**) A comparison of fragments of the ^1^H NMR (400 MHz) hydroxycoumarin derivative of resorcin[4]arene spectra (top, blue) and derivative **3** (bottom, brown) in CDCl_3_ at 298 K.

All of the aromatic proton signals of the coumarin units in derivative **3** shifted towards a lower ppm than 4-aminocoumarin by ~0.6 ppm (Figure 3a). The greatest shift towards a lower ppm was observed for the coumarin proton C-H(n) in the resorcin[4]arene derivative **3**. Furthermore, a significant shift in the aromatic proton signals of the hydroxycoumarin derivative of the resorcin[4]arene, which we have shown to be a crown-*out* conformation [30], was observed in the coumarin proton towards a lower ppm in Figure 3b. This supports the formation of the crown-*in* conformation of the aminocoumarin derivative of the resorcin[4]arene.

**Figure 4:**
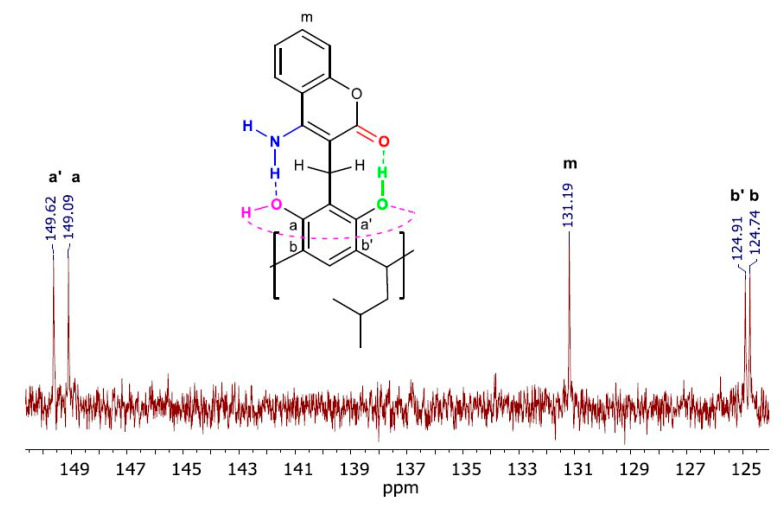
An expansion of the ^13^C NMR spectrum (400 MHz, CDCl_3_, 298 K) of derivative **3**, displaying the chemical shifts of aromatic carbons (a,a’ and b,b’) in the rings of the resorcinol unit.

The aromatic carbon atoms of the resorcin[4]arene’s skeleton were sensitive to the formation of the cyclochiral structure in the resorcin[4]arene. This was particularly evident in the aromatic carbons associated with the hydroxyl groups which participate in the hydrogen bond formation. This affected the electron density around the carbon atoms and caused alterations in their chemical shift. The observed difference in the chemical shifts of aromatic carbons (a,a’) in the rings of the resorcinol unit in compound **3** was 0.53 ppm. This change is comparable with the observed changes in the chemical shifts of carbon atoms (0.6–0.7 ppm) connected by oxygen atoms in cyclochiral tetramethoxy derivatives of resorcin[4]arenes [31,32]. These atoms are not only a sensitive indicator of the formation of cyclochiral derivatives of resorcin[4]arenes, but also of the specific resorcin[4]arene conformation selectivity. Figure 4 shows a section of the ^13^C NMR spectrum of derivative **3**, presenting the chemical shifts of the aromatic carbons (a,a’ and b,b’) in the resorcinol units rings.

Geometry optimisation calculations (Table 1) on the crown-*in* and crown-*out* derivative **3** in the gaseous phase, CHCl_3_, and DMSO, were performed using the density functional theory (DFT) approach within the Gaussian 09 program suite [33] and DFTB/GNF2-xTB method [34]. The geometry was optimised using the B3LYP functional, employing a 6-311G(d,p) basis set. The solvent effects were considered within the SCRF theory using the polarised continuum model (PCM) approach to model the interaction with the solvent. The fast semi-empirical DFTB/GFN2-xTB method was also applied using the S. Grimme software [35]. The solvent’s effects were considered within the generalised Born (GB) solvent accessible surface area (SASA) termed GBSA.

**Figure 5:**
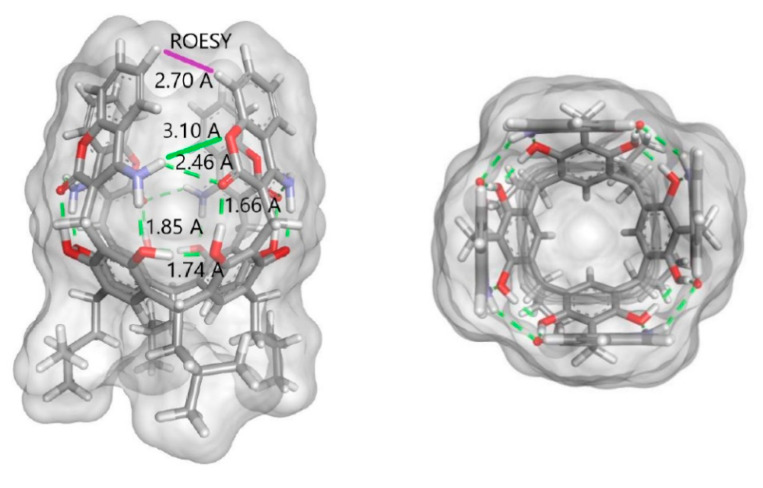
The structure of the aminocoumarin derivative of the resorcin[4]arene calculated by the DFT/B3LYP method in CHCl_3_. The side projection is given on the **left**, with the top projection on the **right**.

As previously mentioned, the solubility of derivative **3** in organic solvents is very low, especially in DMSO. This fact rendered the acquirement of the ^1^H NMR spectrum in this solvent not possible. To explain the reason for such low solubility of derivative **3** in DMSO, calculations showing how the strong proton-acceptor properties of DMSO can lead to a transformation of the crown-*in* conformation into a crown-*out* conformation of derivative **3** were undertaken. All the calculations indicated that the most energy-stable conformer in all states (gaseous phase, CHCl_3_, and DMSO) is the crown-*in* conformation. The calculated differences in the energy of the formations of both conformers in CHCl_3_ are, respectively: 28.34 kJ/mol (DFTB/GFN2) and 19.54 kJ/mol (DFT/B3LYP). The Boltzmann distribution of the conformers at 298 K shows that more than 99.9% of the conformer population in chloroform is the crown-*in* conformer. Moreover, in the case of both methods used for theoretical calculations, we observed lower energy of the crown-*in* conformation in chloroform than in DMSO. The difference is, respectively: 20.33 kJ/mol for the DFTB/GFN2 method and 9.41 kJ/mol for the DFT/B3LYP method. This is related to the difference in polarity and the different proton-donor-acceptor properties of both solvents. Figure 5 shows the structure of derivative **3** calculated by the DFT/B3LYP method in CDCl_3_, with the lengths of intramolecular hydrogen bonds and distance of the protons (**l**,**n** ROESY) in the adjacent coumarin units marked.

**Figure 6:**
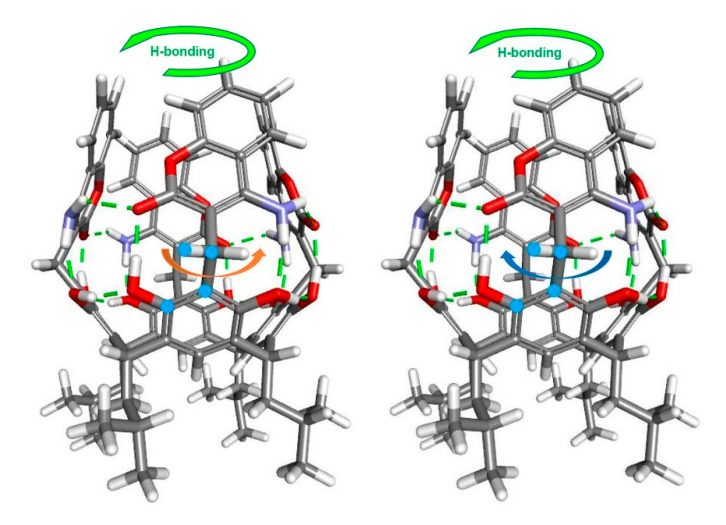
The structure of derivative **3** with the hydrogen bonding system direction (green arrow) and the direction of the marked dihedral angle: **left**—orange arrow, **right**—blue arrow.

To estimate the activation energy of the coumarin unit rotation in the crown-*in* derivative **3**, the total energy changes were scanned modifying the dihedral angle with a 3° deg step for the carbon atoms marked blue (Figure 6). Scanning of the total energy changes through a full range of dihedral angle rotation was performed in CHCl_3_ using a GFN2-xTB semiempirical DFTB method. Geometry optimization was performed for each change in the dihedral angle. The calculations were completed using an Econv/Eh of 1 × 10^−7^, a Gconv/Eh·α^−1^ of 2 × 10^−4^, and an accuracy of 0.05.

**Figure 7:**
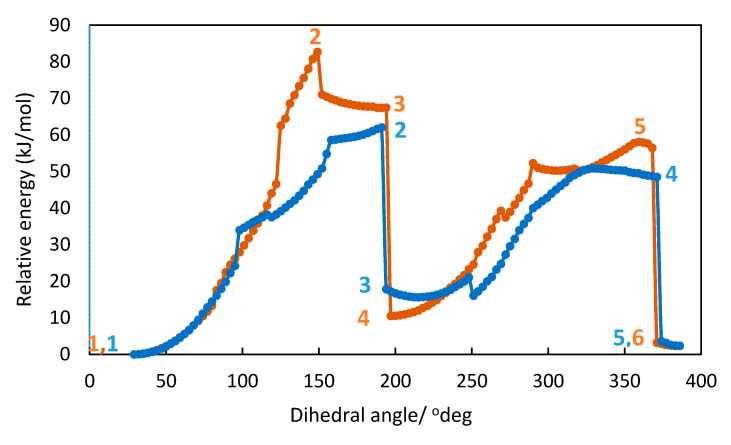
The changes in the relative energy of derivative **3** with respect to the dihedral angle (blue curve—angle changes from the left, orange curve—from the right). The calculations were performed using the GFN2-xTB semiempirical DFT in CHCl_3_.

While maintaining the directionality of the intramolecular hydrogen bond in derivative **3** (as shown in Figure 6), the total energy of the system relating to the rotation of one coumarin unit strongly depended on the directional changes of the dihedral angle, outlined in Figure 7.

**Figure 8:**
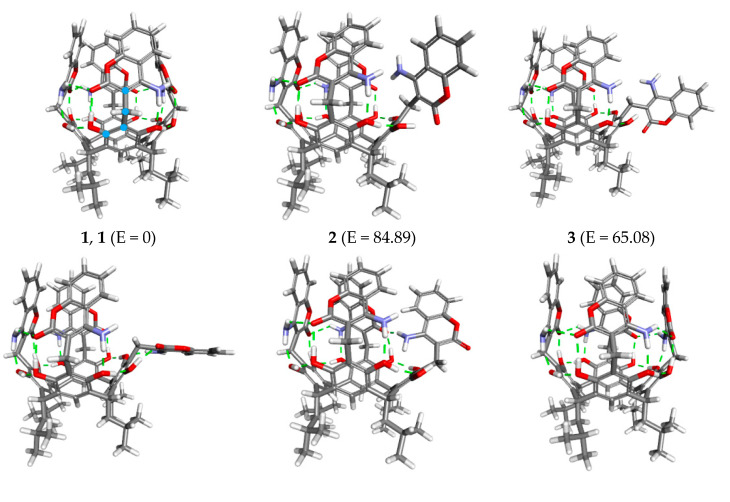

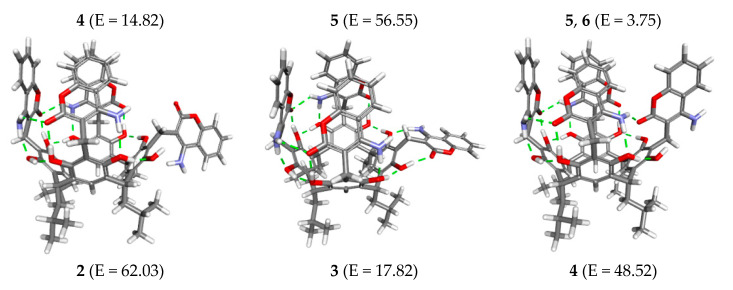
The structures of compound **3** with relative energies (kJ/mol) corresponding to the labelled points in Figure 7.

The change in the total energy of derivative **3**, along with the rotation of the dihedral angle from the left (blue curve), proceeds through lower energy values than the rotation in the opposite direction (orange curve). The difference in total energy between the point with the maximum energy and the starting point on the orange curve is the activation energy for the change of position of the coumarin substituent and is ∆E_2−1_ = 62.03 kJ/mol. When the coumarin unit is turned in the opposite direction, the activation energy is greater (∆E_2−1_ = 84.89 kJ/mol). On both curves, there are characteristic “jumps” in the total energy when rotating the dihedral angle. These distinct changes are associated with the recovery of the hydrogen bond by rotating the coumarin unit. In Figure 7, the characteristic points which represent distinct changes in the total energy of derivative **3** with respect to the dihedral angle are labelled with numbers. The structures at these colour-coded labelled energy values are shown in Figure 8.

To evaluate the susceptibility of compound **3** as the host on the complexation of small molecules, the synthesis of compound **3** was carried out in tetrahydrofuran (THF) under the same reaction conditions as in CHCl_3_. The ^1^H NMR spectrum in CDCl_3_ of the product **3** synthesised in THF no show of THF molecule located in the cavity. There are visible chemical shifts of the THF molecule protons (1.85 ppm and 3.75 ppm respectively), which are consistent with the chemical shifts of the free THF molecules in chloroform (1.85 ppm and 3.76 ppm) [36]. The reason for this may be the insufficiently accessible, top-limited, and small volume of the cavity of compound **3**. In Figure S2, the ^1^H NMR spectra of compound **3** in the presence and absence of THF molecule are compared. The spectra have the same chemical shifts of hydrogen atoms and resolution. Only the proton signal of the hydroxyl group OH(**g**) of compound **3** slightly expanded. It is probably related to its interaction with THF molecules located outside the cavity which are visible in the ^1^H NMR spectrum.

**Figure 9:**
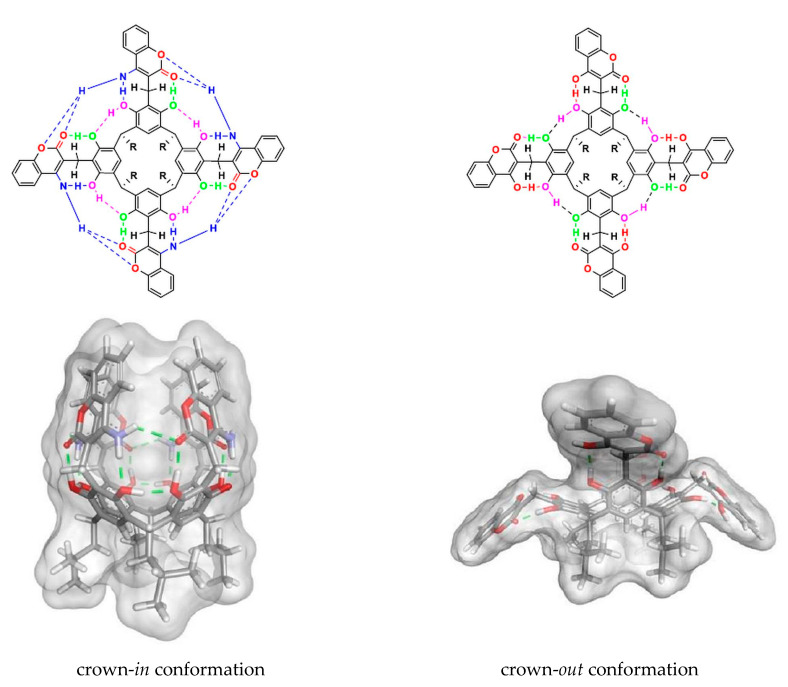
The crown-*in* conformation of the aminocoumarin derivative of resorcin[4]arene (**left**) and crown-*out* conformation of the hydroxycoumarin derivative of resorcin[4]arene (**right**) in CHCl_3_.

The effect of the substituent (-NH_2_ or -OH group), at position 4 in the coumarin molecule, on the conformation of the coumarin derivatives of resorcin[4]arenes is shown in Figure 9. The possible formation of additional intramolecular hydrogen bonds by the amine group vs. the hydroxyl group in the coumarin derivatives of resorcin[4]arenes led to selective formation of the crown-*in* conformer as it is the more stable form.

**Figure 10:**
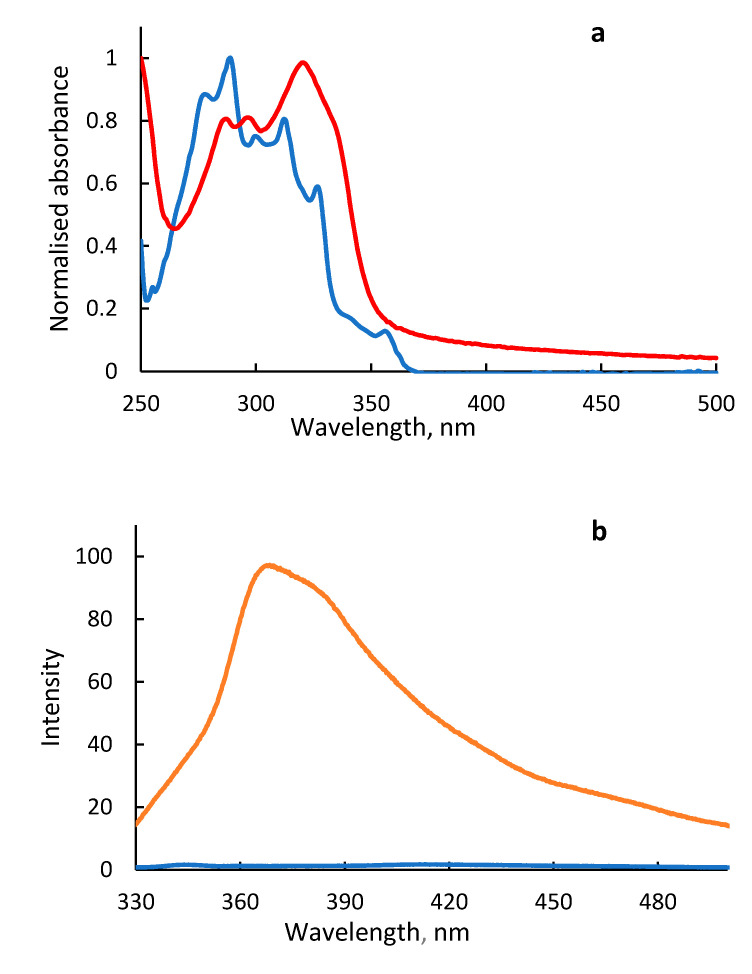
(**a**) The normalised absorption spectra of 4-aminocoumarin (c = 1.76 × 10^−5^ M, blue) and derivative **3** (c = 1.84 × 10^−6^ M, red) in CHCl_3_; (**b**) with a comparison of the intensity of the fluorescence spectra of 4-aminocoumarin (orange line) and derivative **3** (blue line) in CHCl_3_ (λ_exc_ = 310 nm, A = 0.2).

Due to their fluorescent properties, coumarin and its derivatives are often used as fluorescent markers in biological research [37] and as fluorescent chemosensors in cavitands [38]. Therefore, the inclusion of coumarin units in the resorcin[4]arene platform should directly affect the fluorescence of coumarin[4]arene, compared to pure 4-aminocoumarin. To compare the changes of the aminocoumarin spectroscopic properties versus the aminocoumarin derivative of the resorcin[4]arene, comparative measurements of the absorption and fluorescence spectra were performed in chloroform. From the UV-VIS spectra, we observed a bathochromic shift of the electron bands of derivative **3** compared to 4-aminocoumarin. Furthermore, the oscillatory structure of the band is less distinct. Moreover, in the range from 350 to 500 nm, a small absorption was observed, which is probably related to the intramolecular hydrogen bond system balancing the crown-*in* conformation of derivative **3**, presented in Figure 10a. The addition of methanol caused a small change in the absorption of derivative **3**, especially in the long-term spectrum area, and its decrease was observed with increasing methanol concentration (Figure S3). The most likely reason for these changes is the influence of the methanol proton-donor properties on the intramolecular hydrogen bond system in derivative **3**. The measurements of the fluorescence spectra for 4-aminocoumarin and derivative **3** showed that there is a significant difference between them (Figure 10b).

The concentrations of the 4-aminocoumarin and derivative **3** were chosen so that the absorbance (A) at wavelength λ_exc_ = 310 nm would correspond (A = 0.2). This enabled the relative fluorescence intensity for both of the compounds to be compared. It was observed that the fluorescence of derivative **3** was nearly completely extinguished compared to 4-aminocoumarin in chloroform. At a wavelength of λ = 369 nm, the fluorescence intensity of 4-aminocoumarin was more than 80 times stronger than that of the aminocoumarin derivative of the resorcin[4]arene. The most probable reason for such a strong fluorescence quenching of derivative **3** is the nonradiative energy dissipation through the formation of the intramolecular hydrogen bond system in derivative **3** [39,40]. However, no changes were observed in the fluorescence spectrum of derivative **3** after the addition of methanol.

## 3. Materials and Methods

The NMR spectra were achieved using a Avance 400 MHz ultra-shield spectrometer (Bruker, Karlsruhe, Germany). The mass spectra were recorded by electrospray ionisation (ESI) coupled with a TOF analyser (Bruker, Karlsruhe, Germany). The reaction was completed using a Monowave 50 reactor (Anton Paar, Graz, Austria). The UV-Vis spectra were measured using an Carry 60 spectrophotometer (Agilent, Santa Clara, Ca, USA). The fluorescence spectra were carried out using a F-7000 fluorescence spectrophotometer (Hitachi, Japan) All reagents and solvents were obtained from Sigma-Aldrich, Fluka, and Merck and were used without purification. The methoxy derivative of resorcin[4]arene (1) was synthesised according to literature [41].

**Aminocoumarin derivative of resorcin[4]arene** (**3**): The methoxy derivative of resorcin[4]arene (0.112 mmol, 100 mg) and the 4-aminocoumarin (0.448 mmol, 72.5 mg, 4 equivalents) in CHCl_3_ (5 mL) were stirred at 160 °C for 15 min in a Monowave 50 reactor. After cooling to room temperature, the precipitate was filtered, washed slowly with toluene, chloroform, acetone, and methanol, and then dried (135 mg, 89% yield). Compound **3** was obtained as a white solid, m.p. > 300 °C. ^1^H NMR (400 MHz, CDCl_3_) δ = 11.18 (s, 4H, g), 9.67 (s, 4H, f), 7.43 (d, *J* = 8.44 Hz, 4H, k), 7.26–7.24 (m, 4H, m), 7.01 (s, 4H, e), 6.96 (t, *J* = 6.97 Hz, 4H, l), 6.69 (d, *J* = 7.70 Hz, 4H, n), 4.45 (t, *J* = 7.34, 4H, d), 3.76 (d, *J* = 15.77, 4H, h), 3.61 (d, *J* = 15.77 Hz, 4H, h’), 2.12–2.00 (m, 8H, c), 1.50 (m, 4H, b), 1.99 (d, *J* = 6.24 Hz, 24H, a) ppm; ^13^C NMR (100 MHz, CDCl_3_) δ = 167.62, 154.07, 151.88, 149.62, 149.09, 131.19, 124.91, 124.74, 123.80, 122.36, 122.27, 116.64, 114.93, 112.71, 96.08, 42.33, 32.16, 26.24, 23.03, 20.84 ppm. HRMS ESI m/z for C_84_H_84_N_4_O_16_ [M + H]^+^ calcd 1405.5882, found 1405.5889.

## 4. Conclusions

A fast and efficient synthesis of an aminocoumarin derivative of resorcin[4]arene **3** has been presented. This derivative has very low solubility in organic solvents. A conformational analysis was performed based on NMR spectroscopy (CDCl_3_) observation and by quantum-mechanical calculations. Both experimental and analytical methods suggest the formation of a crown-*in* type conformation in CHCl_3_. The conformations of the aminocoumarin derivative of resorcin[4]arene were compared with those of the hydroxycoumarin derivative of resorcin[4]arene, as well as the effects of the substituents in the coumarin molecule on the selectivity of the synthesised coumarin derivative of resorcin[4]arene. The UV-VIS spectrum of derivative **3** in chloroform demonstrated a bathochromic shift (compared to 4-aminocoumarin). By adding a solvent with proton-donor properties to the chloroform solution of derivative **3** (i.e., methanol), a slight reduction in the electron band absorption in the long-wavelength part of the spectrum was produced. Measurements of the fluorescence spectra of derivative **3** showed that it was nearly completely extinguished compared to the fluorescence of 4-aminocoumarin. There was no effect of methanol on the fluorescence spectrum of derivative **3**.

## Figures and Tables

**Table 1:** The energy differences, ∆E (kJ/mol), for the optimised geometry of the crown-*in* and crown-*out* derivative **3** in the gaseous phase, CHCl_3_ and DMSO, calculated by density functional theory (DFT) and DFTB methods.

Method	∆E kJ/mol (E_crown-*out gas*_ − E_crown-*in gas*_)	∆E kJ/mol (E_crown-*out CHCl3*_ − E_crown-*in CHCl3*_)	∆E kJ/mol (E_crown-*out DMSO*_ − E_crown-*in DMSO*_)
DFTB/GFN2-xTB	95.01	28.34	8.01
DFT/B3LYP/6-311G(d,p)	44.84	19.54	10.13

## Supplementary Materials

The following are available online at https://www.mdpi.com/1422-0067/21/17/6160/s1.
