# Supplementary material for: Intramolecular Hydrogen Bond Driven Conformational Selectivity of Coumarin Derivatives of Resorcin[4]arene
Source: Int J Mol Sci. 2020 Aug 26;21(17):6160. doi: 10.3390/ijms21176160 (PMC7503801; doi:10.3390/ijms21176160)
Supplement: Supplementary file 1 [file ijms-21-06160-s001.pdf]

Intramolecular hydrogen bond driven conformational selectivity of coumarin derivatives of resorcin[4]arene

Anna Szafraniec [a] and Waldemar Iwanek [b*]

[a] Faculty of Chemistry, Adam Mickiewicz University, Uniwersytetu Pozna´nskiego 8, 60-614 Poznań, Poland

[b] UTP, University of Science and Technology, Faculty of Chemical Technology and Engineering, Seminaryjna 3, 85-326 Bydgoszcz, Poland: e-mail: *waldemar.iwanek@utp.edu.pl*

Electronic Supporting Information

Table of contents:

1. Figure S1
2. Figure S2
3. Figure S3
4. ^{1}H NMR spectra of 4-aminocoumarin (**2**)
5. ^{13}C NMR spectra of 4-aminocoumarin (**2**)
6. ^{1}H NMR spectra of aminocoumarin derivative of resorcin[4]arene (**3**)
7. ^{13}C NMR spectra of aminocoumarin derivative of resorcin[4]arene (**3**)
8. COSY (^{1}H,^{1}H) spectra of aminocoumarin derivative of resorcin[4]arene (**3**)
9. HSQC spectra of aminocoumarin derivative of resorcin[4]arene (**3**)
10. HMBC spectra of aminocoumarin derivative of resorcin[4]arene (**3**)
11. NOESY spectra of aminocoumarin derivative of resorcin[4]arene (**3**)
12. DFT calculation

1. Figure S1. A ^{1}H-^{13}C HMBC (400 MHZ, $CDCl_3$, 298 K) spectrum fragment showing the coupling of diastereotopic CH_2 group protons (**h,h'**) with aromatic carbon atoms.

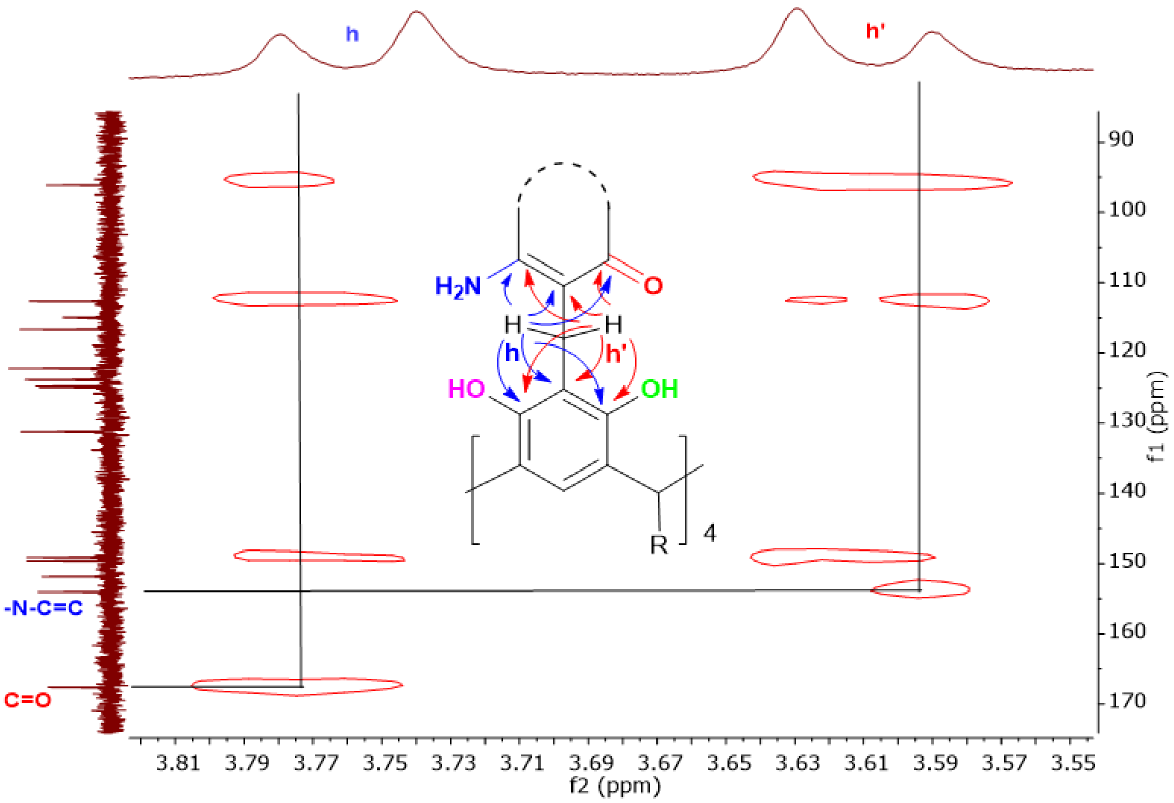

2. Figure S2. A compared the ^{1}H NMR spectra (400 MHz, $CDCl_3$, 298 K) of compound **3** in the presence and absence of THF molecule. The ^{1}H NMR spectrum in $CDCl_3$ is marked in blue. The ^{1}H NMR spectrum with THF molecule in $CDCl_3$ is marked in red.

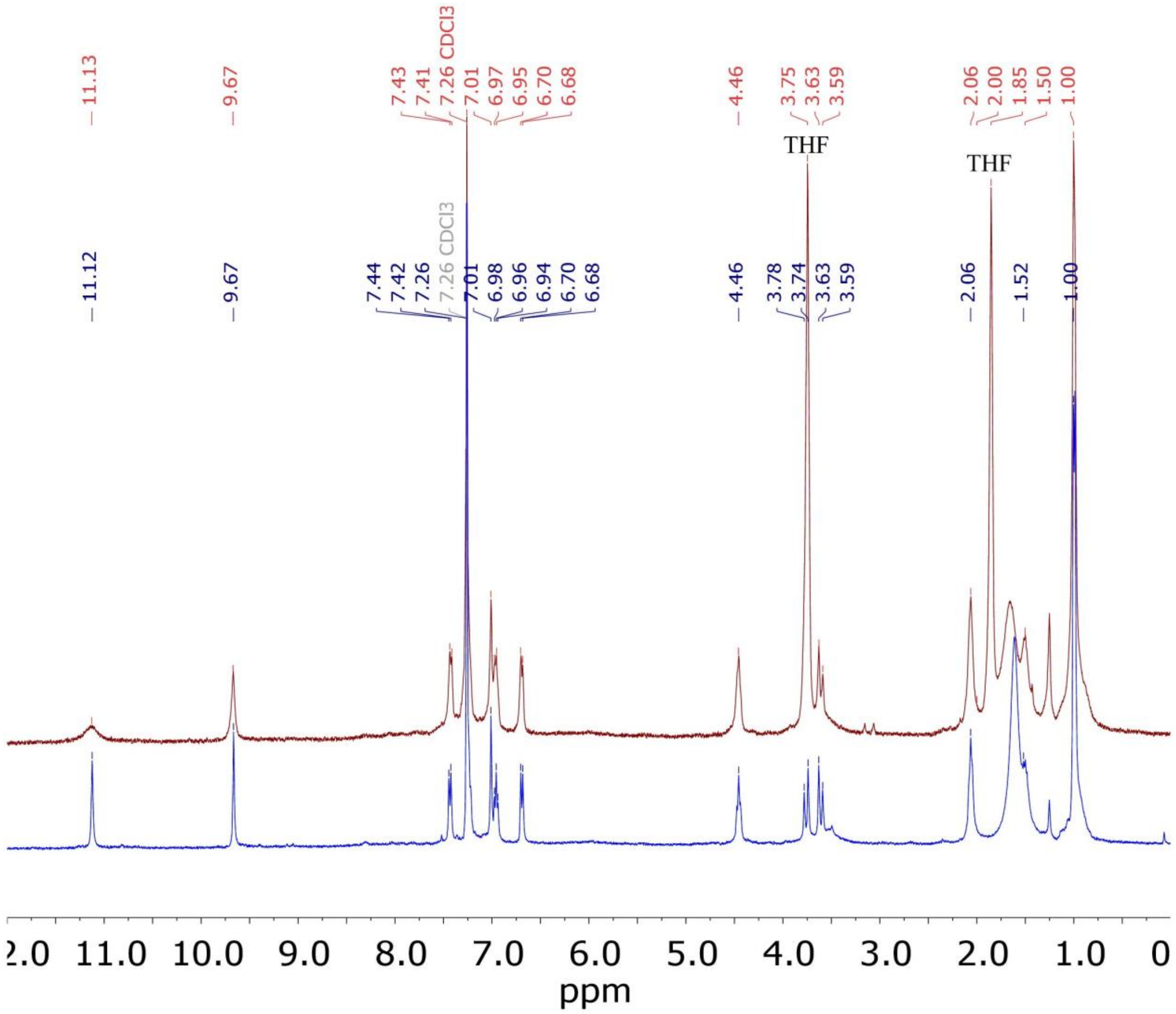

3. Figure S3. Change in the absorption spectrum of derivative 3 (c = 1,84x10^{-6} M) after addition of methanol 10, 20 ... 100 μl, respectively.

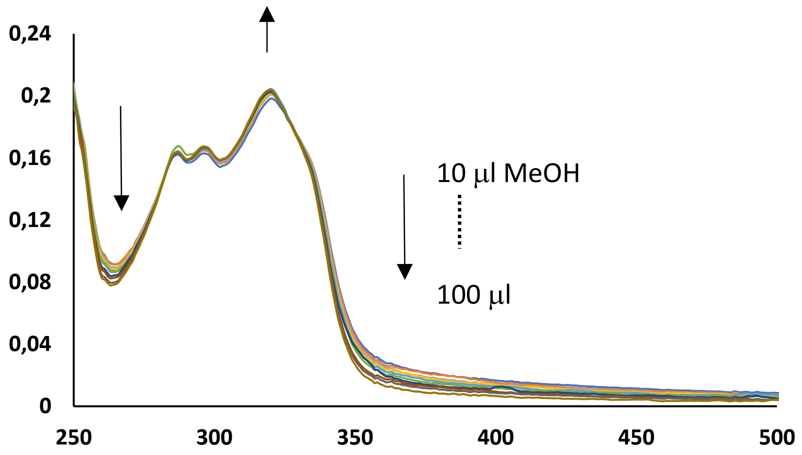

4. ^{1}H NMR spectra (400 MHz, DMSO-d_6, 298 K) of 4-aminocoumarin (**2**)

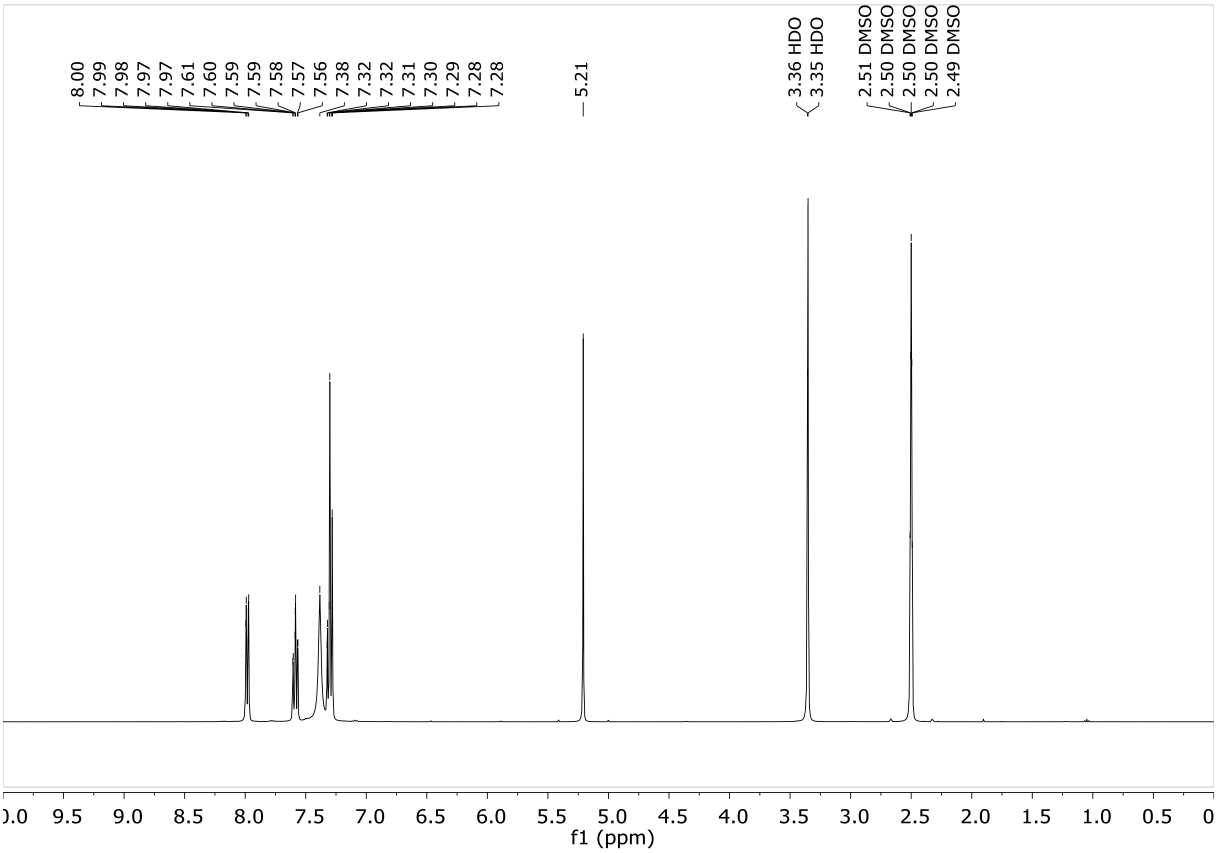

5. ^{13}C NMR spectra (400 MHz, DMSO-d_6, 298 K) of 4-aminocoumarin (**2**)

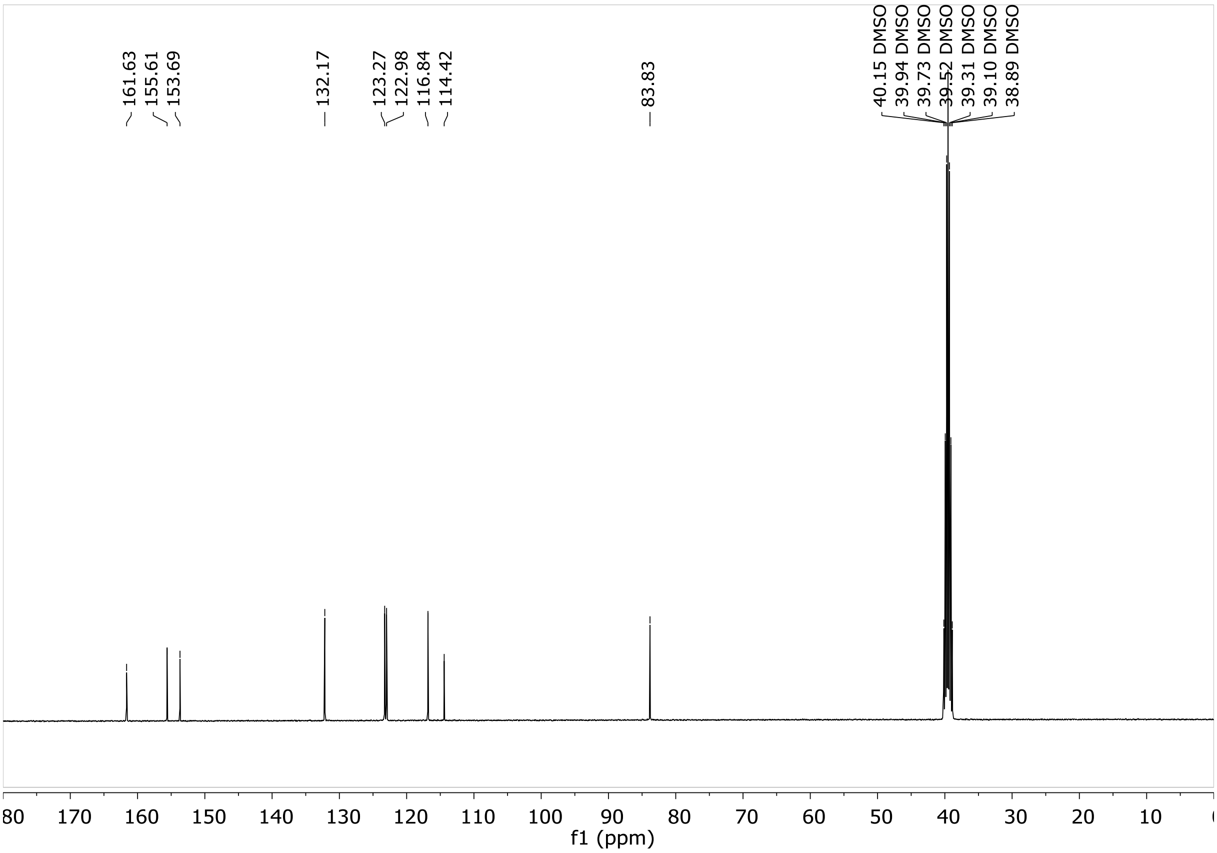

6. ^{1}H NMR spectra (400 MHz, $CDCl_3$, 298 K) of aminocoumarin derivative of resorcin[4]arene (**3**)

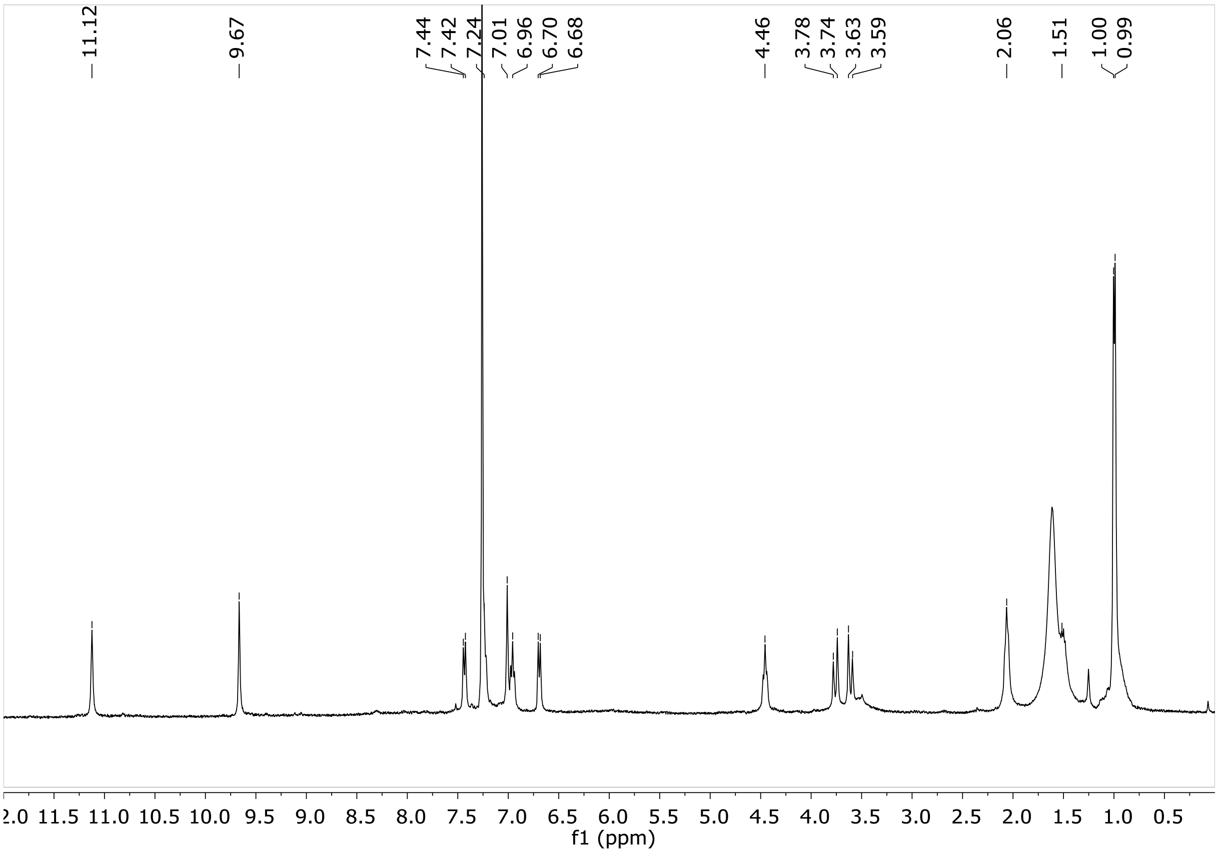

7. ^{13}C NMR spectra (400 MHz, $CDCl_3$, 298 K) of aminocoumarin derivative of resorcin[4]arene (**3**)

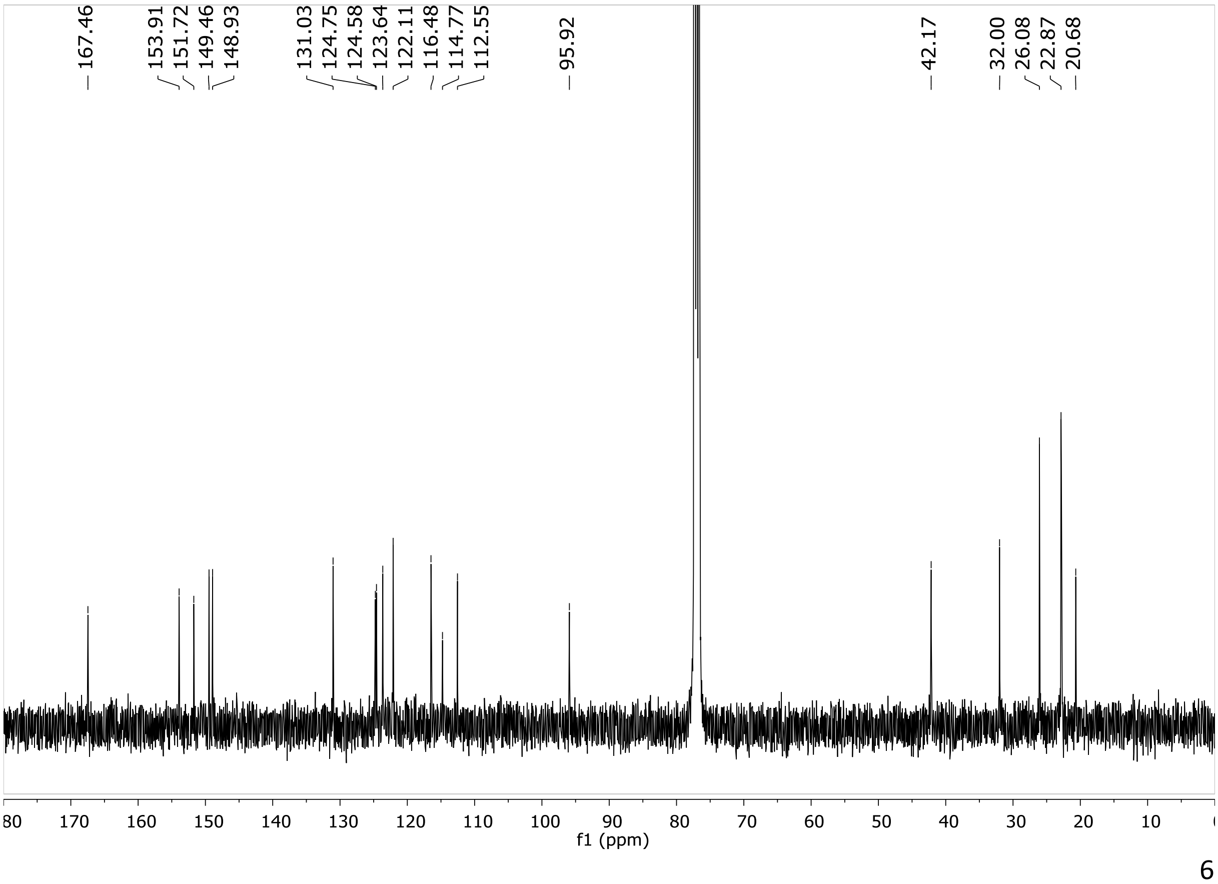

8. COSY (^1H,^1H) spectra (400 MHz, $CDCl_3$, 298 K) of aminocoumarin derivative of resorcin[4]arene (**3**)

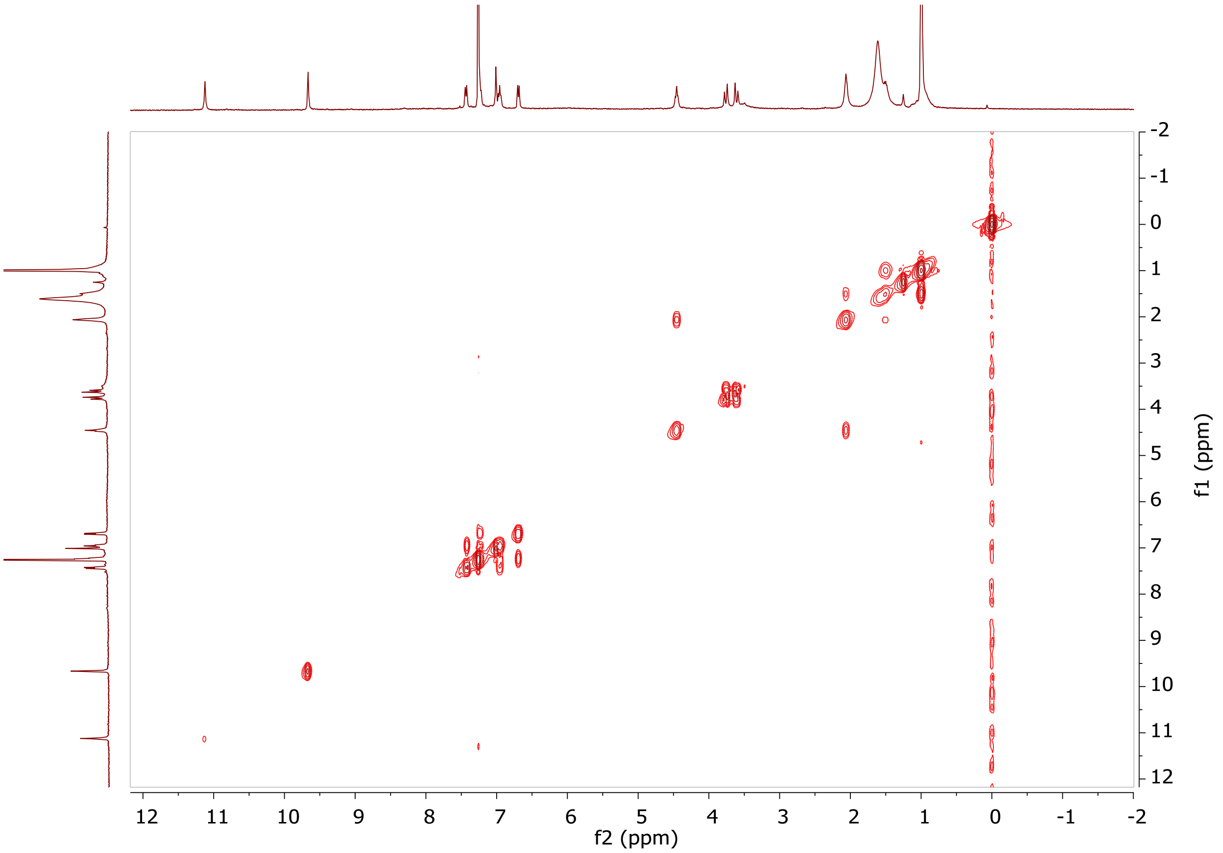

9. HSQC spectra (400 MHz, $CDCl_3$, 298 K) of aminocoumarin derivative of resorcin[4]arene (**3**)

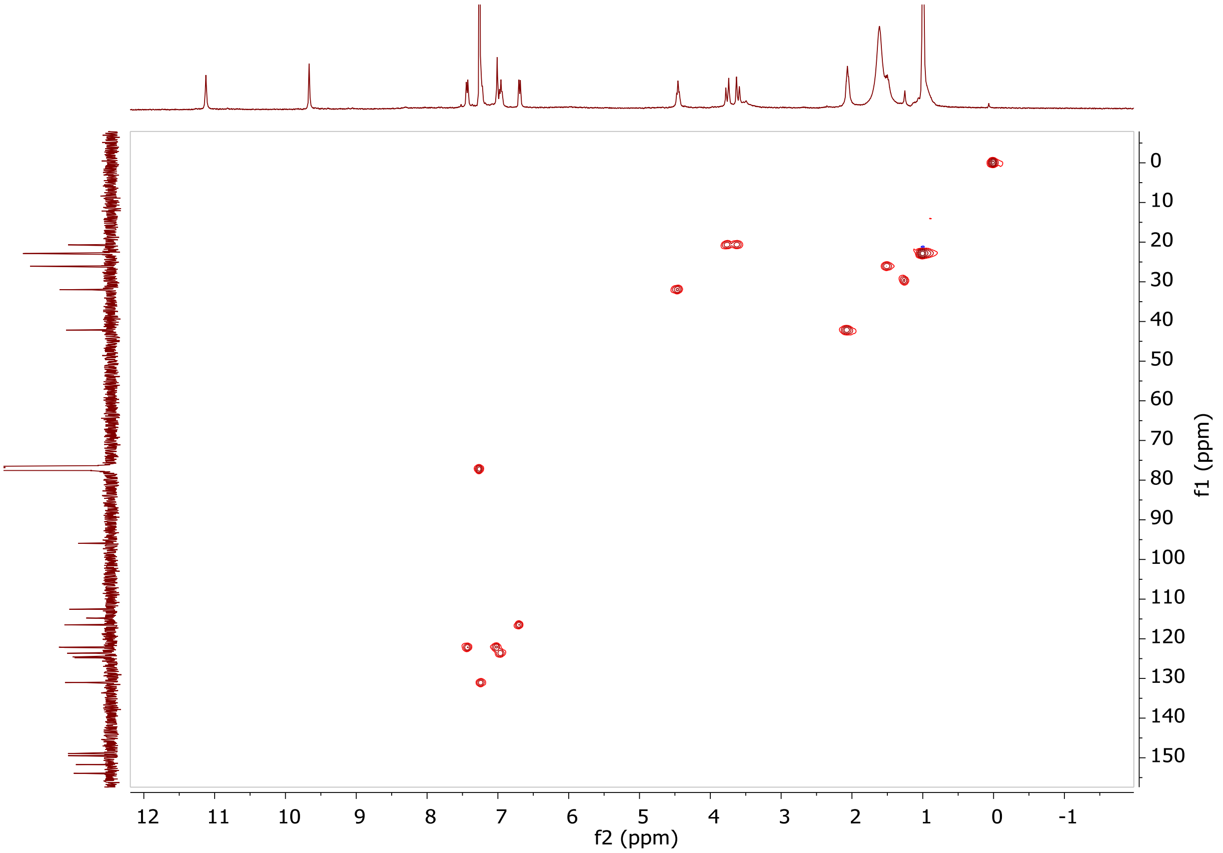

10. HMBC spectra (400 MHz, $CDCl_3$, 298 K) of aminocoumarin derivative of resorcin[4]arene (**3**)

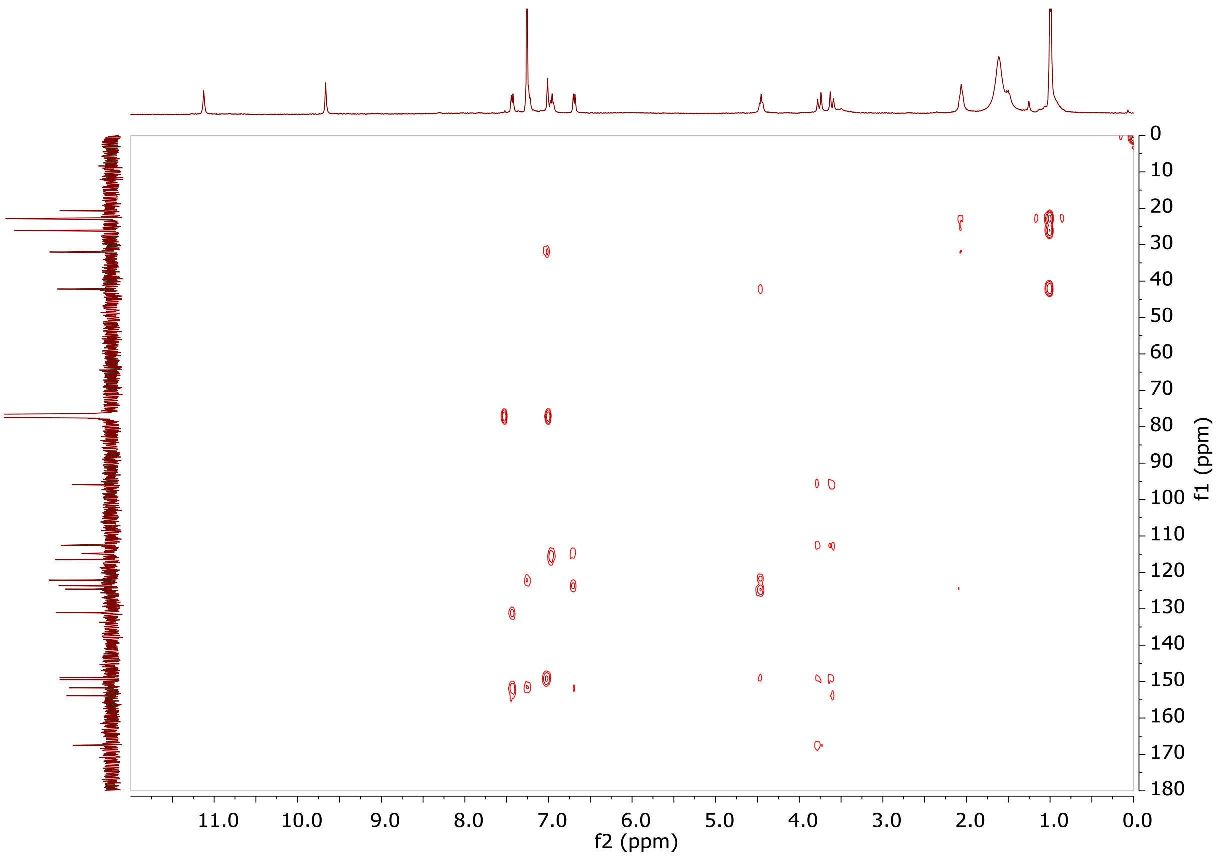

11. NOESY spectra (400 MHz, $CDCl_3$, 298 K) of aminocoumarin derivative of resorcin[4]arene (**3**)

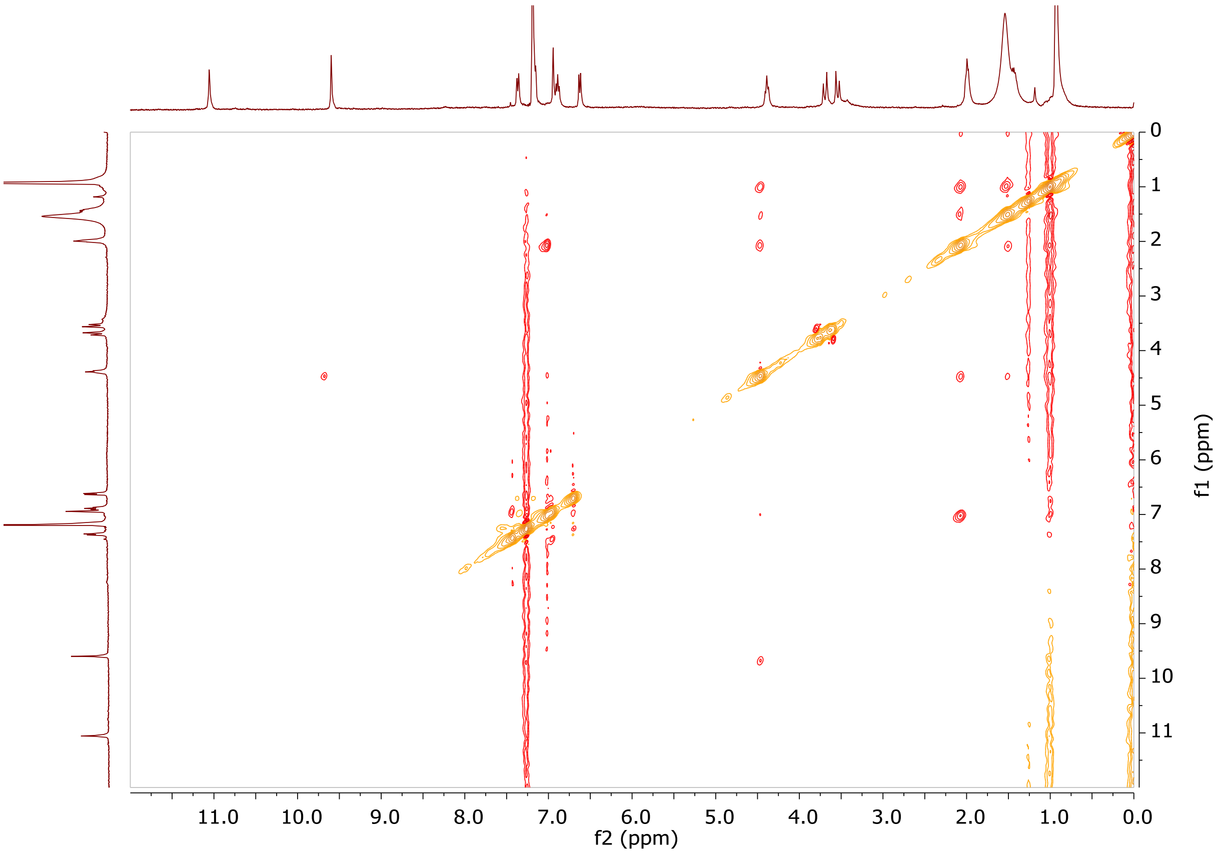

12. DFT calculations

Optimizing the geometry of the compound **3** by semiempirical **DFTB/GFN2-xTB**

Crown-*in* gas phase

E = -299.830827404837 Eh

-1.7972	-2.6565	2.6853	C	0	0	0	0	0	0	0	0	0	0	0	0
-1.9639	-3.4784	1.5643	C	0	0	0	0	0	0	0	0	0	0	0	0
-0.8788	-4.1608	1.0139	C	0	0	0	0	0	0	0	0	0	0	0	0
0.3782	-4.0211	1.6074	C	0	0	0	0	0	0	0	0	0	0	0	0
0.5778	-3.1559	2.6852	C	0	0	0	0	0	0	0	0	0	0	0	0
-0.5186	-2.4707	3.1785	C	0	0	0	0	0	0	0	0	0	0	0	0
-3.2258	-3.6174	1.0510	O	0	0	0	0	0	0	0	0	0	0	0	0
1.4000	-4.7859	1.1135	O	0	0	0	0	0	0	0	0	0	0	0	0
1.9616	-2.9847	3.2851	C	0	0	0	0	0	0	0	0	0	0	0	0
2.6364	-1.7794	2.6596	C	0	0	0	0	0	0	0	0	0	0	0	0
3.4508	-1.9449	1.5340	C	0	0	0	0	0	0	0	0	0	0	0	0
4.1357	-0.8609	0.9843	C	0	0	0	0	0	0	0	0	0	0	0	0
4.0039	0.3948	1.5829	C	0	0	0	0	0	0	0	0	0	0	0	0
3.1530	0.5904	2.6717	C	0	0	0	0	0	0	0	0	0	0	0	0
2.4719	-0.5055	3.1723	C	0	0	0	0	0	0	0	0	0	0	0	0
3.5816	-3.2058	1.0157	O	0	0	0	0	0	0	0	0	0	0	0	0
2.9955	1.9627	3.3016	C	0	0	0	0	0	0	0	0	0	0	0	0
1.7850	2.6413	2.6874	C	0	0	0	0	0	0	0	0	0	0	0	0
1.9503	3.4659	1.5675	C	0	0	0	0	0	0	0	0	0	0	0	0
0.8661	4.1499	1.0178	C	0	0	0	0	0	0	0	0	0	0	0	0
-0.3916	4.0084	1.6096	C	0	0	0	0	0	0	0	0	0	0	0	0
-0.5895	3.1417	2.6851	C	0	0	0	0	0	0	0	0	0	0	0	0
0.5064	2.4568	3.1816	C	0	0	0	0	0	0	0	0	0	0	0	0
3.2124	3.6093	1.0550	O	0	0	0	0	0	0	0	0	0	0	0	0
-1.4126	4.7770	1.1208	O	0	0	0	0	0	0	0	0	0	0	0	0
-1.9649	2.9782	3.3074	C	0	0	0	0	0	0	0	0	0	0	0	0
-2.6441	1.7711	2.6880	C	0	0	0	0	0	0	0	0	0	0	0	0
-3.4602	1.9409	1.5624	C	0	0	0	0	0	0	0	0	0	0	0	0
-4.1458	0.8603	1.0074	C	0	0	0	0	0	0	0	0	0	0	0	0
-4.0139	-0.3986	1.5988	C	0	0	0	0	0	0	0	0	0	0	0	0
-3.1585	-0.6002	2.6825	C	0	0	0	0	0	0	0	0	0	0	0	0
-2.4719	0.4919	3.1848	C	0	0	0	0	0	0	0	0	0	0	0	0
-3.5946	3.2039	1.0503	O	0	0	0	0	0	0	0	0	0	0	0	0
-4.7806	-1.4168	1.1017	O	0	0	0	0	0	0	0	0	0	0	0	0
-3.0047	-1.9772	3.3029	C	0	0	0	0	0	0	0	0	0	0	0	0
1.9376	-2.9385	4.8189	C	0	0	0	0	0	0	0	0	0	0	0	0
2.9842	1.8828	4.8368	C	0	0	0	0	0	0	0	0	0	0	0	0
-1.8916	2.9660	4.8429	C	0	0	0	0	0	0	0	0	0	0	0	0
-2.9952	-1.9089	4.8384	C	0	0	0	0	0	0	0	0	0	0	0	0
3.3337	-2.8309	5.4445	C	0	0	0	0	0	0	0	0	0	0	0	0
3.4364	3.1776	5.5215	C	0	0	0	0	0	0	0	0	0	0	0	0
-3.1912	3.4116	5.5229	C	0	0	0	0	0	0	0	0	0	0	0	0
-3.4326	-3.2136	5.5139	C	0	0	0	0	0	0	0	0	0	0	0	0
-5.0834	1.0446	-0.1702	C	0	0	0	0	0	0	0	0	0	0	0	0
4.7696	1.4158	1.0898	O	0	0	0	0	0	0	0	0	0	0	0	0
-1.0565	-5.0982	-0.1650	C	0	0	0	0	0	0	0	0	0	0	0	0
5.0726	-1.0399	-0.1947	C	0	0	0	0	0	0	0	0	0	0	0	0
-0.8690	-4.4648	-1.5313	C	0	0	0	0	0	0	0	0	0	0	0	0
0.3547	-4.4557	-2.1911	C	0	0	0	0	0	0	0	0	0	0	0	0
0.3761	-4.0437	-3.5841	C	0	0	0	0	0	0	0	0	0	0	0	0
-0.7900	-3.4966	-4.1293	C	0	0	0	0	0	0	0	0	0	0	0	0
-1.9159	-3.3528	-3.3957	O	0	0	0	0	0	0	0	0	0	0	0	0
-2.0095	-3.8838	-2.1392	C	0	0	0	0	0	0	0	0	0	0	0	0
-3.1231	-3.7781	-1.6114	O	0	0	0	0	0	0	0	0	0	0	0	0
1.4863	-4.8915	-1.6279	N	0	0	0	0	0	0	0	0	0	0	0	0
1.4783	-4.2358	-4.4247	C	0	0	0	0	0	0	0	0	0	0	0	0
1.4163	-3.8822	-5.7533	C	0	0	0	0	0	0	0	0	0	0	0	0
0.2655	-3.2940	-6.2698	C	0	0	0	0	0	0	0	0	0	0	0	0
-0.8332	-3.0966	-5.4637	C	0	0	0	0	0	0	0	0	0	0	0	0
4.4421	-0.8485	-1.5615	C	0	0	0	0	0	0	0	0	0	0	0	0
4.4387	0.3766	-2.2189	C	0	0	0	0	0	0	0	0	0	0	0	0
4.0320	0.4011	-3.6134	C	0	0	0	0	0	0	0	0	0	0	0	0
3.4829	-0.7626	-4.1619	C	0	0	0	0	0	0	0	0	0	0	0	0
3.3325	-1.8889	-3.4305	O	0	0	0	0	0	0	0	0	0	0	0	0

3.8594	-1.9862	-2.1727	C	0	0	0	0	0	0	0	0	0	0	0	0
3.7492	-3.1003	-1.6464	O	0	0	0	0	0	0	0	0	0	0	0	0
4.2316	1.5033	-4.4521	C	0	0	0	0	0	0	0	0	0	0	0	0
3.8831	1.4440	-5.7822	C	0	0	0	0	0	0	0	0	0	0	0	0
3.2925	0.2958	-6.3021	C	0	0	0	0	0	0	0	0	0	0	0	0
3.0877	-0.8028	-5.4979	C	0	0	0	0	0	0	0	0	0	0	0	0
4.8763	1.5052	-1.6513	N	0	0	0	0	0	0	0	0	0	0	0	0
1.0445	5.0944	-0.1552	C	0	0	0	0	0	0	0	0	0	0	0	0
0.8565	4.4692	-1.5251	C	0	0	0	0	0	0	0	0	0	0	0	0
0.7775	3.5150	-4.1279	C	0	0	0	0	0	0	0	0	0	0	0	0
1.9036	3.3674	-3.3954	O	0	0	0	0	0	0	0	0	0	0	0	0
1.9971	3.8914	-2.1359	C	0	0	0	0	0	0	0	0	0	0	0	0
3.1104	3.7829	-1.6081	O	0	0	0	0	0	0	0	0	0	0	0	0
-0.3672	4.4642	-2.1849	C	0	0	0	0	0	0	0	0	0	0	0	0
-0.3886	4.0593	-3.5799	C	0	0	0	0	0	0	0	0	0	0	0	0
-1.4983	4.8981	-1.6193	N	0	0	0	0	0	0	0	0	0	0	0	0
-1.4908	4.2554	-4.4195	C	0	0	0	0	0	0	0	0	0	0	0	0
-1.4291	3.9082	-5.7498	C	0	0	0	0	0	0	0	0	0	0	0	0
-0.2782	3.3228	-6.2693	C	0	0	0	0	0	0	0	0	0	0	0	0
0.8206	3.1216	-5.4644	C	0	0	0	0	0	0	0	0	0	0	0	0
-4.4536	0.8588	-1.5382	C	0	0	0	0	0	0	0	0	0	0	0	0
-3.4947	0.7855	-4.1391	C	0	0	0	0	0	0	0	0	0	0	0	0
-3.3447	1.9086	-3.4026	O	0	0	0	0	0	0	0	0	0	0	0	0
-3.8717	1.9997	-2.1443	C	0	0	0	0	0	0	0	0	0	0	0	0
-3.7623	3.1114	-1.6129	O	0	0	0	0	0	0	0	0	0	0	0	0
-4.4498	-0.3631	-2.2013	C	0	0	0	0	0	0	0	0	0	0	0	0
-4.0433	-0.3809	-3.5959	C	0	0	0	0	0	0	0	0	0	0	0	0
-4.8867	-1.4947	-1.6392	N	0	0	0	0	0	0	0	0	0	0	0	0
-4.2426	-1.4792	-4.4398	C	0	0	0	0	0	0	0	0	0	0	0	0
-3.8943	-1.4136	-5.7696	C	0	0	0	0	0	0	0	0	0	0	0	0
-3.3041	-0.2629	-6.2843	C	0	0	0	0	0	0	0	0	0	0	0	0
-3.0996	0.8321	-5.4750	C	0	0	0	0	0	0	0	0	0	0	0	0
3.2009	-2.6021	6.9497	C	0	0	0	0	0	0	0	0	0	0	0	0
4.1788	-4.0746	5.1794	C	0	0	0	0	0	0	0	0	0	0	0	0
-3.6001	-2.9783	7.0151	C	0	0	0	0	0	0	0	0	0	0	0	0
-2.4420	-4.3495	5.2736	C	0	0	0	0	0	0	0	0	0	0	0	0
-2.9494	3.5832	7.0225	C	0	0	0	0	0	0	0	0	0	0	0	0
-4.3329	2.4259	5.2907	C	0	0	0	0	0	0	0	0	0	0	0	0
3.5994	2.9306	7.0211	C	0	0	0	0	0	0	0	0	0	0	0	0
2.4591	4.3261	5.2874	C	0	0	0	0	0	0	0	0	0	0	0	0
-0.3735	-1.7840	3.9937	H	0	0	0	0	0	0	0	0	0	0	0	0
-3.1877	-3.8293	0.0856	H	0	0	0	0	0	0	0	0	0	0	0	0
2.2522	-4.3162	1.2445	H	0	0	0	0	0	0	0	0	0	0	0	0
2.5562	-3.8647	3.0280	H	0	0	0	0	0	0	0	0	0	0	0	0
1.8132	-0.3620	4.0107	H	0	0	0	0	0	0	0	0	0	0	0	0
3.7922	-3.1657	0.0501	H	0	0	0	0	0	0	0	0	0	0	0	0
3.8737	2.5653	3.0485	H	0	0	0	0	0	0	0	0	0	0	0	0
0.3549	1.7777	4.0024	H	0	0	0	0	0	0	0	0	0	0	0	0
3.1740	3.8250	0.0905	H	0	0	0	0	0	0	0	0	0	0	0	0
-2.2663	4.3125	1.2608	H	0	0	0	0	0	0	0	0	0	0	0	0
-2.5662	3.8574	3.0548	H	0	0	0	0	0	0	0	0	0	0	0	0
-1.8024	0.3377	4.0128	H	0	0	0	0	0	0	0	0	0	0	0	0
-3.8065	3.1680	0.0848	H	0	0	0	0	0	0	0	0	0	0	0	0
-4.3228	-2.2732	1.2480	H	0	0	0	0	0	0	0	0	0	0	0	0
-3.8853	-2.5747	3.0464	H	0	0	0	0	0	0	0	0	0	0	0	0
1.4517	-3.8506	5.1751	H	0	0	0	0	0	0	0	0	0	0	0	0
1.3437	-2.0960	5.1712	H	0	0	0	0	0	0	0	0	0	0	0	0
3.6737	1.0862	5.1287	H	0	0	0	0	0	0	0	0	0	0	0	0
1.9979	1.6185	5.2152	H	0	0	0	0	0	0	0	0	0	0	0	0
-1.1009	3.6620	5.1356	H	0	0	0	0	0	0	0	0	0	0	0	0
-1.6218	1.9817	5.2227	H	0	0	0	0	0	0	0	0	0	0	0	0
-3.6962	-1.1232	5.1328	H	0	0	0	0	0	0	0	0	0	0	0	0
-2.0129	-1.6348	5.2205	H	0	0	0	0	0	0	0	0	0	0	0	0
3.8429	-1.9662	5.0050	H	0	0	0	0	0	0	0	0	0	0	0	0
4.4127	3.4638	5.1131	H	0	0	0	0	0	0	0	0	0	0	0	0
-3.4830	4.3842	5.1096	H	0	0	0	0	0	0	0	0	0	0	0	0
-4.4053	-3.5081	5.1028	H	0	0	0	0	0	0	0	0	0	0	0	0
-5.5063	2.0524	-0.1340	H	0	0	0	0	0	0	0	0	0	0	0	0
-5.9084	0.3395	-0.0627	H	0	0	0	0	0	0	0	0	0	0	0	0
4.3116	2.2710	1.2424	H	0	0	0	0	0	0	0	0	0	0	0	0
-2.0630	-5.5243	-0.1323	H	0	0	0	0	0	0	0	0	0	0	0	0
-0.3491	-5.9211	-0.0569	H	0	0	0	0	0	0	0	0	0	0	0	0
5.4949	-2.0480	-0.1628	H	0	0	0	0	0	0	0	0	0	0	0	0
5.8979	-0.3356	-0.0849	H	0	0	0	0	0	0	0	0	0	0	0	0
2.3701	-4.5556	-1.9885	H	0	0	0	0	0	0	0	0	0	0	0	0
1.4708	-5.0469	-0.6227	H	0	0	0	0	0	0	0	0	0	0	0	0
2.3768	-4.6863	-4.0353	H	0	0	0	0	0	0	0	0	0	0	0	0

```
    2.2625   -4.0693   -6.3979 H   0  0  0  0  0  0  0  0  0  0  0  0
    0.2260   -3.0026   -7.3085 H   0  0  0  0  0  0  0  0  0  0  0  0
   -1.7406   -2.6538   -5.8396 H   0  0  0  0  0  0  0  0  0  0  0  0
    4.6841    2.3997   -4.0598 H   0  0  0  0  0  0  0  0  0  0  0  0
    4.0759    2.2899   -6.4255 H   0  0  0  0  0  0  0  0  0  0  0  0
    3.0050    0.2588   -7.3420 H   0  0  0  0  0  0  0  0  0  0  0  0
    2.6430   -1.7082   -5.8763 H   0  0  0  0  0  0  0  0  0  0  0  0
    4.5469    2.3921   -2.0099 H   0  0  0  0  0  0  0  0  0  0  0  0
    5.0265    1.4874   -0.6452 H   0  0  0  0  0  0  0  0  0  0  0  0
    2.0511    5.5200   -0.1200 H   0  0  0  0  0  0  0  0  0  0  0  0
    0.3373    5.9168   -0.0420 H   0  0  0  0  0  0  0  0  0  0  0  0
   -2.3827    4.5648   -1.9805 H   0  0  0  0  0  0  0  0  0  0  0  0
   -1.4829    5.0468   -0.6130 H   0  0  0  0  0  0  0  0  0  0  0  0
   -2.3893    4.7040   -4.0277 H   0  0  0  0  0  0  0  0  0  0  0  0
   -2.2754    4.0982   -6.3934 H   0  0  0  0  0  0  0  0  0  0  0  0
   -0.2389    3.0364   -7.3095 H   0  0  0  0  0  0  0  0  0  0  0  0
    1.7280    2.6809   -5.8426 H   0  0  0  0  0  0  0  0  0  0  0  0
   -4.5574   -2.3798   -2.0023 H   0  0  0  0  0  0  0  0  0  0  0  0
   -5.0379   -1.4818   -0.6332 H   0  0  0  0  0  0  0  0  0  0  0  0
   -4.6948   -2.3776   -4.0516 H   0  0  0  0  0  0  0  0  0  0  0  0
   -4.0871   -2.2565   -6.4169 H   0  0  0  0  0  0  0  0  0  0  0  0
   -3.0167   -0.2209   -7.3240 H   0  0  0  0  0  0  0  0  0  0  0  0
   -2.6552    1.7394   -5.8492 H   0  0  0  0  0  0  0  0  0  0  0  0
    2.6367   -1.6947    7.1553 H   0  0  0  0  0  0  0  0  0  0  0  0
    2.6867   -3.4398    7.4169 H   0  0  0  0  0  0  0  0  0  0  0  0
    4.1813   -2.5033    7.4099 H   0  0  0  0  0  0  0  0  0  0  0  0
    3.6548   -4.9698    5.5092 H   0  0  0  0  0  0  0  0  0  0  0  0
    4.4105   -4.1749    4.1226 H   0  0  0  0  0  0  0  0  0  0  0  0
    5.1199   -4.0102    5.7211 H   0  0  0  0  0  0  0  0  0  0  0  0
   -2.6515   -2.6858    7.4611 H   0  0  0  0  0  0  0  0  0  0  0  0
   -4.3267   -2.1911    7.2050 H   0  0  0  0  0  0  0  0  0  0  0  0
   -3.9427   -3.8851    7.5080 H   0  0  0  0  0  0  0  0  0  0  0  0
   -2.3706   -4.5907    4.2171 H   0  0  0  0  0  0  0  0  0  0  0  0
   -1.4512   -4.0707    5.6260 H   0  0  0  0  0  0  0  0  0  0  0  0
   -2.7587   -5.2423    5.8088 H   0  0  0  0  0  0  0  0  0  0  0  0
   -2.6569    2.6353    7.4702 H   0  0  0  0  0  0  0  0  0  0  0  0
   -2.1598    4.3086    7.2068 H   0  0  0  0  0  0  0  0  0  0  0  0
   -3.8534    3.9292    7.5181 H   0  0  0  0  0  0  0  0  0  0  0  0
   -4.5702    2.3417    4.2342 H   0  0  0  0  0  0  0  0  0  0  0  0
   -4.0618    1.4385    5.6585 H   0  0  0  0  0  0  0  0  0  0  0  0
   -5.2258    2.7550    5.8183 H   0  0  0  0  0  0  0  0  0  0  0  0
    2.6468    2.6466    7.4641 H   0  0  0  0  0  0  0  0  0  0  0  0
    4.3156    2.1330    7.2065 H   0  0  0  0  0  0  0  0  0  0  0  0
    3.9523    3.8300    7.5204 H   0  0  0  0  0  0  0  0  0  0  0  0
    2.3812    4.5663    4.2311 H   0  0  0  0  0  0  0  0  0  0  0  0
    1.4682    4.0604    5.6499 H   0  0  0  0  0  0  0  0  0  0  0  0
    2.7913    5.2158    5.8184 H   0  0  0  0  0  0  0  0  0  0  0  0
  1  6  1  0  0  0  0
  1 35  1  0  0  0  0
  2  1  1  0  0  0  0
  3  4  1  0  0  0  0
  3  2  1  0  0  0  0
  4  5  1  0  0  0  0
  5  6  1  0  0  0  0
  5  9  1  0  0  0  0
  6105  1  0  0  0  0
  7  2  1  0  0  0  0
  8107  1  0  0  0  0
  8  4  1  0  0  0  0
  9108  1  0  0  0  0
  9 36  1  0  0  0  0
 10 15  1  0  0  0  0
 10  9  1  0  0  0  0
 11 10  1  0  0  0  0
 12 13  1  0  0  0  0
 12 11  1  0  0  0  0
 13 14  1  0  0  0  0
 14 15  1  0  0  0  0
 14 17  1  0  0  0  0
 15109  1  0  0  0  0
 16 11  1  0  0  0  0
 17111  1  0  0  0  0
 17 37  1  0  0  0  0
 18 23  1  0  0  0  0
 18 17  1  0  0  0  0
 19 18  1  0  0  0  0
 20 21  1  0  0  0  0
```

20 19 1 0 0 0 0
21 22 1 0 0 0 0
22 23 1 0 0 0 0
22 26 1 0 0 0 0
23112 1 0 0 0 0
24 19 1 0 0 0 0
25114 1 0 0 0 0
25 21 1 0 0 0 0
26115 1 0 0 0 0
26 38 1 0 0 0 0
27 32 1 0 0 0 0
27 26 1 0 0 0 0
28 27 1 0 0 0 0
29 30 1 0 0 0 0
29 28 1 0 0 0 0
30 31 1 0 0 0 0
31 32 1 0 0 0 0
31 35 1 0 0 0 0
32116 1 0 0 0 0
33 28 1 0 0 0 0
34118 1 0 0 0 0
34 30 1 0 0 0 0
35119 1 0 0 0 0
35 39 1 0 0 0 0
36120 1 0 0 0 0
36121 1 0 0 0 0
36 40 1 0 0 0 0
37122 1 0 0 0 0
37123 1 0 0 0 0
37 41 1 0 0 0 0
38124 1 0 0 0 0
38125 1 0 0 0 0
38 42 1 0 0 0 0
39126 1 0 0 0 0
39127 1 0 0 0 0
39 43 1 0 0 0 0
40 97 1 0 0 0 0
41103 1 0 0 0 0
42101 1 0 0 0 0
43 99 1 0 0 0 0
44133 1 0 0 0 0
44132 1 0 0 0 0
44 29 1 0 0 0 0
45134 1 0 0 0 0
45 13 1 0 0 0 0
46136 1 0 0 0 0
46135 1 0 0 0 0
46 3 1 0 0 0 0
47138 1 0 0 0 0
47137 1 0 0 0 0
47 12 1 0 0 0 0
48 46 1 0 0 0 0
49 55 1 0 0 0 0
49 48 1 0 0 0 0
50 49 1 0 0 0 0
51 50 1 0 0 0 0
51 52 1 0 0 0 0
52 53 1 0 0 0 0
53 48 1 0 0 0 0
53 54 1 0 0 0 0
55140 1 0 0 0 0
56141 1 0 0 0 0
56 50 1 0 0 0 0
57 56 1 0 0 0 0
58 57 1 0 0 0 0
58 59 1 0 0 0 0
59 51 1 0 0 0 0
60 47 1 0 0 0 0
61 71 1 0 0 0 0
61 60 1 0 0 0 0
62 61 1 0 0 0 0
63 62 1 0 0 0 0
63 64 1 0 0 0 0
64 65 1 0 0 0 0
65 60 1 0 0 0 0
65 66 1 0 0 0 0
67145 1 0 0 0 0

67 62 1 0 0 0 0
68 67 1 0 0 0 0
69 68 1 0 0 0 0
69 70 1 0 0 0 0
70 63 1 0 0 0 0
71150 1 0 0 0 0
72152 1 0 0 0 0
72151 1 0 0 0 0
72 20 1 0 0 0 0
73 72 1 0 0 0 0
74 79 1 0 0 0 0
74 75 1 0 0 0 0
75 76 1 0 0 0 0
76 73 1 0 0 0 0
76 77 1 0 0 0 0
78 80 1 0 0 0 0
78 73 1 0 0 0 0
79 78 1 0 0 0 0
80154 1 0 0 0 0
81155 1 0 0 0 0
81 79 1 0 0 0 0
82 81 1 0 0 0 0
83 82 1 0 0 0 0
83 84 1 0 0 0 0
84 74 1 0 0 0 0
85 44 1 0 0 0 0
86 91 1 0 0 0 0
86 87 1 0 0 0 0
87 88 1 0 0 0 0
88 85 1 0 0 0 0
88 89 1 0 0 0 0
90 92 1 0 0 0 0
90 85 1 0 0 0 0
91 90 1 0 0 0 0
92160 1 0 0 0 0
93161 1 0 0 0 0
93 91 1 0 0 0 0
94 93 1 0 0 0 0
95 94 1 0 0 0 0
95 96 1 0 0 0 0
96 86 1 0 0 0 0
97165 1 0 0 0 0
97166 1 0 0 0 0
97167 1 0 0 0 0
98 40 1 0 0 0 0
98168 1 0 0 0 0
98170 1 0 0 0 0
99172 1 0 0 0 0
99171 1 0 0 0 0
99173 1 0 0 0 0
100 43 1 0 0 0 0
100175 1 0 0 0 0
100176 1 0 0 0 0
101178 1 0 0 0 0
101177 1 0 0 0 0
101179 1 0 0 0 0
102 42 1 0 0 0 0
102181 1 0 0 0 0
102182 1 0 0 0 0
103184 1 0 0 0 0
103183 1 0 0 0 0
103185 1 0 0 0 0
104 41 1 0 0 0 0
104187 1 0 0 0 0
104188 1 0 0 0 0
106 7 1 0 0 0 0
110 16 1 0 0 0 0
113 24 1 0 0 0 0
117 33 1 0 0 0 0
128 40 1 0 0 0 0
129 41 1 0 0 0 0
130 42 1 0 0 0 0
131 43 1 0 0 0 0
139 55 1 0 0 0 0
142 57 1 0 0 0 0
143 58 1 0 0 0 0
144 59 1 0 0 0 0

```
146 68  1  0  0  0  0
147 69  1  0  0  0  0
148 70  1  0  0  0  0
149 71  1  0  0  0  0
153 80  1  0  0  0  0
156 82  1  0  0  0  0
157 83  1  0  0  0  0
158 84  1  0  0  0  0
159 92  1  0  0  0  0
162 94  1  0  0  0  0
163 95  1  0  0  0  0
164 96  1  0  0  0  0
169 98  1  0  0  0  0
174100  1  0  0  0  0
180102  1  0  0  0  0
186104  1  0  0  0  0
M  END
```

Crown-*in*-$CHCl_3$

E = -299.924051649395 Eh

```
188200  0     0  0            999 V2000
   -1.7765    -2.6766     2.6930 C   0  0  0  0  0  0  0  0  0  0  0  0
   -1.9378    -3.5109     1.5809 C   0  0  0  0  0  0  0  0  0  0  0  0
   -0.8502    -4.1846     1.0253 C   0  0  0  0  0  0  0  0  0  0  0  0
    0.4094    -4.0206     1.6084 C   0  0  0  0  0  0  0  0  0  0  0  0
    0.6046    -3.1526     2.6849 C   0  0  0  0  0  0  0  0  0  0  0  0
   -0.4979    -2.4789     3.1824 C   0  0  0  0  0  0  0  0  0  0  0  0
   -3.2067    -3.6739     1.0786 O   0  0  0  0  0  0  0  0  0  0  0  0
    1.4390    -4.7723     1.1022 O   0  0  0  0  0  0  0  0  0  0  0  0
    1.9872    -2.9714     3.2879 C   0  0  0  0  0  0  0  0  0  0  0  0
    2.6563    -1.7592     2.6681 C   0  0  0  0  0  0  0  0  0  0  0  0
    3.4803    -1.9196     1.5490 C   0  0  0  0  0  0  0  0  0  0  0  0
    4.1550    -0.8333     0.9920 C   0  0  0  0  0  0  0  0  0  0  0  0
    4.0005     0.4251     1.5805 C   0  0  0  0  0  0  0  0  0  0  0  0
    3.1501     0.6168     2.6706 C   0  0  0  0  0  0  0  0  0  0  0  0
    2.4807    -0.4848     3.1768 C   0  0  0  0  0  0  0  0  0  0  0  0
    3.6337    -3.1875     1.0412 O   0  0  0  0  0  0  0  0  0  0  0  0
    2.9846     1.9892     3.3011 C   0  0  0  0  0  0  0  0  0  0  0  0
    1.7681     2.6618     2.6909 C   0  0  0  0  0  0  0  0  0  0  0  0
    1.9274     3.4983     1.5795 C   0  0  0  0  0  0  0  0  0  0  0  0
    0.8399     4.1729     1.0251 C   0  0  0  0  0  0  0  0  0  0  0  0
   -0.4199     4.0071     1.6074 C   0  0  0  0  0  0  0  0  0  0  0  0
   -0.6124     3.1382     2.6821 C   0  0  0  0  0  0  0  0  0  0  0  0
    0.4898     2.4643     3.1816 C   0  0  0  0  0  0  0  0  0  0  0  0
    3.1961     3.6659     1.0776 O   0  0  0  0  0  0  0  0  0  0  0  0
   -1.4495     4.7618     1.1063 O   0  0  0  0  0  0  0  0  0  0  0  0
   -1.9859     2.9644     3.3087 C   0  0  0  0  0  0  0  0  0  0  0  0
   -2.6597     1.7517     2.6928 C   0  0  0  0  0  0  0  0  0  0  0  0
   -3.4871     1.9164     1.5753 C   0  0  0  0  0  0  0  0  0  0  0  0
   -4.1621     0.8330     1.0133 C   0  0  0  0  0  0  0  0  0  0  0  0
   -4.0070    -0.4286     1.5947 C   0  0  0  0  0  0  0  0  0  0  0  0
   -3.1512    -0.6257     2.6790 C   0  0  0  0  0  0  0  0  0  0  0  0
   -2.4756     0.4722     3.1854 C   0  0  0  0  0  0  0  0  0  0  0  0
   -3.6461     3.1867     1.0751 O   0  0  0  0  0  0  0  0  0  0  0  0
   -4.7591    -1.4550     1.0833 O   0  0  0  0  0  0  0  0  0  0  0  0
   -2.9900    -2.0008     3.3043 C   0  0  0  0  0  0  0  0  0  0  0  0
    1.9576    -2.9360     4.8224 C   0  0  0  0  0  0  0  0  0  0  0  0
    2.9803     1.9102     4.8371 C   0  0  0  0  0  0  0  0  0  0  0  0
   -1.9073     2.9559     4.8448 C   0  0  0  0  0  0  0  0  0  0  0  0
   -2.9876    -1.9272     4.8405 C   0  0  0  0  0  0  0  0  0  0  0  0
    3.3515    -2.8614     5.4585 C   0  0  0  0  0  0  0  0  0  0  0  0
    3.4567     3.1984     5.5191 C   0  0  0  0  0  0  0  0  0  0  0  0
   -3.1964     3.4289     5.5277 C   0  0  0  0  0  0  0  0  0  0  0  0
   -3.4643    -3.2181     5.5171 C   0  0  0  0  0  0  0  0  0  0  0  0
   -5.1143     1.0164    -0.1535 C   0  0  0  0  0  0  0  0  0  0  0  0
    4.7509     1.4542     1.0719 O   0  0  0  0  0  0  0  0  0  0  0  0
   -1.0270    -5.1391    -0.1407 C   0  0  0  0  0  0  0  0  0  0  0  0
    5.1053    -1.0116    -0.1771 C   0  0  0  0  0  0  0  0  0  0  0  0
   -0.8544    -4.5245    -1.5180 C   0  0  0  0  0  0  0  0  0  0  0  0
    0.3570    -4.5381    -2.2022 C   0  0  0  0  0  0  0  0  0  0  0  0
    0.3656    -4.1289    -3.5951 C   0  0  0  0  0  0  0  0  0  0  0  0
```

-0.8047	-3.5777	-4.1276	C	0	0	0	0	0	0	0	0	0	0	0	0	0
-1.9252	-3.4293	-3.3748	O	0	0	0	0	0	0	0	0	0	0	0	0	0
-1.9982	-3.9446	-2.1170	C	0	0	0	0	0	0	0	0	0	0	0	0	0
-3.1108	-3.8294	-1.5748	O	0	0	0	0	0	0	0	0	0	0	0	0	0
1.4890	-4.9968	-1.6520	N	0	0	0	0	0	0	0	0	0	0	0	0	0
1.4598	-4.3130	-4.4508	C	0	0	0	0	0	0	0	0	0	0	0	0	0
1.3818	-3.9489	-5.7757	C	0	0	0	0	0	0	0	0	0	0	0	0	0
0.2224	-3.3637	-6.2783	C	0	0	0	0	0	0	0	0	0	0	0	0	0
-0.8687	-3.1740	-5.4596	C	0	0	0	0	0	0	0	0	0	0	0	0	0
4.4895	-0.8352	-1.5531	C	0	0	0	0	0	0	0	0	0	0	0	0	0
4.5081	0.3771	-2.2359	C	0	0	0	0	0	0	0	0	0	0	0	0	0
4.1002	0.3883	-3.6291	C	0	0	0	0	0	0	0	0	0	0	0	0	0
3.5434	-0.7791	-4.1624	C	0	0	0	0	0	0	0	0	0	0	0	0	0
3.3897	-1.8994	-3.4106	O	0	0	0	0	0	0	0	0	0	0	0	0	0
3.9053	-1.9760	-2.1534	C	0	0	0	0	0	0	0	0	0	0	0	0	0
3.7869	-3.0890	-1.6123	O	0	0	0	0	0	0	0	0	0	0	0	0	0
4.2912	1.4816	-4.4844	C	0	0	0	0	0	0	0	0	0	0	0	0	0
3.9280	1.4057	-5.8098	C	0	0	0	0	0	0	0	0	0	0	0	0	0
3.3361	0.2500	-6.3129	C	0	0	0	0	0	0	0	0	0	0	0	0	0
3.1396	-0.8403	-5.4946	C	0	0	0	0	0	0	0	0	0	0	0	0	0
4.9706	1.5062	-1.6832	N	0	0	0	0	0	0	0	0	0	0	0	0	0
1.0166	5.1340	-0.1354	C	0	0	0	0	0	0	0	0	0	0	0	0	0
0.8425	4.5275	-1.5159	C	0	0	0	0	0	0	0	0	0	0	0	0	0
0.7910	3.5946	-4.1301	C	0	0	0	0	0	0	0	0	0	0	0	0	0
1.9115	3.4407	-3.3785	O	0	0	0	0	0	0	0	0	0	0	0	0	0
1.9854	3.9491	-2.1181	C	0	0	0	0	0	0	0	0	0	0	0	0	0
3.0978	3.8298	-1.5763	O	0	0	0	0	0	0	0	0	0	0	0	0	0
-0.3691	4.5468	-2.1997	C	0	0	0	0	0	0	0	0	0	0	0	0	0
-0.3785	4.1448	-3.5947	C	0	0	0	0	0	0	0	0	0	0	0	0	0
-1.4999	5.0047	-1.6465	N	0	0	0	0	0	0	0	0	0	0	0	0	0
-1.4726	4.3347	-4.4491	C	0	0	0	0	0	0	0	0	0	0	0	0	0
-1.3954	3.9771	-5.7760	C	0	0	0	0	0	0	0	0	0	0	0	0	0
-0.2369	3.3929	-6.2817	C	0	0	0	0	0	0	0	0	0	0	0	0	0
0.8541	3.1976	-5.4642	C	0	0	0	0	0	0	0	0	0	0	0	0	0
-4.4997	0.8470	-1.5310	C	0	0	0	0	0	0	0	0	0	0	0	0	0
-3.5563	0.8056	-4.1414	C	0	0	0	0	0	0	0	0	0	0	0	0	0
-3.4036	1.9225	-3.3842	O	0	0	0	0	0	0	0	0	0	0	0	0	0
-3.9180	1.9918	-2.1260	C	0	0	0	0	0	0	0	0	0	0	0	0	0
-3.8009	3.1021	-1.5793	O	0	0	0	0	0	0	0	0	0	0	0	0	0
-4.5166	-0.3620	-2.2195	C	0	0	0	0	0	0	0	0	0	0	0	0	0
-4.1101	-0.3655	-3.6132	C	0	0	0	0	0	0	0	0	0	0	0	0	0
-4.9763	-1.4946	-1.6719	N	0	0	0	0	0	0	0	0	0	0	0	0	0
-4.3001	-1.4550	-4.4737	C	0	0	0	0	0	0	0	0	0	0	0	0	0
-3.9387	-1.3717	-5.7992	C	0	0	0	0	0	0	0	0	0	0	0	0	0
-3.3498	-0.2123	-6.2973	C	0	0	0	0	0	0	0	0	0	0	0	0	0
-3.1544	0.8742	-5.4738	C	0	0	0	0	0	0	0	0	0	0	0	0	0
3.2106	-2.6663	6.9685	C	0	0	0	0	0	0	0	0	0	0	0	0	0
4.1840	-4.1103	5.1729	C	0	0	0	0	0	0	0	0	0	0	0	0	0
-3.6408	-2.9738	7.0163	C	0	0	0	0	0	0	0	0	0	0	0	0	0
-2.4988	-4.3782	5.2884	C	0	0	0	0	0	0	0	0	0	0	0	0	0
-2.9441	3.6124	7.0248	C	0	0	0	0	0	0	0	0	0	0	0	0	0
-4.3535	2.4573	5.3102	C	0	0	0	0	0	0	0	0	0	0	0	0	0
3.6344	2.9470	7.0170	C	0	0	0	0	0	0	0	0	0	0	0	0	0
2.4904	4.3591	5.2971	C	0	0	0	0	0	0	0	0	0	0	0	0	0
-0.3565	-1.7923	3.9972	H	0	0	0	0	0	0	0	0	0	0	0	0	0
-3.1707	-3.8817	0.1119	H	0	0	0	0	0	0	0	0	0	0	0	0	0
2.2890	-4.3028	1.2442	H	0	0	0	0	0	0	0	0	0	0	0	0	0
2.5882	-3.8453	3.0252	H	0	0	0	0	0	0	0	0	0	0	0	0	0
1.8230	-0.3446	4.0154	H	0	0	0	0	0	0	0	0	0	0	0	0	0
3.8368	-3.1496	0.0736	H	0	0	0	0	0	0	0	0	0	0	0	0	0
3.8577	2.5965	3.0416	H	0	0	0	0	0	0	0	0	0	0	0	0	0
0.3433	1.7843	4.0013	H	0	0	0	0	0	0	0	0	0	0	0	0	0
3.1591	3.8758	0.1114	H	0	0	0	0	0	0	0	0	0	0	0	0	0
-2.3007	4.2972	1.2579	H	0	0	0	0	0	0	0	0	0	0	0	0	0
-2.5935	3.8384	3.0532	H	0	0	0	0	0	0	0	0	0	0	0	0	0
-1.8057	0.3214	4.0126	H	0	0	0	0	0	0	0	0	0	0	0	0	0
-3.8512	3.1534	0.1077	H	0	0	0	0	0	0	0	0	0	0	0	0	0
-4.3032	-2.3093	1.2435	H	0	0	0	0	0	0	0	0	0	0	0	0	0
-3.8656	-2.6035	3.0430	H	0	0	0	0	0	0	0	0	0	0	0	0	0
1.4539	-3.8426	5.1660	H	0	0	0	0	0	0	0	0	0	0	0	0	0
1.3788	-2.0849	5.1761	H	0	0	0	0	0	0	0	0	0	0	0	0	0
3.6612	1.1053	5.1228	H	0	0	0	0	0	0	0	0	0	0	0	0	0
1.9918	1.6621	5.2181	H	0	0	0	0	0	0	0	0	0	0	0	0	0
-1.1039	3.6388	5.1305	H	0	0	0	0	0	0	0	0	0	0	0	0	0
-1.6563	1.9675	5.2242	H	0	0	0	0	0	0	0	0	0	0	0	0	0
-3.6700	-1.1235	5.1265	H	0	0	0	0	0	0	0	0	0	0	0	0	0
-2.0000	-1.6799	5.2244	H	0	0	0	0	0	0	0	0	0	0	0	0	0

```
    3.8751   -1.9943    5.0406 H   0  0  0  0  0  0  0  0  0  0  0  0
    4.4317    3.4738    5.0991 H   0  0  0  0  0  0  0  0  0  0  0  0
   -3.4762    4.4015    5.1054 H   0  0  0  0  0  0  0  0  0  0  0  0
   -4.4399   -3.4905    5.0968 H   0  0  0  0  0  0  0  0  0  0  0  0
   -5.5434    2.0212   -0.1047 H   0  0  0  0  0  0  0  0  0  0  0  0
   -5.9328    0.3050   -0.0407 H   0  0  0  0  0  0  0  0  0  0  0  0
    4.2941    2.3074    1.2363 H   0  0  0  0  0  0  0  0  0  0  0  0
   -2.0310   -5.5704   -0.0950 H   0  0  0  0  0  0  0  0  0  0  0  0
   -0.3143   -5.9560   -0.0249 H   0  0  0  0  0  0  0  0  0  0  0  0
    5.5330   -2.0172   -0.1335 H   0  0  0  0  0  0  0  0  0  0  0  0
    5.9249   -0.3019   -0.0621 H   0  0  0  0  0  0  0  0  0  0  0  0
    2.3707   -4.6925   -2.0398 H   0  0  0  0  0  0  0  0  0  0  0  0
    1.4942   -5.1232   -0.6439 H   0  0  0  0  0  0  0  0  0  0  0  0
    2.3643   -4.7627   -4.0740 H   0  0  0  0  0  0  0  0  0  0  0  0
    2.2237   -4.1263   -6.4275 H   0  0  0  0  0  0  0  0  0  0  0  0
    0.1711   -3.0677   -7.3143 H   0  0  0  0  0  0  0  0  0  0  0  0
   -1.7838   -2.7344   -5.8193 H   0  0  0  0  0  0  0  0  0  0  0  0
    4.7459    2.3833   -4.1069 H   0  0  0  0  0  0  0  0  0  0  0  0
    4.1113    2.2462   -6.4617 H   0  0  0  0  0  0  0  0  0  0  0  0
    3.0402    0.2009   -7.3490 H   0  0  0  0  0  0  0  0  0  0  0  0
    2.6951   -1.7529   -5.8546 H   0  0  0  0  0  0  0  0  0  0  0  0
    4.6737    2.3906   -2.0702 H   0  0  0  0  0  0  0  0  0  0  0  0
    5.0946    1.5102   -0.6748 H   0  0  0  0  0  0  0  0  0  0  0  0
    2.0208    5.5647   -0.0880 H   0  0  0  0  0  0  0  0  0  0  0  0
    0.3043    5.9505   -0.0142 H   0  0  0  0  0  0  0  0  0  0  0  0
   -2.3825    4.7050   -2.0356 H   0  0  0  0  0  0  0  0  0  0  0  0
   -1.5052    5.1240   -0.6375 H   0  0  0  0  0  0  0  0  0  0  0  0
   -2.3763    4.7839   -4.0699 H   0  0  0  0  0  0  0  0  0  0  0  0
   -2.2372    4.1587   -6.4267 H   0  0  0  0  0  0  0  0  0  0  0  0
   -0.1863    3.1018   -7.3191 H   0  0  0  0  0  0  0  0  0  0  0  0
    1.7686    2.7587   -5.8263 H   0  0  0  0  0  0  0  0  0  0  0  0
   -4.6796   -2.3770   -2.0638 H   0  0  0  0  0  0  0  0  0  0  0  0
   -5.1008   -1.5038   -0.6636 H   0  0  0  0  0  0  0  0  0  0  0  0
   -4.7525   -2.3594   -4.1002 H   0  0  0  0  0  0  0  0  0  0  0  0
   -4.1214   -2.2091   -6.4553 H   0  0  0  0  0  0  0  0  0  0  0  0
   -3.0554   -0.1574   -7.3335 H   0  0  0  0  0  0  0  0  0  0  0  0
   -2.7122    1.7895   -5.8299 H   0  0  0  0  0  0  0  0  0  0  0  0
    2.6415   -1.7667    7.1922 H   0  0  0  0  0  0  0  0  0  0  0  0
    2.6982   -3.5171    7.4134 H   0  0  0  0  0  0  0  0  0  0  0  0
    4.1891   -2.5743    7.4345 H   0  0  0  0  0  0  0  0  0  0  0  0
    3.6435   -5.0064    5.4724 H   0  0  0  0  0  0  0  0  0  0  0  0
    4.4299   -4.1884    4.1169 H   0  0  0  0  0  0  0  0  0  0  0  0
    5.1186   -4.0724    5.7286 H   0  0  0  0  0  0  0  0  0  0  0  0
   -2.6907   -2.6996    7.4703 H   0  0  0  0  0  0  0  0  0  0  0  0
   -4.3536   -2.1715    7.1955 H   0  0  0  0  0  0  0  0  0  0  0  0
   -4.0072   -3.8722    7.5079 H   0  0  0  0  0  0  0  0  0  0  0  0
   -2.4140   -4.6148    4.2314 H   0  0  0  0  0  0  0  0  0  0  0  0
   -1.5084   -4.1264    5.6607 H   0  0  0  0  0  0  0  0  0  0  0  0
   -2.8478   -5.2666    5.8109 H   0  0  0  0  0  0  0  0  0  0  0  0
   -2.6582    2.6662    7.4801 H   0  0  0  0  0  0  0  0  0  0  0  0
   -2.1472    4.3329    7.1965 H   0  0  0  0  0  0  0  0  0  0  0  0
   -3.8425    3.9722    7.5210 H   0  0  0  0  0  0  0  0  0  0  0  0
   -4.5893    2.3586    4.2541 H   0  0  0  0  0  0  0  0  0  0  0  0
   -4.0987    1.4727    5.6959 H   0  0  0  0  0  0  0  0  0  0  0  0
   -5.2432    2.8096    5.8282 H   0  0  0  0  0  0  0  0  0  0  0  0
    2.6845    2.6712    7.4702 H   0  0  0  0  0  0  0  0  0  0  0  0
    4.3463    2.1431    7.1916 H   0  0  0  0  0  0  0  0  0  0  0  0
    4.0017    3.8429    7.5125 H   0  0  0  0  0  0  0  0  0  0  0  0
    2.3975    4.5955    4.2406 H   0  0  0  0  0  0  0  0  0  0  0  0
    1.5030    4.1076    5.6778 H   0  0  0  0  0  0  0  0  0  0  0  0
    2.8433    5.2475    5.8169 H   0  0  0  0  0  0  0  0  0  0  0  0
  1  6  1  0  0  0  0
  1 35  1  0  0  0  0
  2  1  1  0  0  0  0
  3  4  1  0  0  0  0
  3  2  1  0  0  0  0
  4  5  1  0  0  0  0
  5  6  1  0  0  0  0
  5  9  1  0  0  0  0
  6105  1  0  0  0  0
  7  2  1  0  0  0  0
  8107  1  0  0  0  0
  8  4  1  0  0  0  0
  9108  1  0  0  0  0
  9 36  1  0  0  0  0
 10 15  1  0  0  0  0
 10  9  1  0  0  0  0
```

11 10 1 0 0 0 0
12 13 1 0 0 0 0
12 11 1 0 0 0 0
13 14 1 0 0 0 0
14 15 1 0 0 0 0
14 17 1 0 0 0 0
15109 1 0 0 0 0
16 11 1 0 0 0 0
17111 1 0 0 0 0
17 37 1 0 0 0 0
18 23 1 0 0 0 0
18 17 1 0 0 0 0
19 18 1 0 0 0 0
20 21 1 0 0 0 0
20 19 1 0 0 0 0
21 22 1 0 0 0 0
22 23 1 0 0 0 0
22 26 1 0 0 0 0
23112 1 0 0 0 0
24 19 1 0 0 0 0
25114 1 0 0 0 0
25 21 1 0 0 0 0
26115 1 0 0 0 0
26 38 1 0 0 0 0
27 32 1 0 0 0 0
27 26 1 0 0 0 0
28 27 1 0 0 0 0
29 30 1 0 0 0 0
29 28 1 0 0 0 0
30 31 1 0 0 0 0
31 32 1 0 0 0 0
31 35 1 0 0 0 0
32116 1 0 0 0 0
33 28 1 0 0 0 0
34118 1 0 0 0 0
34 30 1 0 0 0 0
35119 1 0 0 0 0
35 39 1 0 0 0 0
36120 1 0 0 0 0
36121 1 0 0 0 0
36 40 1 0 0 0 0
37122 1 0 0 0 0
37123 1 0 0 0 0
37 41 1 0 0 0 0
38124 1 0 0 0 0
38125 1 0 0 0 0
38 42 1 0 0 0 0
39126 1 0 0 0 0
39127 1 0 0 0 0
39 43 1 0 0 0 0
40 97 1 0 0 0 0
41103 1 0 0 0 0
42101 1 0 0 0 0
43 99 1 0 0 0 0
44133 1 0 0 0 0
44132 1 0 0 0 0
44 29 1 0 0 0 0
45134 1 0 0 0 0
45 13 1 0 0 0 0
46136 1 0 0 0 0
46135 1 0 0 0 0
46 3 1 0 0 0 0
47138 1 0 0 0 0
47137 1 0 0 0 0
47 12 1 0 0 0 0
48 46 1 0 0 0 0
49 55 1 0 0 0 0
49 48 1 0 0 0 0
50 49 1 0 0 0 0
51 50 1 0 0 0 0
51 52 1 0 0 0 0
52 53 1 0 0 0 0
53 48 1 0 0 0 0
53 54 1 0 0 0 0
55140 1 0 0 0 0
56141 1 0 0 0 0
56 50 1 0 0 0 0

57 56 1 0 0 0 0
58 57 1 0 0 0 0
58 59 1 0 0 0 0
59 51 1 0 0 0 0
60 47 1 0 0 0 0
61 71 1 0 0 0 0
61 60 1 0 0 0 0
62 61 1 0 0 0 0
63 62 1 0 0 0 0
63 64 1 0 0 0 0
64 65 1 0 0 0 0
65 60 1 0 0 0 0
65 66 1 0 0 0 0
67145 1 0 0 0 0
67 62 1 0 0 0 0
68 67 1 0 0 0 0
69 68 1 0 0 0 0
69 70 1 0 0 0 0
70 63 1 0 0 0 0
71150 1 0 0 0 0
72152 1 0 0 0 0
72151 1 0 0 0 0
72 20 1 0 0 0 0
73 72 1 0 0 0 0
74 79 1 0 0 0 0
74 75 1 0 0 0 0
75 76 1 0 0 0 0
76 73 1 0 0 0 0
76 77 1 0 0 0 0
78 80 1 0 0 0 0
78 73 1 0 0 0 0
79 78 1 0 0 0 0
80154 1 0 0 0 0
81155 1 0 0 0 0
81 79 1 0 0 0 0
82 81 1 0 0 0 0
83 82 1 0 0 0 0
83 84 1 0 0 0 0
84 74 1 0 0 0 0
85 44 1 0 0 0 0
86 91 1 0 0 0 0
86 87 1 0 0 0 0
87 88 1 0 0 0 0
88 85 1 0 0 0 0
88 89 1 0 0 0 0
90 92 1 0 0 0 0
90 85 1 0 0 0 0
91 90 1 0 0 0 0
92160 1 0 0 0 0
93161 1 0 0 0 0
93 91 1 0 0 0 0
94 93 1 0 0 0 0
95 94 1 0 0 0 0
95 96 1 0 0 0 0
96 86 1 0 0 0 0
97165 1 0 0 0 0
97166 1 0 0 0 0
97167 1 0 0 0 0
98 40 1 0 0 0 0
98168 1 0 0 0 0
98170 1 0 0 0 0
99172 1 0 0 0 0
99171 1 0 0 0 0
99173 1 0 0 0 0
100 43 1 0 0 0 0
100175 1 0 0 0 0
100176 1 0 0 0 0
101178 1 0 0 0 0
101177 1 0 0 0 0
101179 1 0 0 0 0
102 42 1 0 0 0 0
102181 1 0 0 0 0
102182 1 0 0 0 0
103184 1 0 0 0 0
103183 1 0 0 0 0
103185 1 0 0 0 0
104 41 1 0 0 0 0

```
104187  1  0  0  0  0
104188  1  0  0  0  0
106  7  1  0  0  0  0
110 16  1  0  0  0  0
113 24  1  0  0  0  0
117 33  1  0  0  0  0
128 40  1  0  0  0  0
129 41  1  0  0  0  0
130 42  1  0  0  0  0
131 43  1  0  0  0  0
139 55  1  0  0  0  0
142 57  1  0  0  0  0
143 58  1  0  0  0  0
144 59  1  0  0  0  0
146 68  1  0  0  0  0
147 69  1  0  0  0  0
148 70  1  0  0  0  0
149 71  1  0  0  0  0
153 80  1  0  0  0  0
156 82  1  0  0  0  0
157 83  1  0  0  0  0
158 84  1  0  0  0  0
159 92  1  0  0  0  0
162 94  1  0  0  0  0
163 95  1  0  0  0  0
164 96  1  0  0  0  0
169 98  1  0  0  0  0
174100  1  0  0  0  0
180102  1  0  0  0  0
186104  1  0  0  0  0
M  END
```

Crown-*in*-DMSO

E = -299.912566214561 Eh

```
188200  0     0  0            999 V2000
   -1.7701    -2.6798    2.6947 C   0  0  0  0  0  0  0  0  0  0  0  0
   -1.9352    -3.5156    1.5828 C   0  0  0  0  0  0  0  0  0  0  0  0
   -0.8451    -4.1879    1.0292 C   0  0  0  0  0  0  0  0  0  0  0  0
    0.4160    -4.0269    1.6120 C   0  0  0  0  0  0  0  0  0  0  0  0
    0.6113    -3.1553    2.6858 C   0  0  0  0  0  0  0  0  0  0  0  0
   -0.4914    -2.4798    3.1803 C   0  0  0  0  0  0  0  0  0  0  0  0
   -3.2033    -3.6760    1.0899 O   0  0  0  0  0  0  0  0  0  0  0  0
    1.4405    -4.7854    1.1106 O   0  0  0  0  0  0  0  0  0  0  0  0
    1.9922    -2.9688    3.2894 C   0  0  0  0  0  0  0  0  0  0  0  0
    2.6609    -1.7562    2.6711 C   0  0  0  0  0  0  0  0  0  0  0  0
    3.4855    -1.9187    1.5512 C   0  0  0  0  0  0  0  0  0  0  0  0
    4.1601    -0.8297    0.9984 C   0  0  0  0  0  0  0  0  0  0  0  0
    4.0088     0.4295    1.5882 C   0  0  0  0  0  0  0  0  0  0  0  0
    3.1546     0.6200    2.6759 C   0  0  0  0  0  0  0  0  0  0  0  0
    2.4841    -0.4824    3.1786 C   0  0  0  0  0  0  0  0  0  0  0  0
    3.6345    -3.1847    1.0498 O   0  0  0  0  0  0  0  0  0  0  0  0
    2.9825     1.9911    3.3053 C   0  0  0  0  0  0  0  0  0  0  0  0
    1.7661     2.6623    2.6943 C   0  0  0  0  0  0  0  0  0  0  0  0
    1.9280     3.5009    1.5834 C   0  0  0  0  0  0  0  0  0  0  0  0
    0.8377     4.1746    1.0323 C   0  0  0  0  0  0  0  0  0  0  0  0
   -0.4231     4.0115    1.6153 C   0  0  0  0  0  0  0  0  0  0  0  0
   -0.6148     3.1383    2.6870 C   0  0  0  0  0  0  0  0  0  0  0  0
    0.4880     2.4626    3.1823 C   0  0  0  0  0  0  0  0  0  0  0  0
    3.1955     3.6662    1.0899 O   0  0  0  0  0  0  0  0  0  0  0  0
   -1.4481     4.7730    1.1202 O   0  0  0  0  0  0  0  0  0  0  0  0
   -1.9868     2.9584    3.3131 C   0  0  0  0  0  0  0  0  0  0  0  0
   -2.6598     1.7460    2.6967 C   0  0  0  0  0  0  0  0  0  0  0  0
   -3.4898     1.9138    1.5801 C   0  0  0  0  0  0  0  0  0  0  0  0
   -4.1653     0.8280    1.0223 C   0  0  0  0  0  0  0  0  0  0  0  0
   -4.0124    -0.4348    1.6037 C   0  0  0  0  0  0  0  0  0  0  0  0
   -3.1512    -0.6315    2.6840 C   0  0  0  0  0  0  0  0  0  0  0  0
   -2.4734     0.4667    3.1863 C   0  0  0  0  0  0  0  0  0  0  0  0
   -3.6458     3.1828    1.0880 O   0  0  0  0  0  0  0  0  0  0  0  0
   -4.7721    -1.4563    1.0987 O   0  0  0  0  0  0  0  0  0  0  0  0
   -2.9829    -2.0056    3.3080 C   0  0  0  0  0  0  0  0  0  0  0  0
    1.9589    -2.9334    4.8235 C   0  0  0  0  0  0  0  0  0  0  0  0
    2.9745     1.9152    4.8414 C   0  0  0  0  0  0  0  0  0  0  0  0
   -1.9100     2.9460    4.8491 C   0  0  0  0  0  0  0  0  0  0  0  0
```

-2.9761	-1.9339	4.8442	C	0	0	0	0	0	0	0	0	0	0	0	0
3.3506	-2.8602	5.4637	C	0	0	0	0	0	0	0	0	0	0	0	0
3.4648	3.1996	5.5201	C	0	0	0	0	0	0	0	0	0	0	0	0
-3.1954	3.4295	5.5307	C	0	0	0	0	0	0	0	0	0	0	0	0
-3.4596	-3.2223	5.5201	C	0	0	0	0	0	0	0	0	0	0	0	0
-5.1240	1.0098	-0.1396	C	0	0	0	0	0	0	0	0	0	0	0	0
4.7662	1.4539	1.0857	O	0	0	0	0	0	0	0	0	0	0	0	0
-1.0204	-5.1461	-0.1341	C	0	0	0	0	0	0	0	0	0	0	0	0
5.1152	-1.0057	-0.1673	C	0	0	0	0	0	0	0	0	0	0	0	0
-0.8690	-4.5413	-1.5179	C	0	0	0	0	0	0	0	0	0	0	0	0
0.3432	-4.5229	-2.1995	C	0	0	0	0	0	0	0	0	0	0	0	0
0.3356	-4.1404	-3.6017	C	0	0	0	0	0	0	0	0	0	0	0	0
-0.8577	-3.6544	-4.1476	C	0	0	0	0	0	0	0	0	0	0	0	0
-1.9812	-3.5310	-3.4039	O	0	0	0	0	0	0	0	0	0	0	0	0
-2.0360	-4.0137	-2.1252	C	0	0	0	0	0	0	0	0	0	0	0	0
-3.1467	-3.9184	-1.5868	O	0	0	0	0	0	0	0	0	0	0	0	0
1.4873	-4.9286	-1.6386	N	0	0	0	0	0	0	0	0	0	0	0	0
1.4365	-4.2970	-4.4523	C	0	0	0	0	0	0	0	0	0	0	0	0
1.3451	-3.9753	-5.7877	C	0	0	0	0	0	0	0	0	0	0	0	0
0.1619	-3.4579	-6.3075	C	0	0	0	0	0	0	0	0	0	0	0	0
-0.9355	-3.2929	-5.4920	C	0	0	0	0	0	0	0	0	0	0	0	0
4.5118	-0.8484	-1.5508	C	0	0	0	0	0	0	0	0	0	0	0	0
4.5010	0.3658	-2.2292	C	0	0	0	0	0	0	0	0	0	0	0	0
4.1227	0.3633	-3.6325	C	0	0	0	0	0	0	0	0	0	0	0	0
3.6313	-0.8262	-4.1821	C	0	0	0	0	0	0	0	0	0	0	0	0
3.5001	-1.9506	-3.4414	O	0	0	0	0	0	0	0	0	0	0	0	0
3.9802	-2.0112	-2.1622	C	0	0	0	0	0	0	0	0	0	0	0	0
3.8799	-3.1231	-1.6268	O	0	0	0	0	0	0	0	0	0	0	0	0
4.2886	1.4648	-4.4805	C	0	0	0	0	0	0	0	0	0	0	0	0
3.9702	1.3779	-5.8171	C	0	0	0	0	0	0	0	0	0	0	0	0
3.4466	0.1990	-6.3405	C	0	0	0	0	0	0	0	0	0	0	0	0
3.2725	-0.8989	-5.5276	C	0	0	0	0	0	0	0	0	0	0	0	0
4.9106	1.5059	-1.6633	N	0	0	0	0	0	0	0	0	0	0	0	0
1.0117	5.1401	-0.1251	C	0	0	0	0	0	0	0	0	0	0	0	0
0.8578	4.5441	-1.5123	C	0	0	0	0	0	0	0	0	0	0	0	0
0.8424	3.6726	-4.1468	C	0	0	0	0	0	0	0	0	0	0	0	0
1.9667	3.5439	-3.4054	O	0	0	0	0	0	0	0	0	0	0	0	0
2.0235	4.0192	-2.1240	C	0	0	0	0	0	0	0	0	0	0	0	0
3.1347	3.9205	-1.5873	O	0	0	0	0	0	0	0	0	0	0	0	0
-0.3554	4.5311	-2.1923	C	0	0	0	0	0	0	0	0	0	0	0	0
-0.3498	4.1567	-3.5967	C	0	0	0	0	0	0	0	0	0	0	0	0
-1.4981	4.9350	-1.6274	N	0	0	0	0	0	0	0	0	0	0	0	0
-1.4516	4.3192	-4.4450	C	0	0	0	0	0	0	0	0	0	0	0	0
-1.3622	4.0048	-5.7824	C	0	0	0	0	0	0	0	0	0	0	0	0
-0.1802	3.4891	-6.3065	C	0	0	0	0	0	0	0	0	0	0	0	0
0.9181	3.3186	-5.4933	C	0	0	0	0	0	0	0	0	0	0	0	0
-4.5235	0.8600	-1.5252	C	0	0	0	0	0	0	0	0	0	0	0	0
-3.6476	0.8536	-4.1581	C	0	0	0	0	0	0	0	0	0	0	0	0
-3.5166	1.9741	-3.4114	O	0	0	0	0	0	0	0	0	0	0	0	0
-3.9944	2.0270	-2.1310	C	0	0	0	0	0	0	0	0	0	0	0	0
-3.8945	3.1357	-1.5894	O	0	0	0	0	0	0	0	0	0	0	0	0
-4.5122	-0.3504	-2.2102	C	0	0	0	0	0	0	0	0	0	0	0	0
-4.1361	-0.3397	-3.6141	C	0	0	0	0	0	0	0	0	0	0	0	0
-4.9194	-1.4942	-1.6500	N	0	0	0	0	0	0	0	0	0	0	0	0
-4.3016	-1.4369	-4.4678	C	0	0	0	0	0	0	0	0	0	0	0	0
-3.9857	-1.3421	-5.8045	C	0	0	0	0	0	0	0	0	0	0	0	0
-3.4652	-0.1594	-6.3225	C	0	0	0	0	0	0	0	0	0	0	0	0
-3.2916	0.9344	-5.5038	C	0	0	0	0	0	0	0	0	0	0	0	0
3.2091	-2.6394	6.9704	C	0	0	0	0	0	0	0	0	0	0	0	0
4.1744	-4.1199	5.2009	C	0	0	0	0	0	0	0	0	0	0	0	0
-3.6147	-2.9875	7.0235	C	0	0	0	0	0	0	0	0	0	0	0	0
-2.5119	-4.3928	5.2704	C	0	0	0	0	0	0	0	0	0	0	0	0
-2.9560	3.5785	7.0341	C	0	0	0	0	0	0	0	0	0	0	0	0
-4.3691	2.4859	5.2810	C	0	0	0	0	0	0	0	0	0	0	0	0
3.6160	2.9613	7.0233	C	0	0	0	0	0	0	0	0	0	0	0	0
2.5245	4.3761	5.2707	C	0	0	0	0	0	0	0	0	0	0	0	0
-0.3488	-1.7881	3.9921	H	0	0	0	0	0	0	0	0	0	0	0	0
-3.1857	-3.9286	0.1364	H	0	0	0	0	0	0	0	0	0	0	0	0
2.2980	-4.3336	1.2596	H	0	0	0	0	0	0	0	0	0	0	0	0
2.5968	-3.8408	3.0319	H	0	0	0	0	0	0	0	0	0	0	0	0
1.8229	-0.3415	4.0158	H	0	0	0	0	0	0	0	0	0	0	0	0
3.8841	-3.1635	0.0956	H	0	0	0	0	0	0	0	0	0	0	0	0
3.8541	2.6013	3.0511	H	0	0	0	0	0	0	0	0	0	0	0	0
0.3405	1.7771	3.9990	H	0	0	0	0	0	0	0	0	0	0	0	0
3.1761	3.9218	0.1372	H	0	0	0	0	0	0	0	0	0	0	0	0
-2.3067	4.3266	1.2796	H	0	0	0	0	0	0	0	0	0	0	0	0
-2.5972	3.8312	3.0636	H	0	0	0	0	0	0	0	0	0	0	0	0

```
 -1.7977    0.3147    4.0102 H   0  0  0  0  0  0  0  0  0  0  0  0
 -3.8977    3.1670    0.1343 H   0  0  0  0  0  0  0  0  0  0  0  0
 -4.3337   -2.3177    1.2650 H   0  0  0  0  0  0  0  0  0  0  0  0
 -3.8573   -2.6113    3.0529 H   0  0  0  0  0  0  0  0  0  0  0  0
  1.4507   -3.8372    5.1704 H   0  0  0  0  0  0  0  0  0  0  0  0
  1.3813   -2.0804    5.1769 H   0  0  0  0  0  0  0  0  0  0  0  0
  3.6419    1.1014    5.1362 H   0  0  0  0  0  0  0  0  0  0  0  0
  1.9816    1.6820    5.2223 H   0  0  0  0  0  0  0  0  0  0  0  0
 -1.0994    3.6180    5.1431 H   0  0  0  0  0  0  0  0  0  0  0  0
 -1.6703    1.9539    5.2283 H   0  0  0  0  0  0  0  0  0  0  0  0
 -3.6500   -1.1250    5.1384 H   0  0  0  0  0  0  0  0  0  0  0  0
 -1.9855   -1.6948    5.2276 H   0  0  0  0  0  0  0  0  0  0  0  0
  3.8812   -2.0030    5.0344 H   0  0  0  0  0  0  0  0  0  0  0  0
  4.4515    3.4527    5.1136 H   0  0  0  0  0  0  0  0  0  0  0  0
 -3.4523    4.4163    5.1268 H   0  0  0  0  0  0  0  0  0  0  0  0
 -4.4444   -3.4801    5.1117 H   0  0  0  0  0  0  0  0  0  0  0  0
 -5.5655    2.0082   -0.0803 H   0  0  0  0  0  0  0  0  0  0  0  0
 -5.9327    0.2872   -0.0283 H   0  0  0  0  0  0  0  0  0  0  0  0
  4.3273    2.3141    1.2573 H   0  0  0  0  0  0  0  0  0  0  0  0
 -2.0175   -5.5910   -0.0783 H   0  0  0  0  0  0  0  0  0  0  0  0
 -0.2955   -5.9528   -0.0226 H   0  0  0  0  0  0  0  0  0  0  0  0
  5.5558   -2.0048   -0.1140 H   0  0  0  0  0  0  0  0  0  0  0  0
  5.9250   -0.2845   -0.0545 H   0  0  0  0  0  0  0  0  0  0  0  0
  2.3658   -4.6329   -2.0374 H   0  0  0  0  0  0  0  0  0  0  0  0
  1.4932   -5.0521   -0.6288 H   0  0  0  0  0  0  0  0  0  0  0  0
  2.3628   -4.6889   -4.0645 H   0  0  0  0  0  0  0  0  0  0  0  0
  2.1943   -4.1323   -6.4356 H   0  0  0  0  0  0  0  0  0  0  0  0
  0.0985   -3.1941   -7.3516 H   0  0  0  0  0  0  0  0  0  0  0  0
 -1.8673   -2.9010   -5.8651 H   0  0  0  0  0  0  0  0  0  0  0  0
  4.6855    2.3878   -4.0898 H   0  0  0  0  0  0  0  0  0  0  0  0
  4.1345    2.2271   -6.4632 H   0  0  0  0  0  0  0  0  0  0  0  0
  3.1850    0.1396   -7.3853 H   0  0  0  0  0  0  0  0  0  0  0  0
  2.8759   -1.8276   -5.9034 H   0  0  0  0  0  0  0  0  0  0  0  0
  4.6244    2.3879   -2.0610 H   0  0  0  0  0  0  0  0  0  0  0  0
  5.0288    1.5092   -0.6528 H   0  0  0  0  0  0  0  0  0  0  0  0
  2.0090    5.5844   -0.0682 H   0  0  0  0  0  0  0  0  0  0  0  0
  0.2872    5.9461   -0.0072 H   0  0  0  0  0  0  0  0  0  0  0  0
 -2.3778    4.6437   -2.0267 H   0  0  0  0  0  0  0  0  0  0  0  0
 -1.5030    5.0509   -0.6166 H   0  0  0  0  0  0  0  0  0  0  0  0
 -2.3770    4.7101   -4.0539 H   0  0  0  0  0  0  0  0  0  0  0  0
 -2.2121    4.1661   -6.4284 H   0  0  0  0  0  0  0  0  0  0  0  0
 -0.1185    3.2312   -7.3521 H   0  0  0  0  0  0  0  0  0  0  0  0
  1.8490    2.9280   -5.8697 H   0  0  0  0  0  0  0  0  0  0  0  0
 -4.6342   -2.3739   -2.0534 H   0  0  0  0  0  0  0  0  0  0  0  0
 -5.0370   -1.5033   -0.6394 H   0  0  0  0  0  0  0  0  0  0  0  0
 -4.6961   -2.3627   -4.0815 H   0  0  0  0  0  0  0  0  0  0  0  0
 -4.1496   -2.1881   -6.4549 H   0  0  0  0  0  0  0  0  0  0  0  0
 -3.2060   -0.0937   -7.3674 H   0  0  0  0  0  0  0  0  0  0  0  0
 -2.8975    1.8658   -5.8753 H   0  0  0  0  0  0  0  0  0  0  0  0
  2.6495   -1.7300    7.1782 H   0  0  0  0  0  0  0  0  0  0  0  0
  2.6853   -3.4768    7.4276 H   0  0  0  0  0  0  0  0  0  0  0  0
  4.1872   -2.5513    7.4381 H   0  0  0  0  0  0  0  0  0  0  0  0
  3.6289   -5.0055    5.5218 H   0  0  0  0  0  0  0  0  0  0  0  0
  4.4139   -4.2206    4.1462 H   0  0  0  0  0  0  0  0  0  0  0  0
  5.1111   -4.0771    5.7527 H   0  0  0  0  0  0  0  0  0  0  0  0
 -2.6552   -2.7287    7.4671 H   0  0  0  0  0  0  0  0  0  0  0  0
 -4.3140   -2.1770    7.2181 H   0  0  0  0  0  0  0  0  0  0  0  0
 -3.9861   -3.8845    7.5137 H   0  0  0  0  0  0  0  0  0  0  0  0
 -2.4429   -4.6180    4.2106 H   0  0  0  0  0  0  0  0  0  0  0  0
 -1.5143   -4.1555    5.6331 H   0  0  0  0  0  0  0  0  0  0  0  0
 -2.8653   -5.2812    5.7898 H   0  0  0  0  0  0  0  0  0  0  0  0
 -2.6974    2.6170    7.4733 H   0  0  0  0  0  0  0  0  0  0  0  0
 -2.1440    4.2759    7.2288 H   0  0  0  0  0  0  0  0  0  0  0  0
 -3.8511    3.9494    7.5283 H   0  0  0  0  0  0  0  0  0  0  0  0
 -4.5938    2.4160    4.2211 H   0  0  0  0  0  0  0  0  0  0  0  0
 -4.1358    1.4880    5.6459 H   0  0  0  0  0  0  0  0  0  0  0  0
 -5.2568    2.8427    5.7995 H   0  0  0  0  0  0  0  0  0  0  0  0
  2.6545    2.7066    7.4646 H   0  0  0  0  0  0  0  0  0  0  0  0
  4.3104    2.1467    7.2173 H   0  0  0  0  0  0  0  0  0  0  0  0
  3.9912    3.8556    7.5158 H   0  0  0  0  0  0  0  0  0  0  0  0
  2.4532    4.5996    4.2107 H   0  0  0  0  0  0  0  0  0  0  0  0
  1.5266    4.1465    5.6378 H   0  0  0  0  0  0  0  0  0  0  0  0
  2.8852    5.2634    5.7872 H   0  0  0  0  0  0  0  0  0  0  0  0
  1  6  1  0  0  0  0
  1 35  1  0  0  0  0
  2  1  1  0  0  0  0
  3  4  1  0  0  0  0
```

3 2 1 0 0 0 0
4 5 1 0 0 0 0
5 6 1 0 0 0 0
5 9 1 0 0 0 0
6105 1 0 0 0 0
7 2 1 0 0 0 0
8107 1 0 0 0 0
8 4 1 0 0 0 0
9108 1 0 0 0 0
9 36 1 0 0 0 0
10 15 1 0 0 0 0
10 9 1 0 0 0 0
11 10 1 0 0 0 0
12 13 1 0 0 0 0
12 11 1 0 0 0 0
13 14 1 0 0 0 0
14 15 1 0 0 0 0
14 17 1 0 0 0 0
15109 1 0 0 0 0
16 11 1 0 0 0 0
17111 1 0 0 0 0
17 37 1 0 0 0 0
18 23 1 0 0 0 0
18 17 1 0 0 0 0
19 18 1 0 0 0 0
20 21 1 0 0 0 0
20 19 1 0 0 0 0
21 22 1 0 0 0 0
22 23 1 0 0 0 0
22 26 1 0 0 0 0
23112 1 0 0 0 0
24 19 1 0 0 0 0
25114 1 0 0 0 0
25 21 1 0 0 0 0
26115 1 0 0 0 0
26 38 1 0 0 0 0
27 32 1 0 0 0 0
27 26 1 0 0 0 0
28 27 1 0 0 0 0
29 30 1 0 0 0 0
29 28 1 0 0 0 0
30 31 1 0 0 0 0
31 32 1 0 0 0 0
31 35 1 0 0 0 0
32116 1 0 0 0 0
33 28 1 0 0 0 0
34118 1 0 0 0 0
34 30 1 0 0 0 0
35119 1 0 0 0 0
35 39 1 0 0 0 0
36120 1 0 0 0 0
36121 1 0 0 0 0
36 40 1 0 0 0 0
37122 1 0 0 0 0
37123 1 0 0 0 0
37 41 1 0 0 0 0
38124 1 0 0 0 0
38125 1 0 0 0 0
38 42 1 0 0 0 0
39126 1 0 0 0 0
39127 1 0 0 0 0
39 43 1 0 0 0 0
40 97 1 0 0 0 0
41103 1 0 0 0 0
42101 1 0 0 0 0
43 99 1 0 0 0 0
44133 1 0 0 0 0
44132 1 0 0 0 0
44 29 1 0 0 0 0
45134 1 0 0 0 0
45 13 1 0 0 0 0
46136 1 0 0 0 0
46135 1 0 0 0 0
46 3 1 0 0 0 0
47138 1 0 0 0 0
47137 1 0 0 0 0
47 12 1 0 0 0 0

48 46 1 0 0 0 0
49 55 1 0 0 0 0
49 48 1 0 0 0 0
50 49 1 0 0 0 0
51 50 1 0 0 0 0
51 52 1 0 0 0 0
52 53 1 0 0 0 0
53 48 1 0 0 0 0
53 54 1 0 0 0 0
55140 1 0 0 0 0
56141 1 0 0 0 0
56 50 1 0 0 0 0
57 56 1 0 0 0 0
58 57 1 0 0 0 0
58 59 1 0 0 0 0
59 51 1 0 0 0 0
60 47 1 0 0 0 0
61 71 1 0 0 0 0
61 60 1 0 0 0 0
62 61 1 0 0 0 0
63 62 1 0 0 0 0
63 64 1 0 0 0 0
64 65 1 0 0 0 0
65 60 1 0 0 0 0
65 66 1 0 0 0 0
67145 1 0 0 0 0
67 62 1 0 0 0 0
68 67 1 0 0 0 0
69 68 1 0 0 0 0
69 70 1 0 0 0 0
70 63 1 0 0 0 0
71150 1 0 0 0 0
72152 1 0 0 0 0
72151 1 0 0 0 0
72 20 1 0 0 0 0
73 72 1 0 0 0 0
74 79 1 0 0 0 0
74 75 1 0 0 0 0
75 76 1 0 0 0 0
76 73 1 0 0 0 0
76 77 1 0 0 0 0
78 80 1 0 0 0 0
78 73 1 0 0 0 0
79 78 1 0 0 0 0
80154 1 0 0 0 0
81155 1 0 0 0 0
81 79 1 0 0 0 0
82 81 1 0 0 0 0
83 82 1 0 0 0 0
83 84 1 0 0 0 0
84 74 1 0 0 0 0
85 44 1 0 0 0 0
86 91 1 0 0 0 0
86 87 1 0 0 0 0
87 88 1 0 0 0 0
88 85 1 0 0 0 0
88 89 1 0 0 0 0
90 92 1 0 0 0 0
90 85 1 0 0 0 0
91 90 1 0 0 0 0
92160 1 0 0 0 0
93161 1 0 0 0 0
93 91 1 0 0 0 0
94 93 1 0 0 0 0
95 94 1 0 0 0 0
95 96 1 0 0 0 0
96 86 1 0 0 0 0
97165 1 0 0 0 0
97166 1 0 0 0 0
97167 1 0 0 0 0
98 40 1 0 0 0 0
98168 1 0 0 0 0
98170 1 0 0 0 0
99172 1 0 0 0 0
99171 1 0 0 0 0
99173 1 0 0 0 0
100 43 1 0 0 0 0

```
100175  1  0  0  0  0
100176  1  0  0  0  0
101178  1  0  0  0  0
101177  1  0  0  0  0
101179  1  0  0  0  0
102 42  1  0  0  0  0
102181  1  0  0  0  0
102182  1  0  0  0  0
103184  1  0  0  0  0
103183  1  0  0  0  0
103185  1  0  0  0  0
104 41  1  0  0  0  0
104187  1  0  0  0  0
104188  1  0  0  0  0
106  7  1  0  0  0  0
110 16  1  0  0  0  0
113 24  1  0  0  0  0
117 33  1  0  0  0  0
128 40  1  0  0  0  0
129 41  1  0  0  0  0
130 42  1  0  0  0  0
131 43  1  0  0  0  0
139 55  1  0  0  0  0
142 57  1  0  0  0  0
143 58  1  0  0  0  0
144 59  1  0  0  0  0
146 68  1  0  0  0  0
147 69  1  0  0  0  0
148 70  1  0  0  0  0
149 71  1  0  0  0  0
153 80  1  0  0  0  0
156 82  1  0  0  0  0
157 83  1  0  0  0  0
158 84  1  0  0  0  0
159 92  1  0  0  0  0
162 94  1  0  0  0  0
163 95  1  0  0  0  0
164 96  1  0  0  0  0
169 98  1  0  0  0  0
174100  1  0  0  0  0
180102  1  0  0  0  0
186104  1  0  0  0  0
M  END
```

Crown-*out*-gas phase

E = -299.794639680918 Eh

```
188200  0     0  0            999 V2000
    0.9101    3.1018   -0.8515 C   0  0  0  0  0  0  0  0  0  0  0  0
    1.5005    3.7858    0.2137 C   0  0  0  0  0  0  0  0  0  0  0  0
    2.7653    3.4304    0.6914 C   0  0  0  0  0  0  0  0  0  0  0  0
    3.4233    2.3422    0.1207 C   0  0  0  0  0  0  0  0  0  0  0  0
    2.8120    1.5969   -0.8950 C   0  0  0  0  0  0  0  0  0  0  0  0
    1.5643    1.9842   -1.3433 C   0  0  0  0  0  0  0  0  0  0  0  0
    0.8787    4.8386    0.8313 O   0  0  0  0  0  0  0  0  0  0  0  0
    4.6486    1.9183    0.5420 O   0  0  0  0  0  0  0  0  0  0  0  0
    1.0899    1.3998   -2.1125 H   0  0  0  0  0  0  0  0  0  0  0  0
    3.5449    0.4005   -1.4685 C   0  0  0  0  0  0  0  0  0  0  0  0
    3.0412   -0.8853   -0.8429 C   0  0  0  0  0  0  0  0  0  0  0  0
    3.6815   -1.4309    0.2713 C   0  0  0  0  0  0  0  0  0  0  0  0
    3.3137   -2.6806    0.7790 C   0  0  0  0  0  0  0  0  0  0  0  0
    2.2547   -3.3671    0.1871 C   0  0  0  0  0  0  0  0  0  0  0  0
    1.5544   -2.8007   -0.8845 C   0  0  0  0  0  0  0  0  0  0  0  0
    1.9592   -1.5723   -1.3696 C   0  0  0  0  0  0  0  0  0  0  0  0
    4.7020   -0.7813    0.9131 O   0  0  0  0  0  0  0  0  0  0  0  0
    1.4248   -1.1488   -2.2020 H   0  0  0  0  0  0  0  0  0  0  0  0
    0.3931   -3.5588   -1.4959 C   0  0  0  0  0  0  0  0  0  0  0  0
   -0.9108   -3.0796   -0.8847 C   0  0  0  0  0  0  0  0  0  0  0  0
   -1.4904   -3.7695    0.1834 C   0  0  0  0  0  0  0  0  0  0  0  0
   -2.7476   -3.4149    0.6812 C   0  0  0  0  0  0  0  0  0  0  0  0
   -3.4109   -2.3211    0.1277 C   0  0  0  0  0  0  0  0  0  0  0  0
   -2.8114   -1.5725   -0.8914 C   0  0  0  0  0  0  0  0  0  0  0  0
   -1.5716   -1.9597   -1.3629 C   0  0  0  0  0  0  0  0  0  0  0  0
```

-0.8675	-4.8317	0.7833	O	0	0	0	0	0	0	0	0	0	0	0	0
-4.6322	-1.9010	0.5634	O	0	0	0	0	0	0	0	0	0	0	0	0
-1.1181	-1.3734	-2.1435	H	0	0	0	0	0	0	0	0	0	0	0	0
-3.5524	-0.3834	-1.4709	C	0	0	0	0	0	0	0	0	0	0	0	0
-3.0509	0.9023	-0.8402	C	0	0	0	0	0	0	0	0	0	0	0	0
-3.6965	1.4406	0.2763	C	0	0	0	0	0	0	0	0	0	0	0	0
-3.3266	2.6826	0.8014	C	0	0	0	0	0	0	0	0	0	0	0	0
-2.2605	3.3715	0.2256	C	0	0	0	0	0	0	0	0	0	0	0	0
-1.5570	2.8139	-0.8479	C	0	0	0	0	0	0	0	0	0	0	0	0
-1.9585	1.5890	-1.3449	C	0	0	0	0	0	0	0	0	0	0	0	0
-4.7283	0.7931	0.9018	O	0	0	0	0	0	0	0	0	0	0	0	0
-1.8275	4.5791	0.6851	O	0	0	0	0	0	0	0	0	0	0	0	0
-1.4090	1.1702	-2.1701	H	0	0	0	0	0	0	0	0	0	0	0	0
-0.3980	3.5832	-1.4492	C	0	0	0	0	0	0	0	0	0	0	0	0
3.5030	0.3733	-3.0037	C	0	0	0	0	0	0	0	0	0	0	0	0
0.4194	-3.5044	-3.0329	C	0	0	0	0	0	0	0	0	0	0	0	0
0.5299	-4.6138	-1.2352	H	0	0	0	0	0	0	0	0	0	0	0	0
-3.5159	-0.3921	-3.0088	C	0	0	0	0	0	0	0	0	0	0	0	0
-4.6074	-0.5077	-1.2038	H	0	0	0	0	0	0	0	0	0	0	0	0
-0.4279	3.5531	-2.9868	C	0	0	0	0	0	0	0	0	0	0	0	0
-0.5389	4.6337	-1.1733	H	0	0	0	0	0	0	0	0	0	0	0	0
3.9308	1.3119	-3.3661	H	0	0	0	0	0	0	0	0	0	0	0	0
2.4744	0.3287	-3.3600	H	0	0	0	0	0	0	0	0	0	0	0	0
4.2737	-0.8015	-3.6181	C	0	0	0	0	0	0	0	0	0	0	0	0
1.4671	-3.5057	-3.3444	H	0	0	0	0	0	0	0	0	0	0	0	0
-0.0343	-2.5874	-3.4059	H	0	0	0	0	0	0	0	0	0	0	0	0
-0.2754	-4.6970	-3.7002	C	0	0	0	0	0	0	0	0	0	0	0	0
-3.5441	-1.4371	-3.3286	H	0	0	0	0	0	0	0	0	0	0	0	0
-2.5935	0.0435	-3.3896	H	0	0	0	0	0	0	0	0	0	0	0	0
-4.7027	0.3326	-3.6545	C	0	0	0	0	0	0	0	0	0	0	0	0
-1.4772	3.5673	-3.2932	H	0	0	0	0	0	0	0	0	0	0	0	0
0.0185	2.6391	-3.3757	H	0	0	0	0	0	0	0	0	0	0	0	0
0.2725	4.7513	-3.6380	C	0	0	0	0	0	0	0	0	0	0	0	0
4.5983	0.5233	-1.2018	H	0	0	0	0	0	0	0	0	0	0	0	0
-4.0552	3.2290	2.0112	C	0	0	0	0	0	0	0	0	0	0	0	0
4.6911	0.1783	0.7172	H	0	0	0	0	0	0	0	0	0	0	0	0
0.1036	-4.7768	0.6672	H	0	0	0	0	0	0	0	0	0	0	0	0
-5.0972	-2.5617	1.1339	H	0	0	0	0	0	0	0	0	0	0	0	0
-4.7092	-0.1683	0.7153	H	0	0	0	0	0	0	0	0	0	0	0	0
-2.4673	5.0216	1.2959	H	0	0	0	0	0	0	0	0	0	0	0	0
-0.0929	4.7800	0.7223	H	0	0	0	0	0	0	0	0	0	0	0	0
1.8282	-4.5844	0.6273	O	0	0	0	0	0	0	0	0	0	0	0	0
3.3674	4.1954	1.8510	C	0	0	0	0	0	0	0	0	0	0	0	0
5.1217	2.5779	1.1069	H	0	0	0	0	0	0	0	0	0	0	0	0
4.0354	-3.2372	1.9882	C	0	0	0	0	0	0	0	0	0	0	0	0
2.4667	-5.0300	1.2374	H	0	0	0	0	0	0	0	0	0	0	0	0
4.3812	5.2464	1.4529	C	0	0	0	0	0	0	0	0	0	0	0	0
4.0276	6.5290	1.0788	C	0	0	0	0	0	0	0	0	0	0	0	0
5.0894	7.4895	0.8252	C	0	0	0	0	0	0	0	0	0	0	0	0
6.4149	7.0511	0.9537	C	0	0	0	0	0	0	0	0	0	0	0	0
6.7226	5.7798	1.2755	O	0	0	0	0	0	0	0	0	0	0	0	0
5.7502	4.8551	1.5232	C	0	0	0	0	0	0	0	0	0	0	0	0
6.1368	3.7210	1.7909	O	0	0	0	0	0	0	0	0	0	0	0	0
2.7442	6.9095	0.9876	N	0	0	0	0	0	0	0	0	0	0	0	0
4.8742	8.8298	0.4806	C	0	0	0	0	0	0	0	0	0	0	0	0
5.9293	9.6889	0.2754	C	0	0	0	0	0	0	0	0	0	0	0	0
7.2383	9.2368	0.4073	C	0	0	0	0	0	0	0	0	0	0	0	0
7.4820	7.9249	0.7444	C	0	0	0	0	0	0	0	0	0	0	0	0
5.1109	-4.2532	1.6678	C	0	0	0	0	0	0	0	0	0	0	0	0
6.4055	-3.9000	1.3370	C	0	0	0	0	0	0	0	0	0	0	0	0
7.3841	-4.9603	1.1570	C	0	0	0	0	0	0	0	0	0	0	0	0
6.9515	-6.2850	1.3102	C	0	0	0	0	0	0	0	0	0	0	0	0
5.6706	-6.5940	1.5900	O	0	0	0	0	0	0	0	0	0	0	0	0
4.7285	-5.6229	1.7669	C	0	0	0	0	0	0	0	0	0	0	0	0
3.5874	-6.0116	1.9999	O	0	0	0	0	0	0	0	0	0	0	0	0
8.7353	-4.7436	0.8590	C	0	0	0	0	0	0	0	0	0	0	0	0
9.6103	-5.7968	0.7218	C	0	0	0	0	0	0	0	0	0	0	0	0
9.1637	-7.1051	0.8777	C	0	0	0	0	0	0	0	0	0	0	0	0
7.8415	-7.3501	1.1701	C	0	0	0	0	0	0	0	0	0	0	0	0
6.7791	-2.6166	1.2204	N	0	0	0	0	0	0	0	0	0	0	0	0
-3.3365	-4.1879	1.8423	C	0	0	0	0	0	0	0	0	0	0	0	0
-4.3595	-5.2326	1.4508	C	0	0	0	0	0	0	0	0	0	0	0	0
-6.4064	-7.0274	0.9692	C	0	0	0	0	0	0	0	0	0	0	0	0
-6.7050	-5.7576	1.3051	O	0	0	0	0	0	0	0	0	0	0	0	0
-5.7261	-4.8379	1.5449	C	0	0	0	0	0	0	0	0	0	0	0	0
-6.1050	-3.7046	1.8269	O	0	0	0	0	0	0	0	0	0	0	0	0
-4.0158	-6.5137	1.0624	C	0	0	0	0	0	0	0	0	0	0	0	0

-5.0844	-7.4689	0.8176	C	0	0	0	0	0	0	0	0	0	0	0	0
-2.7351	-6.8977	0.9498	N	0	0	0	0	0	0	0	0	0	0	0	0
-4.8787	-8.8072	0.4600	C	0	0	0	0	0	0	0	0	0	0	0	0
-5.9396	-9.6615	0.2642	C	0	0	0	0	0	0	0	0	0	0	0	0
-7.2449	-9.2063	0.4190	C	0	0	0	0	0	0	0	0	0	0	0	0
-7.4793	-7.8963	0.7694	C	0	0	0	0	0	0	0	0	0	0	0	0
-5.1205	4.2565	1.6934	C	0	0	0	0	0	0	0	0	0	0	0	0
-6.9434	6.3056	1.3429	C	0	0	0	0	0	0	0	0	0	0	0	0
-5.6631	6.6020	1.6382	O	0	0	0	0	0	0	0	0	0	0	0	0
-4.7296	5.6222	1.8128	C	0	0	0	0	0	0	0	0	0	0	0	0
-3.5882	6.0001	2.0617	O	0	0	0	0	0	0	0	0	0	0	0	0
-6.4144	3.9165	1.3464	C	0	0	0	0	0	0	0	0	0	0	0	0
-7.3839	4.9858	1.1704	C	0	0	0	0	0	0	0	0	0	0	0	0
-6.7963	2.6374	1.2116	N	0	0	0	0	0	0	0	0	0	0	0	0
-8.7340	4.7824	0.8584	C	0	0	0	0	0	0	0	0	0	0	0	0
-9.6003	5.8433	0.7259	C	0	0	0	0	0	0	0	0	0	0	0	0
-9.1459	7.1465	0.9006	C	0	0	0	0	0	0	0	0	0	0	0	0
-7.8245	7.3785	1.2074	C	0	0	0	0	0	0	0	0	0	0	0	0
-3.3316	3.7010	2.6816	H	0	0	0	0	0	0	0	0	0	0	0	0
-4.5086	2.3922	2.5442	H	0	0	0	0	0	0	0	0	0	0	0	0
3.8677	3.4924	2.5228	H	0	0	0	0	0	0	0	0	0	0	0	0
2.5561	4.6688	2.4058	H	0	0	0	0	0	0	0	0	0	0	0	0
3.3095	-3.7223	2.6467	H	0	0	0	0	0	0	0	0	0	0	0	0
4.4780	-2.4037	2.5353	H	0	0	0	0	0	0	0	0	0	0	0	0
2.5087	7.8234	0.6497	H	0	0	0	0	0	0	0	0	0	0	0	0
3.8690	9.2094	0.3765	H	0	0	0	0	0	0	0	0	0	0	0	0
5.7399	10.7182	0.0122	H	0	0	0	0	0	0	0	0	0	0	0	0
8.0627	9.9140	0.2463	H	0	0	0	0	0	0	0	0	0	0	0	0
8.4844	7.5437	0.8552	H	0	0	0	0	0	0	0	0	0	0	0	0
9.1107	-3.7388	0.7371	H	0	0	0	0	0	0	0	0	0	0	0	0
10.6477	-5.6063	0.4932	H	0	0	0	0	0	0	0	0	0	0	0	0
9.8534	-7.9279	0.7701	H	0	0	0	0	0	0	0	0	0	0	0	0
7.4641	-8.3519	1.2977	H	0	0	0	0	0	0	0	0	0	0	0	0
7.7062	-2.3825	0.9195	H	0	0	0	0	0	0	0	0	0	0	0	0
-3.8251	-3.4885	2.5264	H	0	0	0	0	0	0	0	0	0	0	0	0
-2.5194	-4.6683	2.3822	H	0	0	0	0	0	0	0	0	0	0	0	0
-2.5076	-7.8099	0.6020	H	0	0	0	0	0	0	0	0	0	0	0	0
-3.8764	-9.1892	0.3379	H	0	0	0	0	0	0	0	0	0	0	0	0
-5.7574	-10.6895	-0.0095	H	0	0	0	0	0	0	0	0	0	0	0	0
-8.0738	-9.8798	0.2655	H	0	0	0	0	0	0	0	0	0	0	0	0
-8.4787	-7.5128	0.8982	H	0	0	0	0	0	0	0	0	0	0	0	0
-7.7210	2.4142	0.8953	H	0	0	0	0	0	0	0	0	0	0	0	0
-9.1155	3.7817	0.7221	H	0	0	0	0	0	0	0	0	0	0	0	0
-10.6370	5.6629	0.4864	H	0	0	0	0	0	0	0	0	0	0	0	0
-9.8287	7.9754	0.7966	H	0	0	0	0	0	0	0	0	0	0	0	0
-7.4412	8.3761	1.3499	H	0	0	0	0	0	0	0	0	0	0	0	0
4.0734	-0.8051	-5.1332	C	0	0	0	0	0	0	0	0	0	0	0	0
5.7638	-0.7456	-3.2891	C	0	0	0	0	0	0	0	0	0	0	0	0
-0.0050	4.7489	-5.1414	C	0	0	0	0	0	0	0	0	0	0	0	0
1.7789	4.7473	-3.3905	C	0	0	0	0	0	0	0	0	0	0	0	0
-4.6985	0.0776	-5.1618	C	0	0	0	0	0	0	0	0	0	0	0	0
-4.6826	1.8347	-3.3844	C	0	0	0	0	0	0	0	0	0	0	0	0
-0.0038	-4.6694	-5.2044	C	0	0	0	0	0	0	0	0	0	0	0	0
-1.7807	-4.7065	-3.4469	C	0	0	0	0	0	0	0	0	0	0	0	0
3.8657	-1.7334	-3.2117	H	0	0	0	0	0	0	0	0	0	0	0	0
0.1522	-5.6205	-3.2919	H	0	0	0	0	0	0	0	0	0	0	0	0
-5.6287	-0.0831	-3.2398	H	0	0	0	0	0	0	0	0	0	0	0	0
-0.1480	5.6709	-3.2139	H	0	0	0	0	0	0	0	0	0	0	0	0
3.0171	-0.8786	-5.3831	H	0	0	0	0	0	0	0	0	0	0	0	0
4.4666	0.1107	-5.5702	H	0	0	0	0	0	0	0	0	0	0	0	0
4.5889	-1.6490	-5.5863	H	0	0	0	0	0	0	0	0	0	0	0	0
6.1823	0.2174	-3.5751	H	0	0	0	0	0	0	0	0	0	0	0	0
5.9363	-0.8995	-2.2274	H	0	0	0	0	0	0	0	0	0	0	0	0
6.2965	-1.5249	-3.8302	H	0	0	0	0	0	0	0	0	0	0	0	0
0.3993	3.8483	-5.5993	H	0	0	0	0	0	0	0	0	0	0	0	0
-1.0745	4.7840	-5.3376	H	0	0	0	0	0	0	0	0	0	0	0	0
0.4578	5.6103	-5.6177	H	0	0	0	0	0	0	0	0	0	0	0	0
2.0049	4.8353	-2.3319	H	0	0	0	0	0	0	0	0	0	0	0	0
2.2201	3.8216	-3.7534	H	0	0	0	0	0	0	0	0	0	0	0	0
2.2474	5.5794	-3.9128	H	0	0	0	0	0	0	0	0	0	0	0	0
-3.7905	0.4758	-5.6106	H	0	0	0	0	0	0	0	0	0	0	0	0
-4.7478	-0.9882	-5.3741	H	0	0	0	0	0	0	0	0	0	0	0	0
-5.5514	0.5597	-5.6342	H	0	0	0	0	0	0	0	0	0	0	0	0
-4.7536	2.0454	-2.3212	H	0	0	0	0	0	0	0	0	0	0	0	0
-3.7584	2.2732	-3.7547	H	0	0	0	0	0	0	0	0	0	0	0	0
-5.5173	2.3183	-3.8882	H	0	0	0	0	0	0	0	0	0	0	0	0
-0.4158	-3.7644	-5.6466	H	0	0	0	0	0	0	0	0	0	0	0	0

```
   1.0652   -4.6934   -5.4049 H   0  0  0  0  0  0  0  0  0  0  0  0
  -0.4625   -5.5263   -5.6925 H   0  0  0  0  0  0  0  0  0  0  0  0
  -2.0021   -4.7994   -2.3877 H   0  0  0  0  0  0  0  0  0  0  0  0
  -2.2303   -3.7831   -3.8059 H   0  0  0  0  0  0  0  0  0  0  0  0
  -2.2449   -5.5401   -3.9704 H   0  0  0  0  0  0  0  0  0  0  0  0
   2.0076    6.2147    1.0326 H   0  0  0  0  0  0  0  0  0  0  0  0
   6.0763   -1.8861    1.2135 H   0  0  0  0  0  0  0  0  0  0  0  0
  -1.9959   -6.2053    0.9886 H   0  0  0  0  0  0  0  0  0  0  0  0
  -6.0988    1.9018    1.2001 H   0  0  0  0  0  0  0  0  0  0  0  0
  1  2  1  0  0  0  0
  2  3  1  0  0  0  0
  2  7  1  0  0  0  0
  3 68  1  0  0  0  0
  4  8  1  0  0  0  0
  4  3  1  0  0  0  0
  5  4  1  0  0  0  0
  6  5  1  0  0  0  0
  6  1  1  0  0  0  0
  8 69  1  0  0  0  0
  9  6  1  0  0  0  0
 10 59  1  0  0  0  0
 10 11  1  0  0  0  0
 10  5  1  0  0  0  0
 11 12  1  0  0  0  0
 12 13  1  0  0  0  0
 12 17  1  0  0  0  0
 13 70  1  0  0  0  0
 14 67  1  0  0  0  0
 14 13  1  0  0  0  0
 15 14  1  0  0  0  0
 16 15  1  0  0  0  0
 16 11  1  0  0  0  0
 18 16  1  0  0  0  0
 19 42  1  0  0  0  0
 19 15  1  0  0  0  0
 19 20  1  0  0  0  0
 20 21  1  0  0  0  0
 21 26  1  0  0  0  0
 21 22  1  0  0  0  0
 22 96  1  0  0  0  0
 23 22  1  0  0  0  0
 23 27  1  0  0  0  0
 24 23  1  0  0  0  0
 25 20  1  0  0  0  0
 25 24  1  0  0  0  0
 27 63  1  0  0  0  0
 28 25  1  0  0  0  0
 29 44  1  0  0  0  0
 29 24  1  0  0  0  0
 29 30  1  0  0  0  0
 30 31  1  0  0  0  0
 31 32  1  0  0  0  0
 31 36  1  0  0  0  0
 32 60  1  0  0  0  0
 33 37  1  0  0  0  0
 33 32  1  0  0  0  0
 34 33  1  0  0  0  0
 35 30  1  0  0  0  0
 35 34  1  0  0  0  0
 37 65  1  0  0  0  0
 38 35  1  0  0  0  0
 39 46  1  0  0  0  0
 39  1  1  0  0  0  0
 39 34  1  0  0  0  0
 40 10  1  0  0  0  0
 41 19  1  0  0  0  0
 43 29  1  0  0  0  0
 45 39  1  0  0  0  0
 47 40  1  0  0  0  0
 48 40  1  0  0  0  0
 49150  1  0  0  0  0
 49157  1  0  0  0  0
 49 40  1  0  0  0  0
 50 41  1  0  0  0  0
 51 41  1  0  0  0  0
 52156  1  0  0  0  0
 52158  1  0  0  0  0
```

```
 52 41  1  0  0  0  0
 53 43  1  0  0  0  0
 54 43  1  0  0  0  0
 55154  1  0  0  0  0
 55159  1  0  0  0  0
 55 43  1  0  0  0  0
 56 45  1  0  0  0  0
 57 45  1  0  0  0  0
 58152  1  0  0  0  0
 58160  1  0  0  0  0
 58 45  1  0  0  0  0
 60122  1  0  0  0  0
 60121  1  0  0  0  0
 61 17  1  0  0  0  0
 62 26  1  0  0  0  0
 64 36  1  0  0  0  0
 66  7  1  0  0  0  0
 67 71  1  0  0  0  0
 68124  1  0  0  0  0
 68123  1  0  0  0  0
 70126  1  0  0  0  0
 70125  1  0  0  0  0
 72 77  1  0  0  0  0
 72 68  1  0  0  0  0
 73 72  1  0  0  0  0
 74 75  1  0  0  0  0
 74 73  1  0  0  0  0
 75 76  1  0  0  0  0
 76 77  1  0  0  0  0
 77 78  1  0  0  0  0
 79185  1  0  0  0  0
 79 73  1  0  0  0  0
 80 74  1  0  0  0  0
 81 80  1  0  0  0  0
 81 82  1  0  0  0  0
 82 83  1  0  0  0  0
 83131  1  0  0  0  0
 83 75  1  0  0  0  0
 84 89  1  0  0  0  0
 84 70  1  0  0  0  0
 85 84  1  0  0  0  0
 86 87  1  0  0  0  0
 86 85  1  0  0  0  0
 87 88  1  0  0  0  0
 88 89  1  0  0  0  0
 89 90  1  0  0  0  0
 91 86  1  0  0  0  0
 92 93  1  0  0  0  0
 92 91  1  0  0  0  0
 93 94  1  0  0  0  0
 94135  1  0  0  0  0
 94 87  1  0  0  0  0
 95 85  1  0  0  0  0
 95186  1  0  0  0  0
 96138  1  0  0  0  0
 96137  1  0  0  0  0
 97100  1  0  0  0  0
 97 96  1  0  0  0  0
 98 99  1  0  0  0  0
 99100  1  0  0  0  0
100101  1  0  0  0  0
102 97  1  0  0  0  0
103102  1  0  0  0  0
103 98  1  0  0  0  0
104187  1  0  0  0  0
104102  1  0  0  0  0
105103  1  0  0  0  0
106105  1  0  0  0  0
106107  1  0  0  0  0
107108  1  0  0  0  0
108 98  1  0  0  0  0
108143  1  0  0  0  0
109112  1  0  0  0  0
109 60  1  0  0  0  0
110111  1  0  0  0  0
111112  1  0  0  0  0
112113  1  0  0  0  0
```

```
114109  1  0  0  0  0
115110  1  0  0  0  0
115114  1  0  0  0  0
116114  1  0  0  0  0
116188  1  0  0  0  0
117115  1  0  0  0  0
118119  1  0  0  0  0
118117  1  0  0  0  0
119120  1  0  0  0  0
120148  1  0  0  0  0
120110  1  0  0  0  0
127 79  1  0  0  0  0
128 80  1  0  0  0  0
129 81  1  0  0  0  0
130 82  1  0  0  0  0
132 91  1  0  0  0  0
133 92  1  0  0  0  0
134 93  1  0  0  0  0
136 95  1  0  0  0  0
139104  1  0  0  0  0
140105  1  0  0  0  0
141106  1  0  0  0  0
142107  1  0  0  0  0
144116  1  0  0  0  0
145117  1  0  0  0  0
146118  1  0  0  0  0
147119  1  0  0  0  0
149 49  1  0  0  0  0
150165  1  0  0  0  0
151 58  1  0  0  0  0
152170  1  0  0  0  0
153 55  1  0  0  0  0
154176  1  0  0  0  0
155 52  1  0  0  0  0
156182  1  0  0  0  0
161149  1  0  0  0  0
162149  1  0  0  0  0
163149  1  0  0  0  0
164150  1  0  0  0  0
166150  1  0  0  0  0
167151  1  0  0  0  0
168151  1  0  0  0  0
169151  1  0  0  0  0
171152  1  0  0  0  0
172152  1  0  0  0  0
173153  1  0  0  0  0
174153  1  0  0  0  0
175153  1  0  0  0  0
177154  1  0  0  0  0
178154  1  0  0  0  0
179155  1  0  0  0  0
180155  1  0  0  0  0
181155  1  0  0  0  0
183156  1  0  0  0  0
184156  1  0  0  0  0
M  END
```

Crown -*out*- $CHCl_3$
E = -299.913255895117 Eh

```
188200  0     0  0            999 V2000
    0.8955    3.1149   -0.8559 C   0  0  0  0  0  0  0  0  0  0  0  0
    1.4938    3.8136    0.1954 C   0  0  0  0  0  0  0  0  0  0  0  0
    2.7662    3.4728    0.6651 C   0  0  0  0  0  0  0  0  0  0  0  0
    3.4230    2.3832    0.0965 C   0  0  0  0  0  0  0  0  0  0  0  0
    2.8093    1.6223   -0.9055 C   0  0  0  0  0  0  0  0  0  0  0  0
    1.5545    1.9985   -1.3458 C   0  0  0  0  0  0  0  0  0  0  0  0
    0.8731    4.8732    0.8090 O   0  0  0  0  0  0  0  0  0  0  0  0
    4.6629    1.9740    0.5096 O   0  0  0  0  0  0  0  0  0  0  0  0
    1.0789    1.4060   -2.1067 H   0  0  0  0  0  0  0  0  0  0  0  0
    3.5453    0.4282   -1.4835 C   0  0  0  0  0  0  0  0  0  0  0  0
    3.0515   -0.8616   -0.8542 C   0  0  0  0  0  0  0  0  0  0  0  0
```

3.6898	-1.3936	0.2680	C	0	0	0	0	0	0	0	0	0	0	0	0
3.3244	-2.6382	0.7919	C	0	0	0	0	0	0	0	0	0	0	0	0
2.2735	-3.3358	0.2002	C	0	0	0	0	0	0	0	0	0	0	0	0
1.5774	-2.7906	-0.8844	C	0	0	0	0	0	0	0	0	0	0	0	0
1.9785	-1.5641	-1.3800	C	0	0	0	0	0	0	0	0	0	0	0	0
4.7127	-0.7386	0.9062	O	0	0	0	0	0	0	0	0	0	0	0	0
1.4478	-1.1565	-2.2213	H	0	0	0	0	0	0	0	0	0	0	0	0
0.4283	-3.5668	-1.4995	C	0	0	0	0	0	0	0	0	0	0	0	0
-0.8859	-3.1076	-0.8929	C	0	0	0	0	0	0	0	0	0	0	0	0
-1.4722	-3.8159	0.1593	C	0	0	0	0	0	0	0	0	0	0	0	0
-2.7339	-3.4734	0.6558	C	0	0	0	0	0	0	0	0	0	0	0	0
-3.3958	-2.3752	0.1108	C	0	0	0	0	0	0	0	0	0	0	0	0
-2.7967	-1.6103	-0.8959	C	0	0	0	0	0	0	0	0	0	0	0	0
-1.5507	-1.9859	-1.3626	C	0	0	0	0	0	0	0	0	0	0	0	0
-0.8526	-4.8912	0.7460	O	0	0	0	0	0	0	0	0	0	0	0	0
-4.6292	-1.9683	0.5450	O	0	0	0	0	0	0	0	0	0	0	0	0
-1.0955	-1.3890	-2.1329	H	0	0	0	0	0	0	0	0	0	0	0	0
-3.5428	-0.4224	-1.4750	C	0	0	0	0	0	0	0	0	0	0	0	0
-3.0524	0.8656	-0.8381	C	0	0	0	0	0	0	0	0	0	0	0	0
-3.7010	1.3931	0.2818	C	0	0	0	0	0	0	0	0	0	0	0	0
-3.3347	2.6304	0.8217	C	0	0	0	0	0	0	0	0	0	0	0	0
-2.2736	3.3282	0.2488	C	0	0	0	0	0	0	0	0	0	0	0	0
-1.5708	2.7896	-0.8343	C	0	0	0	0	0	0	0	0	0	0	0	0
-1.9664	1.5654	-1.3398	C	0	0	0	0	0	0	0	0	0	0	0	0
-4.7393	0.7419	0.8993	O	0	0	0	0	0	0	0	0	0	0	0	0
-1.8483	4.5410	0.7209	O	0	0	0	0	0	0	0	0	0	0	0	0
-1.4183	1.1594	-2.1710	H	0	0	0	0	0	0	0	0	0	0	0	0
-0.4258	3.5771	-1.4420	C	0	0	0	0	0	0	0	0	0	0	0	0
3.4933	0.4092	-3.0190	C	0	0	0	0	0	0	0	0	0	0	0	0
0.4603	-3.5135	-3.0375	C	0	0	0	0	0	0	0	0	0	0	0	0
0.5813	-4.6184	-1.2343	H	0	0	0	0	0	0	0	0	0	0	0	0
-3.5007	-0.4324	-3.0137	C	0	0	0	0	0	0	0	0	0	0	0	0
-4.5972	-0.5546	-1.2094	H	0	0	0	0	0	0	0	0	0	0	0	0
-0.4703	3.5507	-2.9805	C	0	0	0	0	0	0	0	0	0	0	0	0
-0.5811	4.6236	-1.1590	H	0	0	0	0	0	0	0	0	0	0	0	0
3.8617	1.3740	-3.3758	H	0	0	0	0	0	0	0	0	0	0	0	0
2.4661	0.3052	-3.3631	H	0	0	0	0	0	0	0	0	0	0	0	0
4.3296	-0.7131	-3.6471	C	0	0	0	0	0	0	0	0	0	0	0	0
1.5096	-3.4853	-3.3394	H	0	0	0	0	0	0	0	0	0	0	0	0
-0.0199	-2.6112	-3.4107	H	0	0	0	0	0	0	0	0	0	0	0	0
-0.1941	-4.7272	-3.7088	C	0	0	0	0	0	0	0	0	0	0	0	0
-3.5061	-1.4787	-3.3278	H	0	0	0	0	0	0	0	0	0	0	0	0
-2.5867	0.0223	-3.3899	H	0	0	0	0	0	0	0	0	0	0	0	0
-4.7010	0.2642	-3.6666	C	0	0	0	0	0	0	0	0	0	0	0	0
-1.5226	3.5262	-3.2725	H	0	0	0	0	0	0	0	0	0	0	0	0
0.0082	2.6562	-3.3743	H	0	0	0	0	0	0	0	0	0	0	0	0
0.1756	4.7779	-3.6357	C	0	0	0	0	0	0	0	0	0	0	0	0
4.5990	0.5549	-1.2199	H	0	0	0	0	0	0	0	0	0	0	0	0
-4.0590	3.1582	2.0426	C	0	0	0	0	0	0	0	0	0	0	0	0
4.7115	0.2151	0.6893	H	0	0	0	0	0	0	0	0	0	0	0	0
0.1180	-4.8250	0.6445	H	0	0	0	0	0	0	0	0	0	0	0	0
-5.0694	-2.6224	1.1377	H	0	0	0	0	0	0	0	0	0	0	0	0
-4.7254	-0.2148	0.6967	H	0	0	0	0	0	0	0	0	0	0	0	0
-2.4650	4.9414	1.3782	H	0	0	0	0	0	0	0	0	0	0	0	0
-0.0976	4.8049	0.7120	H	0	0	0	0	0	0	0	0	0	0	0	0
1.8542	-4.5577	0.6537	O	0	0	0	0	0	0	0	0	0	0	0	0
3.3739	4.2513	1.8134	C	0	0	0	0	0	0	0	0	0	0	0	0
5.1170	2.6311	1.0888	H	0	0	0	0	0	0	0	0	0	0	0	0
4.0344	-3.1719	2.0187	C	0	0	0	0	0	0	0	0	0	0	0	0
2.4666	-4.9602	1.3141	H	0	0	0	0	0	0	0	0	0	0	0	0
4.4014	5.2881	1.4087	C	0	0	0	0	0	0	0	0	0	0	0	0
4.0598	6.5721	1.0092	C	0	0	0	0	0	0	0	0	0	0	0	0
5.1307	7.5207	0.7508	C	0	0	0	0	0	0	0	0	0	0	0	0
6.4512	7.0766	0.8999	C	0	0	0	0	0	0	0	0	0	0	0	0
6.7456	5.8011	1.2543	O	0	0	0	0	0	0	0	0	0	0	0	0
5.7611	4.8987	1.5094	C	0	0	0	0	0	0	0	0	0	0	0	0
6.1437	3.7606	1.8181	O	0	0	0	0	0	0	0	0	0	0	0	0
2.7853	6.9557	0.8863	N	0	0	0	0	0	0	0	0	0	0	0	0
4.9282	8.8570	0.3780	C	0	0	0	0	0	0	0	0	0	0	0	0
5.9934	9.7021	0.1665	C	0	0	0	0	0	0	0	0	0	0	0	0
7.2981	9.2419	0.3188	C	0	0	0	0	0	0	0	0	0	0	0	0
7.5291	7.9344	0.6834	C	0	0	0	0	0	0	0	0	0	0	0	0
5.0865	-4.2264	1.7439	C	0	0	0	0	0	0	0	0	0	0	0	0
6.3897	-3.9148	1.3839	C	0	0	0	0	0	0	0	0	0	0	0	0
7.3489	-5.0005	1.2631	C	0	0	0	0	0	0	0	0	0	0	0	0
6.8974	-6.3048	1.5038	C	0	0	0	0	0	0	0	0	0	0	0	0
5.6040	-6.5736	1.8108	O	0	0	0	0	0	0	0	0	0	0	0	0

4.6895	-5.5744	1.9311	C	0	0	0	0	0	0	0	0	0	0	0	0
3.5341	-5.9337	2.2014	O	0	0	0	0	0	0	0	0	0	0	0	0
8.7025	-4.8254	0.9420	C	0	0	0	0	0	0	0	0	0	0	0	0
9.5576	-5.9011	0.8700	C	0	0	0	0	0	0	0	0	0	0	0	0
9.0909	-7.1891	1.1161	C	0	0	0	0	0	0	0	0	0	0	0	0
7.7659	-7.3933	1.4304	C	0	0	0	0	0	0	0	0	0	0	0	0
6.7790	-2.6534	1.1765	N	0	0	0	0	0	0	0	0	0	0	0	0
-3.3255	-4.2625	1.8052	C	0	0	0	0	0	0	0	0	0	0	0	0
-4.3734	-5.2816	1.4081	C	0	0	0	0	0	0	0	0	0	0	0	0
-6.4537	-7.0411	0.9240	C	0	0	0	0	0	0	0	0	0	0	0	0
-6.7258	-5.7714	1.3149	O	0	0	0	0	0	0	0	0	0	0	0	0
-5.7258	-4.8820	1.5554	C	0	0	0	0	0	0	0	0	0	0	0	0
-6.0881	-3.7458	1.8943	O	0	0	0	0	0	0	0	0	0	0	0	0
-4.0554	-6.5602	0.9734	C	0	0	0	0	0	0	0	0	0	0	0	0
-5.1425	-7.4925	0.7234	C	0	0	0	0	0	0	0	0	0	0	0	0
-2.7886	-6.9534	0.8097	N	0	0	0	0	0	0	0	0	0	0	0	0
-4.9639	-8.8208	0.3117	C	0	0	0	0	0	0	0	0	0	0	0	0
-6.0426	-9.6519	0.1144	C	0	0	0	0	0	0	0	0	0	0	0	0
-7.3376	-9.1856	0.3210	C	0	0	0	0	0	0	0	0	0	0	0	0
-7.5454	-7.8851	0.7230	C	0	0	0	0	0	0	0	0	0	0	0	0
-5.0976	4.2240	1.7599	C	0	0	0	0	0	0	0	0	0	0	0	0
-6.8862	6.3201	1.5083	C	0	0	0	0	0	0	0	0	0	0	0	0
-5.5965	6.5748	1.8414	O	0	0	0	0	0	0	0	0	0	0	0	0
-4.6926	5.5667	1.9678	C	0	0	0	0	0	0	0	0	0	0	0	0
-3.5393	5.9133	2.2623	O	0	0	0	0	0	0	0	0	0	0	0	0
-6.3966	3.9270	1.3735	C	0	0	0	0	0	0	0	0	0	0	0	0
-7.3442	5.0222	1.2462	C	0	0	0	0	0	0	0	0	0	0	0	0
-6.7937	2.6711	1.1477	N	0	0	0	0	0	0	0	0	0	0	0	0
-8.6932	4.8621	0.8992	C	0	0	0	0	0	0	0	0	0	0	0	0
-9.5379	5.9456	0.8230	C	0	0	0	0	0	0	0	0	0	0	0	0
-9.0650	7.2270	1.0908	C	0	0	0	0	0	0	0	0	0	0	0	0
-7.7442	7.4167	1.4307	C	0	0	0	0	0	0	0	0	0	0	0	0
-3.3274	3.5892	2.7328	H	0	0	0	0	0	0	0	0	0	0	0	0
-4.5350	2.3162	2.5475	H	0	0	0	0	0	0	0	0	0	0	0	0
3.8651	3.5530	2.4977	H	0	0	0	0	0	0	0	0	0	0	0	0
2.5652	4.7385	2.3605	H	0	0	0	0	0	0	0	0	0	0	0	0
3.2958	-3.6150	2.6937	H	0	0	0	0	0	0	0	0	0	0	0	0
4.4949	-2.3304	2.5386	H	0	0	0	0	0	0	0	0	0	0	0	0
2.5573	7.8798	0.5668	H	0	0	0	0	0	0	0	0	0	0	0	0
3.9277	9.2409	0.2561	H	0	0	0	0	0	0	0	0	0	0	0	0
5.8158	10.7274	-0.1183	H	0	0	0	0	0	0	0	0	0	0	0	0
8.1302	9.9076	0.1524	H	0	0	0	0	0	0	0	0	0	0	0	0
8.5266	7.5452	0.8110	H	0	0	0	0	0	0	0	0	0	0	0	0
9.0913	-3.8380	0.7495	H	0	0	0	0	0	0	0	0	0	0	0	0
10.5959	-5.7445	0.6226	H	0	0	0	0	0	0	0	0	0	0	0	0
9.7648	-8.0295	1.0605	H	0	0	0	0	0	0	0	0	0	0	0	0
7.3709	-8.3775	1.6257	H	0	0	0	0	0	0	0	0	0	0	0	0
7.7218	-2.4453	0.9012	H	0	0	0	0	0	0	0	0	0	0	0	0
-3.7936	-3.5692	2.5105	H	0	0	0	0	0	0	0	0	0	0	0	0
-2.5106	-4.7667	2.3272	H	0	0	0	0	0	0	0	0	0	0	0	0
-2.5779	-7.8739	0.4686	H	0	0	0	0	0	0	0	0	0	0	0	0
-3.9713	-9.2092	0.1473	H	0	0	0	0	0	0	0	0	0	0	0	0
-5.8833	-10.6715	-0.2002	H	0	0	0	0	0	0	0	0	0	0	0	0
-8.1805	-9.8406	0.1666	H	0	0	0	0	0	0	0	0	0	0	0	0
-8.5348	-7.4910	0.8917	H	0	0	0	0	0	0	0	0	0	0	0	0
-7.7328	2.4749	0.8520	H	0	0	0	0	0	0	0	0	0	0	0	0
-9.0868	3.8800	0.6896	H	0	0	0	0	0	0	0	0	0	0	0	0
-10.5728	5.8004	0.5552	H	0	0	0	0	0	0	0	0	0	0	0	0
-9.7308	8.0737	1.0320	H	0	0	0	0	0	0	0	0	0	0	0	0
-7.3445	8.3954	1.6432	H	0	0	0	0	0	0	0	0	0	0	0	0
4.0250	-0.8034	-5.1428	C	0	0	0	0	0	0	0	0	0	0	0	0
5.8274	-0.4970	-3.4382	C	0	0	0	0	0	0	0	0	0	0	0	0
-0.1633	4.8004	-5.1270	C	0	0	0	0	0	0	0	0	0	0	0	0
1.6906	4.8004	-3.4484	C	0	0	0	0	0	0	0	0	0	0	0	0
-4.7090	-0.0285	-5.1678	C	0	0	0	0	0	0	0	0	0	0	0	0
-4.6911	1.7728	-3.4331	C	0	0	0	0	0	0	0	0	0	0	0	0
0.1332	-4.7241	-5.2028	C	0	0	0	0	0	0	0	0	0	0	0	0
-1.7077	-4.7489	-3.5103	C	0	0	0	0	0	0	0	0	0	0	0	0
4.0457	-1.6602	-3.1750	H	0	0	0	0	0	0	0	0	0	0	0	0
0.2284	-5.6380	-3.2676	H	0	0	0	0	0	0	0	0	0	0	0	0
-5.6191	-0.1495	-3.2324	H	0	0	0	0	0	0	0	0	0	0	0	0
-0.2464	5.6793	-3.1754	H	0	0	0	0	0	0	0	0	0	0	0	0
2.9685	-0.9979	-5.3122	H	0	0	0	0	0	0	0	0	0	0	0	0
4.2871	0.1291	-5.6391	H	0	0	0	0	0	0	0	0	0	0	0	0
4.5967	-1.6073	-5.6012	H	0	0	0	0	0	0	0	0	0	0	0	0
6.1335	0.4645	-3.8464	H	0	0	0	0	0	0	0	0	0	0	0	0
6.0868	-0.5219	-2.3833	H	0	0	0	0	0	0	0	0	0	0	0	0

6.3930 -1.2790 -3.9406 H 0 0 0 0 0 0 0 0 0 0 0 0
0.2293 3.9123 -5.6181 H 0 0 0 0 0 0 0 0 0 0 0 0
-1.2404 4.8306 -5.2780 H 0 0 0 0 0 0 0 0 0 0 0 0
0.2729 5.6753 -5.6041 H 0 0 0 0 0 0 0 0 0 0 0 0
1.9556 4.8312 -2.3955 H 0 0 0 0 0 0 0 0 0 0 0 0
2.1379 3.9092 -3.8828 H 0 0 0 0 0 0 0 0 0 0 0 0
2.1180 5.6735 -3.9374 H 0 0 0 0 0 0 0 0 0 0 0 0
-3.8054 0.3588 -5.6341 H 0 0 0 0 0 0 0 0 0 0 0 0
-4.7603 -1.0996 -5.3520 H 0 0 0 0 0 0 0 0 0 0 0 0
-5.5671 0.4414 -5.6434 H 0 0 0 0 0 0 0 0 0 0 0 0
-4.7301 2.0069 -2.3729 H 0 0 0 0 0 0 0 0 0 0 0 0
-3.7846 2.2116 -3.8435 H 0 0 0 0 0 0 0 0 0 0 0 0
-5.5474 2.2351 -3.9202 H 0 0 0 0 0 0 0 0 0 0 0 0
-0.2628 -3.8275 -5.6752 H 0 0 0 0 0 0 0 0 0 0 0 0
1.2091 -4.7512 -5.3624 H 0 0 0 0 0 0 0 0 0 0 0 0
-0.3070 -5.5905 -5.6917 H 0 0 0 0 0 0 0 0 0 0 0 0
-1.9651 -4.7921 -2.4559 H 0 0 0 0 0 0 0 0 0 0 0 0
-2.1560 -3.8516 -3.9311 H 0 0 0 0 0 0 0 0 0 0 0 0
-2.1411 -5.6147 -4.0072 H 0 0 0 0 0 0 0 0 0 0 0 0
2.0368 6.2798 0.9707 H 0 0 0 0 0 0 0 0 0 0 0 0
6.1023 -1.9006 1.1854 H 0 0 0 0 0 0 0 0 0 0 0 0
-2.0307 -6.2880 0.8935 H 0 0 0 0 0 0 0 0 0 0 0 0
-6.1244 1.9118 1.1592 H 0 0 0 0 0 0 0 0 0 0 0 0
1 2 1 0 0 0 0
2 3 1 0 0 0 0
2 7 1 0 0 0 0
3 68 1 0 0 0 0
4 8 1 0 0 0 0
4 3 1 0 0 0 0
5 4 1 0 0 0 0
6 5 1 0 0 0 0
6 1 1 0 0 0 0
8 69 1 0 0 0 0
9 6 1 0 0 0 0
10 59 1 0 0 0 0
10 11 1 0 0 0 0
10 5 1 0 0 0 0
11 12 1 0 0 0 0
12 13 1 0 0 0 0
12 17 1 0 0 0 0
13 70 1 0 0 0 0
14 67 1 0 0 0 0
14 13 1 0 0 0 0
15 14 1 0 0 0 0
16 15 1 0 0 0 0
16 11 1 0 0 0 0
18 16 1 0 0 0 0
19 42 1 0 0 0 0
19 15 1 0 0 0 0
19 20 1 0 0 0 0
20 21 1 0 0 0 0
21 26 1 0 0 0 0
21 22 1 0 0 0 0
22 96 1 0 0 0 0
23 22 1 0 0 0 0
23 27 1 0 0 0 0
24 23 1 0 0 0 0
25 20 1 0 0 0 0
25 24 1 0 0 0 0
27 63 1 0 0 0 0
28 25 1 0 0 0 0
29 44 1 0 0 0 0
29 24 1 0 0 0 0
29 30 1 0 0 0 0
30 31 1 0 0 0 0
31 32 1 0 0 0 0
31 36 1 0 0 0 0
32 60 1 0 0 0 0
33 37 1 0 0 0 0
33 32 1 0 0 0 0
34 33 1 0 0 0 0
35 30 1 0 0 0 0
35 34 1 0 0 0 0
37 65 1 0 0 0 0
38 35 1 0 0 0 0
39 46 1 0 0 0 0
39 1 1 0 0 0 0

39 34 1 0 0 0 0
40 10 1 0 0 0 0
41 19 1 0 0 0 0
43 29 1 0 0 0 0
45 39 1 0 0 0 0
47 40 1 0 0 0 0
48 40 1 0 0 0 0
49150 1 0 0 0 0
49157 1 0 0 0 0
49 40 1 0 0 0 0
50 41 1 0 0 0 0
51 41 1 0 0 0 0
52156 1 0 0 0 0
52158 1 0 0 0 0
52 41 1 0 0 0 0
53 43 1 0 0 0 0
54 43 1 0 0 0 0
55154 1 0 0 0 0
55159 1 0 0 0 0
55 43 1 0 0 0 0
56 45 1 0 0 0 0
57 45 1 0 0 0 0
58152 1 0 0 0 0
58160 1 0 0 0 0
58 45 1 0 0 0 0
60122 1 0 0 0 0
60121 1 0 0 0 0
61 17 1 0 0 0 0
62 26 1 0 0 0 0
64 36 1 0 0 0 0
66 7 1 0 0 0 0
67 71 1 0 0 0 0
68124 1 0 0 0 0
68123 1 0 0 0 0
70126 1 0 0 0 0
70125 1 0 0 0 0
72 77 1 0 0 0 0
72 68 1 0 0 0 0
73 72 1 0 0 0 0
74 75 1 0 0 0 0
74 73 1 0 0 0 0
75 76 1 0 0 0 0
76 77 1 0 0 0 0
77 78 1 0 0 0 0
79185 1 0 0 0 0
79 73 1 0 0 0 0
80 74 1 0 0 0 0
81 80 1 0 0 0 0
81 82 1 0 0 0 0
82 83 1 0 0 0 0
83131 1 0 0 0 0
83 75 1 0 0 0 0
84 89 1 0 0 0 0
84 70 1 0 0 0 0
85 84 1 0 0 0 0
86 87 1 0 0 0 0
86 85 1 0 0 0 0
87 88 1 0 0 0 0
88 89 1 0 0 0 0
89 90 1 0 0 0 0
91 86 1 0 0 0 0
92 93 1 0 0 0 0
92 91 1 0 0 0 0
93 94 1 0 0 0 0
94135 1 0 0 0 0
94 87 1 0 0 0 0
95 85 1 0 0 0 0
95186 1 0 0 0 0
96138 1 0 0 0 0
96137 1 0 0 0 0
97100 1 0 0 0 0
97 96 1 0 0 0 0
98 99 1 0 0 0 0
99100 1 0 0 0 0
100101 1 0 0 0 0
102 97 1 0 0 0 0
103102 1 0 0 0 0

103 98 1 0 0 0 0
104187 1 0 0 0 0
104102 1 0 0 0 0
105103 1 0 0 0 0
106105 1 0 0 0 0
106107 1 0 0 0 0
107108 1 0 0 0 0
108 98 1 0 0 0 0
108143 1 0 0 0 0
109112 1 0 0 0 0
109 60 1 0 0 0 0
110111 1 0 0 0 0
111112 1 0 0 0 0
112113 1 0 0 0 0
114109 1 0 0 0 0
115110 1 0 0 0 0
115114 1 0 0 0 0
116114 1 0 0 0 0
116188 1 0 0 0 0
117115 1 0 0 0 0
118119 1 0 0 0 0
118117 1 0 0 0 0
119120 1 0 0 0 0
120148 1 0 0 0 0
120110 1 0 0 0 0
127 79 1 0 0 0 0
128 80 1 0 0 0 0
129 81 1 0 0 0 0
130 82 1 0 0 0 0
132 91 1 0 0 0 0
133 92 1 0 0 0 0
134 93 1 0 0 0 0
136 95 1 0 0 0 0
139104 1 0 0 0 0
140105 1 0 0 0 0
141106 1 0 0 0 0
142107 1 0 0 0 0
144116 1 0 0 0 0
145117 1 0 0 0 0
146118 1 0 0 0 0
147119 1 0 0 0 0
149 49 1 0 0 0 0
150165 1 0 0 0 0
151 58 1 0 0 0 0
152170 1 0 0 0 0
153 55 1 0 0 0 0
154176 1 0 0 0 0
155 52 1 0 0 0 0
156182 1 0 0 0 0
161149 1 0 0 0 0
162149 1 0 0 0 0
163149 1 0 0 0 0
164150 1 0 0 0 0
166150 1 0 0 0 0
167151 1 0 0 0 0
168151 1 0 0 0 0
169151 1 0 0 0 0
171152 1 0 0 0 0
172152 1 0 0 0 0
173153 1 0 0 0 0
174153 1 0 0 0 0
175153 1 0 0 0 0
177154 1 0 0 0 0
178154 1 0 0 0 0
179155 1 0 0 0 0
180155 1 0 0 0 0
181155 1 0 0 0 0
183156 1 0 0 0 0
184156 1 0 0 0 0
M END

Crown-*out*-DMSO
E = -299.909515052314 Eh

188200 0   0 0   999 V2000
0.9129   3.1106   -0.8382 C   0 0 0 0 0 0 0 0 0 0 0 0
1.5176   3.8144   0.2063 C   0 0 0 0 0 0 0 0 0 0 0 0
2.7871   3.4647   0.6784 C   0 0 0 0 0 0 0 0 0 0 0 0
3.4363   2.3638   0.1216 C   0 0 0 0 0 0 0 0 0 0 0 0
2.8160   1.6041   -0.8783 C   0 0 0 0 0 0 0 0 0 0 0 0
1.5642   1.9869   -1.3203 C   0 0 0 0 0 0 0 0 0 0 0 0
0.9096   4.8844   0.8090 O   0 0 0 0 0 0 0 0 0 0 0 0
4.6652   1.9401   0.5429 O   0 0 0 0 0 0 0 0 0 0 0 0
1.0847   1.3937   -2.0797 H   0 0 0 0 0 0 0 0 0 0 0 0
3.5452   0.4082   -1.4598 C   0 0 0 0 0 0 0 0 0 0 0 0
3.0521   -0.8862   -0.8406 C   0 0 0 0 0 0 0 0 0 0 0 0
3.7027   -1.4343   0.2674 C   0 0 0 0 0 0 0 0 0 0 0 0
3.3335   -2.6814   0.7834 C   0 0 0 0 0 0 0 0 0 0 0 0
2.2664   -3.3660   0.2043 C   0 0 0 0 0 0 0 0 0 0 0 0
1.5641   -2.8053   -0.8695 C   0 0 0 0 0 0 0 0 0 0 0 0
1.9689   -1.5780   -1.3590 C   0 0 0 0 0 0 0 0 0 0 0 0
4.7398   -0.7972   0.8957 O   0 0 0 0 0 0 0 0 0 0 0 0
1.4298   -1.1593   -2.1907 H   0 0 0 0 0 0 0 0 0 0 0 0
0.4054   -3.5688   -1.4817 C   0 0 0 0 0 0 0 0 0 0 0 0
-0.9066   -3.1020   -0.8784 C   0 0 0 0 0 0 0 0 0 0 0 0
-1.5016   -3.8129   0.1675 C   0 0 0 0 0 0 0 0 0 0 0 0
-2.7626   -3.4621   0.6612 C   0 0 0 0 0 0 0 0 0 0 0 0
-3.4162   -2.3548   0.1228 C   0 0 0 0 0 0 0 0 0 0 0 0
-2.8076   -1.5919   -0.8809 C   0 0 0 0 0 0 0 0 0 0 0 0
-1.5630   -1.9742   -1.3447 C   0 0 0 0 0 0 0 0 0 0 0 0
-0.8949   -4.8949   0.7492 O   0 0 0 0 0 0 0 0 0 0 0 0
-4.6406   -1.9347   0.5600 O   0 0 0 0 0 0 0 0 0 0 0 0
-1.1008   -1.3776   -2.1125 H   0 0 0 0 0 0 0 0 0 0 0 0
-3.5453   -0.3998   -1.4612 C   0 0 0 0 0 0 0 0 0 0 0 0
-3.0550   0.8892   -0.8279 C   0 0 0 0 0 0 0 0 0 0 0 0
-3.7171   1.4319   0.2769 C   0 0 0 0 0 0 0 0 0 0 0 0
-3.3474   2.6715   0.8104 C   0 0 0 0 0 0 0 0 0 0 0 0
-2.2705   3.3571   0.2509 C   0 0 0 0 0 0 0 0 0 0 0 0
-1.5596   2.8030   -0.8203 C   0 0 0 0 0 0 0 0 0 0 0 0
-1.9576   1.5777   -1.3199 C   0 0 0 0 0 0 0 0 0 0 0 0
-4.7692   0.7976   0.8829 O   0 0 0 0 0 0 0 0 0 0 0 0
-1.8357   4.5630   0.7230 O   0 0 0 0 0 0 0 0 0 0 0 0
-1.4011   1.1593   -2.1406 H   0 0 0 0 0 0 0 0 0 0 0 0
-0.4051   3.5781   -1.4252 C   0 0 0 0 0 0 0 0 0 0 0 0
3.4896   0.4002   -2.9952 C   0 0 0 0 0 0 0 0 0 0 0 0
0.4394   -3.5176   -3.0198 C   0 0 0 0 0 0 0 0 0 0 0 0
0.5485   -4.6215   -1.2187 H   0 0 0 0 0 0 0 0 0 0 0 0
-3.4986   -0.4123   -2.9999 C   0 0 0 0 0 0 0 0 0 0 0 0
-4.6009   -0.5269   -1.2008 H   0 0 0 0 0 0 0 0 0 0 0 0
-0.4499   3.5476   -2.9636 C   0 0 0 0 0 0 0 0 0 0 0 0
-0.5520   4.6266   -1.1481 H   0 0 0 0 0 0 0 0 0 0 0 0
3.8274   1.3770   -3.3507 H   0 0 0 0 0 0 0 0 0 0 0 0
2.4645   0.2682   -3.3378 H   0 0 0 0 0 0 0 0 0 0 0 0
4.3557   -0.6948   -3.6301 C   0 0 0 0 0 0 0 0 0 0 0 0
1.4892   -3.5106   -3.3233 H   0 0 0 0 0 0 0 0 0 0 0 0
-0.0201   -2.6051   -3.3960 H   0 0 0 0 0 0 0 0 0 0 0 0
-0.2409   -4.7168   -3.6915 C   0 0 0 0 0 0 0 0 0 0 0 0
-3.5121   -1.4587   -3.3154 H   0 0 0 0 0 0 0 0 0 0 0 0
-2.5786   0.0326   -3.3750 H   0 0 0 0 0 0 0 0 0 0 0 0
-4.6882   0.2952   -3.6600 C   0 0 0 0 0 0 0 0 0 0 0 0
-1.5028   3.5508   -3.2568 H   0 0 0 0 0 0 0 0 0 0 0 0
0.0013   2.6376   -3.3558 H   0 0 0 0 0 0 0 0 0 0 0 0
0.2302   4.7517   -3.6266 C   0 0 0 0 0 0 0 0 0 0 0 0
4.5998   0.5285   -1.1991 H   0 0 0 0 0 0 0 0 0 0 0 0
-4.0848   3.2109   2.0187 C   0 0 0 0 0 0 0 0 0 0 0 0
4.7315   0.1663   0.7281 H   0 0 0 0 0 0 0 0 0 0 0 0
0.0791   -4.8374   0.6791 H   0 0 0 0 0 0 0 0 0 0 0 0
-5.1023   -2.5930   1.1259 H   0 0 0 0 0 0 0 0 0 0 0 0
-4.7531   -0.1673   0.7237 H   0 0 0 0 0 0 0 0 0 0 0 0
-2.4623   4.9858   1.3513 H   0 0 0 0 0 0 0 0 0 0 0 0
-0.0643   4.8233   0.7439 H   0 0 0 0 0 0 0 0 0 0 0 0
1.8350   -4.5800   0.6588 O   0 0 0 0 0 0 0 0 0 0 0 0
3.3955   4.2455   1.8254 C   0 0 0 0 0 0 0 0 0 0 0 0
5.1390   2.6000   1.0969 H   0 0 0 0 0 0 0 0 0 0 0 0
4.0563   -3.2288   1.9971 C   0 0 0 0 0 0 0 0 0 0 0 0
2.4566   -5.0052   1.2906 H   0 0 0 0 0 0 0 0 0 0 0 0
4.4132   5.2942   1.4270 C   0 0 0 0 0 0 0 0 0 0 0 0
4.0619   6.5795   1.0447 C   0 0 0 0 0 0 0 0 0 0 0 0
5.1275   7.5387   0.7935 C   0 0 0 0 0 0 0 0 0 0 0 0
6.4521   7.1019   0.9328 C   0 0 0 0 0 0 0 0 0 0 0 0

```
6.7578 5.8301 1.2733 O 0 0 0 0 0 0 0 0 0 0 0 0
5.7786 4.9113 1.5230 C 0 0 0 0 0 0 0 0 0 0 0 0
6.1754 3.7814 1.8219 O 0 0 0 0 0 0 0 0 0 0 0 0
2.7833 6.9587 0.9351 N 0 0 0 0 0 0 0 0 0 0 0 0
4.9165 8.8775 0.4365 C 0 0 0 0 0 0 0 0 0 0 0 0
5.9741 9.7337 0.2284 C 0 0 0 0 0 0 0 0 0 0 0 0
7.2826 9.2812 0.3697 C 0 0 0 0 0 0 0 0 0 0 0 0
7.5227 7.9715 0.7201 C 0 0 0 0 0 0 0 0 0 0 0 0
5.1155 -4.2715 1.7057 C 0 0 0 0 0 0 0 0 0 0 0 0
6.4144 -3.9461 1.3472 C 0 0 0 0 0 0 0 0 0 0 0 0
7.3799 -5.0255 1.2034 C 0 0 0 0 0 0 0 0 0 0 0 0
6.9400 -6.3364 1.4338 C 0 0 0 0 0 0 0 0 0 0 0 0
5.6564 -6.6193 1.7496 O 0 0 0 0 0 0 0 0 0 0 0 0
4.7274 -5.6270 1.8836 C 0 0 0 0 0 0 0 0 0 0 0 0
3.5845 -6.0028 2.1593 O 0 0 0 0 0 0 0 0 0 0 0 0
8.7282 -4.8394 0.8690 C 0 0 0 0 0 0 0 0 0 0 0 0
9.5922 -5.9070 0.7758 C 0 0 0 0 0 0 0 0 0 0 0 0
9.1385 -7.2009 1.0146 C 0 0 0 0 0 0 0 0 0 0 0 0
7.8183 -7.4169 1.3407 C 0 0 0 0 0 0 0 0 0 0 0 0
6.7985 -2.6775 1.1669 N 0 0 0 0 0 0 0 0 0 0 0 0
-3.3575 -4.2506 1.8098 C 0 0 0 0 0 0 0 0 0 0 0 0
-4.3903 -5.2874 1.4192 C 0 0 0 0 0 0 0 0 0 0 0 0
-6.4515 -7.0759 0.9492 C 0 0 0 0 0 0 0 0 0 0 0 0
-6.7411 -5.8079 1.3172 O 0 0 0 0 0 0 0 0 0 0 0 0
-5.7505 -4.8979 1.5534 C 0 0 0 0 0 0 0 0 0 0 0 0
-6.1326 -3.7692 1.8752 O 0 0 0 0 0 0 0 0 0 0 0 0
-4.0560 -6.5694 1.0115 C 0 0 0 0 0 0 0 0 0 0 0 0
-5.1335 -7.5174 0.7688 C 0 0 0 0 0 0 0 0 0 0 0 0
-2.7828 -6.9563 0.8713 N 0 0 0 0 0 0 0 0 0 0 0 0
-4.9397 -8.8511 0.3839 C 0 0 0 0 0 0 0 0 0 0 0 0
-6.0072 -9.6980 0.1891 C 0 0 0 0 0 0 0 0 0 0 0 0
-7.3089 -9.2413 0.3730 C 0 0 0 0 0 0 0 0 0 0 0 0
-7.5323 -7.9361 0.7504 C 0 0 0 0 0 0 0 0 0 0 0 0
-5.1338 4.2623 1.7209 C 0 0 0 0 0 0 0 0 0 0 0 0
-6.9404 6.3415 1.4392 C 0 0 0 0 0 0 0 0 0 0 0 0
-5.6603 6.6131 1.7783 O 0 0 0 0 0 0 0 0 0 0 0 0
-4.7400 5.6136 1.9179 C 0 0 0 0 0 0 0 0 0 0 0 0
-3.5994 5.9795 2.2156 O 0 0 0 0 0 0 0 0 0 0 0 0
-6.4290 3.9485 1.3395 C 0 0 0 0 0 0 0 0 0 0 0 0
-7.3852 5.0357 1.1906 C 0 0 0 0 0 0 0 0 0 0 0 0
-6.8195 2.6842 1.1422 N 0 0 0 0 0 0 0 0 0 0 0 0
-8.7295 4.8614 0.8348 C 0 0 0 0 0 0 0 0 0 0 0 0
-9.5851 5.9354 0.7383 C 0 0 0 0 0 0 0 0 0 0 0 0
-9.1267 7.2242 0.9948 C 0 0 0 0 0 0 0 0 0 0 0 0
-7.8101 7.4286 1.3420 C 0 0 0 0 0 0 0 0 0 0 0 0
-3.3619 3.6587 2.7061 H 0 0 0 0 0 0 0 0 0 0 0 0
-4.5559 2.3714 2.5315 H 0 0 0 0 0 0 0 0 0 0 0 0
3.8954 3.5497 2.5048 H 0 0 0 0 0 0 0 0 0 0 0 0
2.5849 4.7244 2.3761 H 0 0 0 0 0 0 0 0 0 0 0 0
3.3260 -3.6870 2.6697 H 0 0 0 0 0 0 0 0 0 0 0 0
4.5149 -2.3916 2.5250 H 0 0 0 0 0 0 0 0 0 0 0 0
2.5470 7.8803 0.6179 H 0 0 0 0 0 0 0 0 0 0 0 0
3.9139 9.2585 0.3229 H 0 0 0 0 0 0 0 0 0 0 0 0
5.7875 10.7603 -0.0450 H 0 0 0 0 0 0 0 0 0 0 0 0
8.1091 9.9544 0.2062 H 0 0 0 0 0 0 0 0 0 0 0 0
8.5238 7.5892 0.8390 H 0 0 0 0 0 0 0 0 0 0 0 0
9.1099 -3.8483 0.6805 H 0 0 0 0 0 0 0 0 0 0 0 0
10.6258 -5.7394 0.5171 H 0 0 0 0 0 0 0 0 0 0 0 0
9.8184 -8.0351 0.9434 H 0 0 0 0 0 0 0 0 0 0 0 0
7.4339 -8.4063 1.5298 H 0 0 0 0 0 0 0 0 0 0 0 0
7.7350 -2.4582 0.8831 H 0 0 0 0 0 0 0 0 0 0 0 0
-3.8398 -3.5582 2.5052 H 0 0 0 0 0 0 0 0 0 0 0 0
-2.5412 -4.7412 2.3414 H 0 0 0 0 0 0 0 0 0 0 0 0
-2.5597 -7.8740 0.5339 H 0 0 0 0 0 0 0 0 0 0 0 0
-3.9426 -9.2353 0.2371 H 0 0 0 0 0 0 0 0 0 0 0 0
-5.8337 -10.7210 -0.1057 H 0 0 0 0 0 0 0 0 0 0 0 0
-8.1433 -9.9074 0.2203 H 0 0 0 0 0 0 0 0 0 0 0 0
-8.5278 -7.5505 0.9015 H 0 0 0 0 0 0 0 0 0 0 0 0
-7.7524 2.4752 0.8397 H 0 0 0 0 0 0 0 0 0 0 0 0
-9.1150 3.8745 0.6325 H 0 0 0 0 0 0 0 0 0 0 0 0
-10.6161 5.7767 0.4637 H 0 0 0 0 0 0 0 0 0 0 0 0
-9.8000 8.0635 0.9208 H 0 0 0 0 0 0 0 0 0 0 0 0
-7.4222 8.4139 1.5452 H 0 0 0 0 0 0 0 0 0 0 0 0
3.9944 -0.8503 -5.1079 C 0 0 0 0 0 0 0 0 0 0 0 0
5.8468 -0.3910 -3.4959 C 0 0 0 0 0 0 0 0 0 0 0 0
-0.1416 4.8019 -5.1098 C 0 0 0 0 0 0 0 0 0 0 0 0
1.7489 4.7092 -3.4761 C 0 0 0 0 0 0 0 0 0 0 0 0
```

```
  -4.7017   -0.0090   -5.1592 C   0  0  0  0  0  0  0  0  0  0  0  0
  -4.6561    1.8054   -3.4388 C   0  0  0  0  0  0  0  0  0  0  0  0
   0.1131   -4.7454   -5.1795 C   0  0  0  0  0  0  0  0  0  0  0  0
  -1.7580   -4.6860   -3.5221 C   0  0  0  0  0  0  0  0  0  0  0  0
   4.1430   -1.6404   -3.1191 H   0  0  0  0  0  0  0  0  0  0  0  0
   0.1425   -5.6360   -3.2325 H   0  0  0  0  0  0  0  0  0  0  0  0
  -5.6128   -0.1012   -3.2234 H   0  0  0  0  0  0  0  0  0  0  0  0
  -0.1423    5.6668   -3.1510 H   0  0  0  0  0  0  0  0  0  0  0  0
   2.9461   -1.1161   -5.2236 H   0  0  0  0  0  0  0  0  0  0  0  0
   4.1736    0.0816   -5.6409 H   0  0  0  0  0  0  0  0  0  0  0  0
   4.5964   -1.6299   -5.5695 H   0  0  0  0  0  0  0  0  0  0  0  0
   6.0870    0.5493   -3.9888 H   0  0  0  0  0  0  0  0  0  0  0  0
   6.1419   -0.3170   -2.4534 H   0  0  0  0  0  0  0  0  0  0  0  0
   6.4353   -1.1806   -3.9585 H   0  0  0  0  0  0  0  0  0  0  0  0
   0.1944    3.8984   -5.6148 H   0  0  0  0  0  0  0  0  0  0  0  0
  -1.2190    4.8864   -5.2360 H   0  0  0  0  0  0  0  0  0  0  0  0
   0.3271    5.6567   -5.5923 H   0  0  0  0  0  0  0  0  0  0  0  0
   2.0368    4.7034   -2.4295 H   0  0  0  0  0  0  0  0  0  0  0  0
   2.1475    3.8102   -3.9411 H   0  0  0  0  0  0  0  0  0  0  0  0
   2.2009    5.5744   -3.9571 H   0  0  0  0  0  0  0  0  0  0  0  0
  -3.7902    0.3569   -5.6277 H   0  0  0  0  0  0  0  0  0  0  0  0
  -4.7735   -1.0802   -5.3355 H   0  0  0  0  0  0  0  0  0  0  0  0
  -5.5496    0.4742   -5.6397 H   0  0  0  0  0  0  0  0  0  0  0  0
  -4.6784    2.0455   -2.3804 H   0  0  0  0  0  0  0  0  0  0  0  0
  -3.7474    2.2275   -3.8624 H   0  0  0  0  0  0  0  0  0  0  0  0
  -5.5106    2.2762   -3.9206 H   0  0  0  0  0  0  0  0  0  0  0  0
  -0.2385   -3.8397   -5.6695 H   0  0  0  0  0  0  0  0  0  0  0  0
   1.1896   -4.8171   -5.3194 H   0  0  0  0  0  0  0  0  0  0  0  0
  -0.3528   -5.5993   -5.6664 H   0  0  0  0  0  0  0  0  0  0  0  0
  -2.0336   -4.6932   -2.4722 H   0  0  0  0  0  0  0  0  0  0  0  0
  -2.1675   -3.7847   -3.9731 H   0  0  0  0  0  0  0  0  0  0  0  0
  -2.2105   -5.5485   -4.0072 H   0  0  0  0  0  0  0  0  0  0  0  0
   2.0412    6.2701    0.9976 H   0  0  0  0  0  0  0  0  0  0  0  0
   6.1109   -1.9319    1.1737 H   0  0  0  0  0  0  0  0  0  0  0  0
  -2.0342   -6.2745    0.9315 H   0  0  0  0  0  0  0  0  0  0  0  0
  -6.1379    1.9330    1.1489 H   0  0  0  0  0  0  0  0  0  0  0  0
  1  2  1  0  0  0  0
  2  3  1  0  0  0  0
  2  7  1  0  0  0  0
  3 68  1  0  0  0  0
  4  8  1  0  0  0  0
  4  3  1  0  0  0  0
  5  4  1  0  0  0  0
  6  5  1  0  0  0  0
  6  1  1  0  0  0  0
  8 69  1  0  0  0  0
  9  6  1  0  0  0  0
 10 59  1  0  0  0  0
 10 11  1  0  0  0  0
 10  5  1  0  0  0  0
 11 12  1  0  0  0  0
 12 13  1  0  0  0  0
 12 17  1  0  0  0  0
 13 70  1  0  0  0  0
 14 67  1  0  0  0  0
 14 13  1  0  0  0  0
 15 14  1  0  0  0  0
 16 15  1  0  0  0  0
 16 11  1  0  0  0  0
 18 16  1  0  0  0  0
 19 42  1  0  0  0  0
 19 15  1  0  0  0  0
 19 20  1  0  0  0  0
 20 21  1  0  0  0  0
 21 26  1  0  0  0  0
 21 22  1  0  0  0  0
 22 96  1  0  0  0  0
 23 22  1  0  0  0  0
 23 27  1  0  0  0  0
 24 23  1  0  0  0  0
 25 20  1  0  0  0  0
 25 24  1  0  0  0  0
 27 63  1  0  0  0  0
 28 25  1  0  0  0  0
 29 44  1  0  0  0  0
 29 24  1  0  0  0  0
 29 30  1  0  0  0  0
```

30 31 1 0 0 0 0
31 32 1 0 0 0 0
31 36 1 0 0 0 0
32 60 1 0 0 0 0
33 37 1 0 0 0 0
33 32 1 0 0 0 0
34 33 1 0 0 0 0
35 30 1 0 0 0 0
35 34 1 0 0 0 0
37 65 1 0 0 0 0
38 35 1 0 0 0 0
39 46 1 0 0 0 0
39 1 1 0 0 0 0
39 34 1 0 0 0 0
40 10 1 0 0 0 0
41 19 1 0 0 0 0
43 29 1 0 0 0 0
45 39 1 0 0 0 0
47 40 1 0 0 0 0
48 40 1 0 0 0 0
49150 1 0 0 0 0
49157 1 0 0 0 0
49 40 1 0 0 0 0
50 41 1 0 0 0 0
51 41 1 0 0 0 0
52156 1 0 0 0 0
52158 1 0 0 0 0
52 41 1 0 0 0 0
53 43 1 0 0 0 0
54 43 1 0 0 0 0
55154 1 0 0 0 0
55159 1 0 0 0 0
55 43 1 0 0 0 0
56 45 1 0 0 0 0
57 45 1 0 0 0 0
58152 1 0 0 0 0
58160 1 0 0 0 0
58 45 1 0 0 0 0
60122 1 0 0 0 0
60121 1 0 0 0 0
61 17 1 0 0 0 0
62 26 1 0 0 0 0
64 36 1 0 0 0 0
66 7 1 0 0 0 0
67 71 1 0 0 0 0
68124 1 0 0 0 0
68123 1 0 0 0 0
70126 1 0 0 0 0
70125 1 0 0 0 0
72 77 1 0 0 0 0
72 68 1 0 0 0 0
73 72 1 0 0 0 0
74 75 1 0 0 0 0
74 73 1 0 0 0 0
75 76 1 0 0 0 0
76 77 1 0 0 0 0
77 78 1 0 0 0 0
79185 1 0 0 0 0
79 73 1 0 0 0 0
80 74 1 0 0 0 0
81 80 1 0 0 0 0
81 82 1 0 0 0 0
82 83 1 0 0 0 0
83131 1 0 0 0 0
83 75 1 0 0 0 0
84 89 1 0 0 0 0
84 70 1 0 0 0 0
85 84 1 0 0 0 0
86 87 1 0 0 0 0
86 85 1 0 0 0 0
87 88 1 0 0 0 0
88 89 1 0 0 0 0
89 90 1 0 0 0 0
91 86 1 0 0 0 0
92 93 1 0 0 0 0
92 91 1 0 0 0 0
93 94 1 0 0 0 0

94135 1 0 0 0 0
94 87 1 0 0 0 0
95 85 1 0 0 0 0
95186 1 0 0 0 0
96138 1 0 0 0 0
96137 1 0 0 0 0
97100 1 0 0 0 0
97 96 1 0 0 0 0
98 99 1 0 0 0 0
99100 1 0 0 0 0
100101 1 0 0 0 0
102 97 1 0 0 0 0
103102 1 0 0 0 0
103 98 1 0 0 0 0
104187 1 0 0 0 0
104102 1 0 0 0 0
105103 1 0 0 0 0
106105 1 0 0 0 0
106107 1 0 0 0 0
107108 1 0 0 0 0
108 98 1 0 0 0 0
108143 1 0 0 0 0
109112 1 0 0 0 0
109 60 1 0 0 0 0
110111 1 0 0 0 0
111112 1 0 0 0 0
112113 1 0 0 0 0
114109 1 0 0 0 0
115110 1 0 0 0 0
115114 1 0 0 0 0
116114 1 0 0 0 0
116188 1 0 0 0 0
117115 1 0 0 0 0
118119 1 0 0 0 0
118117 1 0 0 0 0
119120 1 0 0 0 0
120148 1 0 0 0 0
120110 1 0 0 0 0
127 79 1 0 0 0 0
128 80 1 0 0 0 0
129 81 1 0 0 0 0
130 82 1 0 0 0 0
132 91 1 0 0 0 0
133 92 1 0 0 0 0
134 93 1 0 0 0 0
136 95 1 0 0 0 0
139104 1 0 0 0 0
140105 1 0 0 0 0
141106 1 0 0 0 0
142107 1 0 0 0 0
144116 1 0 0 0 0
145117 1 0 0 0 0
146118 1 0 0 0 0
147119 1 0 0 0 0
149 49 1 0 0 0 0
150165 1 0 0 0 0
151 58 1 0 0 0 0
152170 1 0 0 0 0
153 55 1 0 0 0 0
154176 1 0 0 0 0
155 52 1 0 0 0 0
156182 1 0 0 0 0
161149 1 0 0 0 0
162149 1 0 0 0 0
163149 1 0 0 0 0
164150 1 0 0 0 0
166150 1 0 0 0 0
167151 1 0 0 0 0
168151 1 0 0 0 0
169151 1 0 0 0 0
171152 1 0 0 0 0
172152 1 0 0 0 0
173153 1 0 0 0 0
174153 1 0 0 0 0
175153 1 0 0 0 0
177154 1 0 0 0 0
178154 1 0 0 0 0

179155 1 0 0 0 0
180155 1 0 0 0 0
181155 1 0 0 0 0
183156 1 0 0 0 0
184156 1 0 0 0 0
M END

Optimizing the geometry of the compound **3** by **DFT/B3LYP/6-311G(d,p)**

Crown-*in*-Gas phase

E = -4675.56969264 H

188

C	-2.66860	-1.55980	-2.86110
C	-1.53870	-1.63970	-3.69060
C	-0.95560	-0.49610	-4.25650
C	-1.52320	0.75220	-3.95660
C	-2.63830	0.88010	-3.11620
C	-3.17060	-0.29200	-2.58160
O	-1.04390	-2.89600	-3.96840
O	-0.96790	1.85080	-4.57160
C	-3.23890	2.25720	-2.81660
C	-2.63210	2.86450	-1.54750
C	-1.50380	3.69630	-1.62270
C	-0.93070	4.26900	-0.47780
C	-1.50750	3.97390	0.76790
C	-2.62700	3.13920	0.88990
C	-3.15150	2.59900	-0.28340
O	-0.99760	3.96460	-2.87670
C	-3.24930	2.84820	2.26020
C	-2.64850	1.57090	2.85960
C	-1.51780	1.63950	3.68920
C	-0.94630	0.49090	4.25620
C	-1.52600	-0.75260	3.95690
C	-2.64210	-0.86890	3.11720
C	-3.16310	0.30800	2.58090
O	-1.01190	2.89160	3.96720
O	-0.98430	-1.85630	4.57440
C	-3.27090	-2.23660	2.82550
C	-2.66930	-2.84640	1.55340
C	-1.54390	-3.68300	1.62910
C	-0.97390	-4.26100	0.48520
C	-1.54840	-3.96490	-0.76140
C	-2.65910	-3.11940	-0.88480
C	-3.17930	-2.57250	0.28760
O	-1.04180	-3.95810	2.88330
O	-1.00660	-4.58920	-1.86120
C	-3.28100	-2.82830	-2.25560
C	-4.78430	2.24680	-2.82630
C	-4.79950	2.89300	2.22340
C	-4.82120	-2.19000	2.86420
C	-4.83140	-2.85750	-2.21490
C	-5.45570	3.63290	-2.75490
C	-5.48430	3.31820	3.53960
C	-5.51740	-3.49950	3.29200
C	-5.52660	-3.27030	-3.52970
C	0.17180	-5.26250	0.58500
O	-0.95990	4.59120	1.86900
C	0.20180	-0.59580	-5.24450
C	0.22210	5.26230	-0.57610
C	1.62690	-0.58560	-4.70290
C	2.37920	0.58050	-4.57630
C	3.80340	0.46800	-4.26790
C	4.31730	-0.79970	-3.97380
O	3.51690	-1.90170	-3.94830
C	2.18920	-1.83650	-4.32450
O	1.59360	-2.91860	-4.27370
N	1.85840	1.80140	-4.79770

C	4.70190	1.54880	-4.30950
C	6.05040	1.36110	-4.05700
C	6.53410	0.08630	-3.74170
C	5.67040	-0.99580	-3.69880
C	1.65110	4.73140	-0.55340
C	2.39630	4.61800	0.61850
C	3.82490	4.32670	0.51740
C	4.35080	4.03190	-0.74530
O	3.55750	3.98660	-1.85170
C	2.22500	4.34780	-1.79770
O	1.63570	4.27690	-2.88200
C	4.71610	4.38880	1.60320
C	6.06950	4.15540	1.42550
C	6.56570	3.83990	0.15550
C	5.70930	3.77690	-0.93140
N	1.86370	4.83770	1.83480
C	0.20800	0.58110	5.24870
C	1.63490	0.56050	4.71220
C	4.32910	0.75430	3.99180
O	3.53770	1.86290	3.96620
C	2.20860	1.80780	4.33930
O	1.62150	2.89450	4.28930
C	2.37770	-0.61150	4.58440
C	3.80390	-0.50980	4.28210
N	1.84580	-1.82880	4.79890
C	4.69360	-1.59780	4.32550
C	6.04440	-1.42070	4.07770
C	6.53930	-0.14950	3.76520
C	5.68450	0.93970	3.72110
C	1.60420	-4.74110	0.56150
C	4.30640	-4.05160	0.75170
O	3.51410	-4.00480	1.85890
C	2.18020	-4.36080	1.80560
O	1.59200	-4.29140	2.89080
C	2.34920	-4.63130	-0.61090
C	3.77890	-4.34500	-0.51050
N	1.81530	-4.85020	-1.82640
C	4.66910	-4.40910	-1.59710
C	6.02310	-4.17840	-1.42040
C	6.52090	-3.86360	-0.15090
C	5.66550	-3.79910	0.93680
C	-6.98040	3.47170	-2.67150
C	-5.07380	4.53970	-3.93440
C	-7.02500	-3.49430	-3.27620
C	-5.32360	-2.28500	-4.68920
C	-7.01700	-3.24460	3.50490
C	-5.30790	-4.67260	2.32460
C	-6.98400	3.54420	3.29610
C	-5.27450	2.34120	4.70470
H	-4.02870	-0.21180	-1.92710
H	-0.08410	-2.87020	-4.21120
H	-1.10410	2.65540	-4.02430
H	-2.95730	2.90770	-3.64410
H	-4.01770	1.95320	-0.20790
H	-0.03750	4.20580	-2.84510
H	-2.96510	3.67120	2.91920
H	-4.02610	0.23330	1.93130
H	-0.05240	2.85840	4.21040
H	-1.13200	-2.65970	4.02780
H	-2.99490	-2.89420	3.65240
H	-4.03750	-1.91730	0.20710
H	-0.08340	-4.20560	2.85220
H	-1.14990	-4.04430	-2.66630
H	-3.00690	-3.65750	-2.91100
H	-5.10400	1.74660	-3.74840
H	-5.17250	1.63530	-2.00580
H	-5.08260	3.62410	1.46000
H	-5.22080	1.93730	1.89310
H	-5.10130	-1.42210	3.59180
H	-5.23710	-1.85990	1.90600
H	-5.11830	-3.58950	-1.45360
H	-5.24220	-1.89940	-1.87820
H	-5.11990	4.12390	-1.83400
H	-5.04750	4.28350	3.83040
H	-5.08990	-3.78670	4.26270
H	-5.09490	-4.23480	-3.83090
H	0.05040	-5.83160	1.51040

H	0.05790	-5.98680	-0.22360
H	-1.10410	4.04460	2.67280
H	0.08200	-1.51610	-5.82200
H	0.10260	0.21910	-5.96390
H	0.10430	5.83320	-1.50080
H	0.11330	5.98650	0.23340
H	2.30460	2.61780	-4.40020
H	0.84360	1.87740	-4.85280
H	4.34360	2.53870	-4.56040
H	6.73190	2.20130	-4.11580
H	7.58860	-0.06080	-3.53950
H	6.01340	-1.99390	-3.46200
H	4.34810	4.64190	2.58890
H	6.74480	4.22980	2.26960
H	7.62420	3.65410	0.01580
H	6.06270	3.54140	-1.92610
H	2.30660	4.44140	2.65350
H	0.84760	4.87940	1.90250
H	0.09330	1.50180	5.82680
H	0.09970	-0.23350	5.96710
H	2.28560	-2.64680	4.39780
H	0.83000	-1.89600	4.84870
H	4.32650	-2.58490	4.57460
H	6.71900	-2.26640	4.13810
H	7.59560	-0.01060	3.56640
H	6.03670	1.93520	3.48700
H	2.25960	-4.45950	-2.64700
H	0.79900	-4.88970	-1.89380
H	4.29970	-4.66070	-2.58270
H	6.69770	-4.25420	-2.26490
H	7.57980	-3.67930	-0.01210
H	6.01990	-3.56310	1.93100
H	-7.27310	2.86480	-1.80940
H	-7.37380	2.98460	-3.57070
H	-7.47460	4.44320	-2.57860
H	-5.34120	4.07490	-4.89030
H	-4.00440	4.76220	-3.95560
H	-5.60300	5.49520	-3.87230
H	-7.51400	-2.56250	-2.97090
H	-7.19190	-4.23270	-2.48630
H	-7.52820	-3.84960	-4.18000
H	-4.27160	-2.16700	-4.95310
H	-5.71240	-1.29390	-4.43330
H	-5.85600	-2.63200	-5.58010
H	-7.50150	-2.95310	2.56640
H	-7.18850	-2.44460	4.23120
H	-7.52130	-4.14370	3.87070
H	-4.25500	-4.93610	2.21470
H	-5.69350	-4.43190	1.32840
H	-5.84010	-5.55950	2.68200
H	-7.47780	2.61170	3.00100
H	-7.15520	4.27750	2.50250
H	-7.47940	3.90710	4.20120
H	-4.22050	2.21930	4.95870
H	-5.67200	1.35060	4.46040
H	-5.79580	2.69790	5.59830

Crown-*in* $CHCl_3$
E = -4675.587645 H

188

C	2.66210	-2.58050	-1.99820
C	1.53580	-3.39270	-2.20610
C	0.96030	-4.13880	-1.16610
C	1.52820	-4.03460	0.11400
C	2.64180	-3.22060	0.37070
C	3.16930	-2.50470	-0.70370
O	1.04070	-3.47010	-3.48970
O	0.97480	-4.81380	1.10480
C	3.24840	-3.14030	1.77580
C	2.64210	-1.98360	2.57710
C	1.51550	-2.19210	3.38880
C	0.94110	-1.15330	4.13700
C	1.51430	0.12560	4.04030
C	2.63480	0.37970	3.23580

C	3.16140	-0.69280	2.51620
O	1.01650	-3.47450	3.45900
C	3.25960	1.77820	3.16630
C	2.65740	2.57430	2.00140
C	1.53140	3.38880	2.20560
C	0.96060	4.13620	1.16460
C	1.53190	4.03210	-0.11430
C	2.64470	3.21690	-0.36700
C	3.16830	2.49850	0.70820
O	1.03330	3.46840	3.48810
O	0.98460	4.81510	-1.10520
C	3.26960	3.14280	-1.76590
C	2.66310	1.98280	-2.56610
C	1.53660	2.19320	-3.37860
C	0.96180	1.15700	-4.12970
C	1.53100	-0.12350	-4.03320
C	2.64670	-0.38130	-3.22410
C	3.17300	0.68880	-2.50010
O	1.04190	3.47730	-3.45300
O	0.97820	-1.11060	-4.81770
C	3.26860	-1.78140	-3.15820
C	4.79410	-3.14110	1.75440
C	4.81030	1.73250	3.19920
C	4.82050	3.16880	-1.71890
C	4.81940	-1.73840	-3.18670
C	5.47680	-3.28350	3.12970
C	5.49630	2.95610	3.84260
C	5.51090	3.81040	-2.94100
C	5.50580	-2.96410	-3.82570
C	-0.18350	1.41930	-5.10230
O	0.96120	1.11520	4.82090
C	-0.18670	-5.11060	-1.42400
C	-0.20870	-1.41180	5.10520
C	-1.61730	-4.58930	-1.35340
C	-2.38570	-4.64790	-0.19160
C	-3.81280	-4.34640	-0.27610
C	-4.31840	-3.88860	-1.49810
O	-3.50490	-3.69610	-2.57640
C	-2.17460	-4.05190	-2.54550
O	-1.56710	-3.84400	-3.60470
N	-1.86860	-5.04090	0.98680
C	-4.72300	-4.54450	0.77830
C	-6.07290	-4.28540	0.60930
C	-6.54830	-3.81180	-0.61940
C	-5.67350	-3.61240	-1.67520
C	-1.63800	-1.33660	4.58090
C	-2.40380	-0.17330	4.64230
C	-3.83070	-0.25340	4.33830
C	-4.33800	-1.47240	3.87440
O	-3.52670	-2.55190	3.67990
C	-2.19730	-2.52560	4.03850
O	-1.59230	-3.58610	3.82960
C	-4.73890	0.80210	4.53900
C	-6.08860	0.63730	4.27620
C	-6.56570	-0.58810	3.79590
C	-5.69290	-1.64520	3.59410
N	-1.88450	1.00250	5.04020
C	-0.18300	5.11270	1.42030
C	-1.61550	4.59770	1.34640
C	-4.31980	3.90870	1.48430
O	-3.50980	3.71230	2.56450
C	-2.17780	4.06200	2.53690
O	-1.57350	3.85100	3.59730
C	-2.38070	4.65990	0.18280
C	-3.80930	4.36460	0.26360
N	-1.85870	5.05100	-0.99410
C	-4.71610	4.56720	-0.79280
C	-6.06760	4.31410	-0.62710
C	-6.54800	3.84210	0.60020
C	-5.67660	3.63850	1.65810
C	-1.61490	1.34750	-4.58380
C	-4.31690	1.49020	-3.88660
O	-3.50350	2.56770	-3.68920
C	-2.17300	2.53790	-4.04370
O	-1.56570	3.59670	-3.83280
C	-2.38350	0.18620	-4.64820
C	-3.81120	0.27000	-4.34920

N	-1.86570	-0.99090	-5.04430
C	-4.72160	-0.78300	-4.55360
C	-6.07170	-0.61470	-4.29520
C	-6.54710	0.61170	-3.81570
C	-5.67220	1.66630	-3.61050
C	6.99890	-3.15490	2.97160
C	5.11930	-4.59950	3.83630
C	7.00790	-2.68930	-3.99310
C	5.28630	-4.27830	-3.06370
C	7.01250	3.97530	-2.66200
C	5.29380	3.05300	-4.25820
C	6.99760	2.67970	4.01400
C	5.28020	4.27210	3.08280
H	4.02780	-1.87100	-0.52370
H	0.08110	-3.72720	-3.50740
H	1.10410	-4.39300	1.98370
H	2.96920	-4.06050	2.28860
H	4.02860	-0.51400	1.89260
H	0.05600	-3.49130	3.71300
H	2.97770	2.29760	4.08440
H	4.02950	1.86750	0.52810
H	0.07420	3.72760	3.50310
H	1.11870	4.39620	-1.98450
H	2.99300	4.06310	-2.28430
H	4.03580	0.50380	-1.87270
H	0.08230	3.49610	-3.71020
H	1.11400	-1.99200	-4.40410
H	2.98860	-2.29630	-4.07930
H	5.11550	-3.97290	1.11600
H	5.17310	-2.23310	1.27560
H	5.09680	0.85540	3.78720
H	5.22760	1.57260	2.19940
H	5.10820	3.75510	-0.84120
H	5.23350	2.16720	-1.55880
H	5.10850	-0.86270	-3.77540
H	5.23420	-1.57730	-2.18600
H	5.13560	-2.45780	3.76520
H	5.06490	3.07510	4.84600
H	5.08380	4.81570	-3.05960
H	5.07730	-3.08460	-4.83010
H	-0.04500	2.41400	-5.53350
H	-0.08550	0.72180	-5.93580
H	1.10000	1.99540	4.40560
H	-0.04950	-5.54570	-2.41710
H	-0.08970	-5.94160	-0.72340
H	-0.07440	-2.40670	5.53740
H	-0.11240	-0.71410	5.93870
H	-2.34590	-4.81810	1.84910
H	-0.85480	-5.08290	1.07190
H	-4.37670	-4.92360	1.73080
H	-6.76200	-4.46060	1.42630
H	-7.60410	-3.60740	-0.75180
H	-6.01300	-3.25440	-2.63780
H	-4.39130	1.75190	4.92360
H	-6.77630	1.45510	4.45330
H	-7.62120	-0.71720	3.58820
H	-6.03360	-2.60540	3.23090
H	-2.36010	1.86660	4.82130
H	-0.87050	1.08570	5.08300
H	-0.04610	5.54710	2.41390
H	-0.08090	5.94340	0.72010
H	-2.33430	4.83020	-1.85780
H	-0.84450	5.08890	-1.07660
H	-4.36570	4.94530	-1.74420
H	-6.75390	4.49270	-1.44570
H	-7.60490	3.64240	0.73010
H	-6.02000	3.28170	2.61980
H	-2.34470	-1.85380	-4.82800
H	-0.85180	-1.07690	-5.08380
H	-4.37510	-1.73360	-4.93720
H	-6.76090	-1.43050	-4.47520
H	-7.60290	0.74340	-3.61150
H	-6.01150	2.62720	-3.24760
H	7.27330	-2.20260	2.50750
H	7.39620	-3.95940	2.34270
H	7.50240	-3.21210	3.94100
H	5.39330	-5.46170	3.21770

H	4.05260	-4.67220	4.06090
H	5.65820	-4.68600	4.78430
H	7.49120	-2.55600	-3.01880
H	7.18670	-1.78460	-4.58160
H	7.50590	-3.52230	-4.49790
H	4.23030	-4.54500	-2.99680
H	5.67550	-4.20810	-2.04250
H	5.80840	-5.09950	-3.56420
H	7.49430	3.00000	-2.53010
H	7.18950	4.56100	-1.75510
H	7.51310	4.48180	-3.49230
H	4.23830	2.98630	-4.52690
H	5.68370	2.03190	-4.19110
H	5.81700	3.55670	-5.07690
H	7.48370	2.54780	3.04090
H	7.17370	1.77340	4.60100
H	7.49480	3.51110	4.52210
H	4.22440	4.53880	3.01140
H	5.67450	4.20430	2.06330
H	5.79990	5.09230	3.58760

Crown-*in* DMSO

E = -4675.59688221 H

188

C	2.66690	2.75530	1.75300
C	1.54640	3.59150	1.88650
C	0.97130	4.23880	0.78190
C	1.53290	4.00870	-0.48490
C	2.64530	3.17180	-0.66590
C	3.17370	2.55860	0.47060
O	1.05900	3.79430	3.15870
O	0.97580	4.69010	-1.54320
C	3.25140	2.96200	-2.05820
C	2.64160	1.73770	-2.74840
C	1.51400	1.87280	-3.57470
C	0.93220	0.76910	-4.21730
C	1.50110	-0.49740	-4.00130
C	2.62820	-0.67710	-3.18510
C	3.16060	0.45720	-2.57150
O	1.02320	3.14540	-3.76740
C	3.25570	-2.06300	-2.99220
C	2.65700	-2.75020	-1.75840
C	1.53640	-3.58780	-1.88680
C	0.96700	-4.23610	-0.78040
C	1.53390	-4.00680	0.48460
C	2.64610	-3.16990	0.66010
C	3.16910	-2.55410	-0.47810
O	1.04470	-3.79290	-3.15720
O	0.98390	-4.69200	1.54390
C	3.27460	-2.96710	2.04510
C	2.66700	-1.73910	2.73520
C	1.54020	-1.87540	3.56350
C	0.95860	-0.77390	4.20940
C	1.52380	0.49410	3.99300
C	2.64600	0.67640	3.17150
C	3.17780	-0.45620	2.55320
O	1.05400	-3.14910	3.76210
O	0.96010	1.54870	4.67480
C	3.27080	2.06420	2.98150
C	4.79760	2.96220	-2.03930
C	4.80650	-2.01910	-3.03220
C	4.82550	-2.99460	1.99770
C	4.82170	2.02290	3.01570
C	5.47800	2.97490	-3.42300
C	5.49160	-3.29540	-3.56470
C	5.51740	-3.52610	3.27090
C	5.50710	3.29990	3.54640
C	-0.18800	-0.94700	5.20020

O	0.93640	-1.55390	-4.67880
C	-0.16080	5.24660	0.95150
C	-0.21940	0.93920	-5.20260
C	-1.59930	4.74350	0.94920
C	-2.38560	4.71000	-0.20180
C	-3.81610	4.44300	-0.06930
C	-4.31030	4.11720	1.19910
O	-3.48290	4.01600	2.28030
C	-2.14850	4.33870	2.19550
O	-1.52810	4.22740	3.26280
N	-1.87780	4.98190	-1.41790
C	-4.73980	4.54690	-1.12590
C	-6.09030	4.32450	-0.91370
C	-6.55410	3.98670	0.36360
C	-5.66650	3.88360	1.42280
C	-1.64550	0.93780	-4.66540
C	-2.42860	-0.21470	-4.60700
C	-3.85010	-0.08450	-4.29450
C	-4.33670	1.18450	-3.95900
O	-3.51070	2.26940	-3.89440
C	-2.18650	2.18580	-4.25500
O	-1.56640	3.25610	-4.17200
C	-4.77330	-1.14490	-4.35590
C	-6.11540	-0.93520	-4.08570
C	-6.57100	0.34270	-3.73960
C	-5.68400	1.40540	-3.67620
N	-1.92550	-1.42950	-4.89170
C	-0.16250	-5.24750	-0.94630
C	-1.60250	-4.74920	-0.93910
C	-4.31660	-4.13300	-1.17890
O	-3.49370	-4.02870	-2.26330
C	-2.15790	-4.34670	-2.18340
O	-1.54180	-4.23330	-3.25290
C	-2.38440	-4.71740	0.21500
C	-3.81630	-4.45630	0.08780
N	-1.87050	-4.98600	1.42920
C	-4.73570	-4.56390	1.14780
C	-6.08790	-4.34730	0.94050
C	-6.55780	-4.01160	-0.33510
C	-5.67460	-3.90510	-1.39760
C	-1.61650	-0.94820	4.66990
C	-4.31040	-1.20040	3.97520
O	-3.48230	-2.28360	3.90670
C	-2.15680	-2.19720	4.26180
O	-1.53480	-3.26620	4.17630
C	-2.40250	0.20260	4.61580
C	-3.82500	0.06940	4.30960
N	-1.90090	1.41830	4.89930
C	-4.75040	1.12760	4.37580
C	-6.09310	0.91520	4.11110
C	-6.54740	-0.36340	3.76580
C	-5.65830	-1.42400	3.69790
C	7.00110	2.87210	-3.25460
C	5.11260	4.21320	-4.25470
C	7.01040	3.04280	3.73010
C	5.28170	4.54430	2.67640
C	7.02150	-3.69790	3.01020
C	5.28790	-2.66430	4.52000
C	6.99410	-3.03690	-3.75300
C	5.27000	-4.53990	-2.69400
H	4.03360	1.91270	0.35090
H	0.10120	4.06570	3.15870
H	1.10080	4.18820	-2.37950
H	2.97160	3.83180	-2.65210
H	4.03390	0.33650	-1.94250
H	0.06250	3.14770	-4.02850
H	2.97110	-2.66300	-3.85880
H	4.03230	-1.91130	-0.35870
H	0.08760	-4.06630	-3.15350
H	1.11710	-4.19300	2.38080
H	2.99910	-3.83610	2.64610
H	4.04690	-0.33020	1.92010
H	0.09460	-3.15300	4.02750
H	1.10470	2.39050	4.18820
H	2.98870	2.65990	3.85190
H	5.12160	3.85020	-1.48310
H	5.17700	2.10290	-1.47840

H	5.09200	-1.19840	-3.69700
H	5.22570	-1.76980	-2.05210
H	5.11460	-3.65460	1.17460
H	5.23700	-2.01070	1.75030
H	5.11090	1.20230	3.67890
H	5.23790	1.77490	2.03390
H	5.14300	2.08870	-3.97470
H	5.06380	-3.50040	-4.55560
H	5.09980	-4.52240	3.47110
H	5.08200	3.50430	4.53860
H	-0.03960	-1.88950	5.73330
H	-0.10270	-0.16550	5.95680
H	1.08450	-2.39460	-4.19090
H	-0.00580	5.77730	1.89430
H	-0.06060	6.00180	0.17050
H	-0.07540	1.88180	-5.73660
H	-0.13630	0.15760	-5.95930
H	-2.38280	4.71610	-2.25070
H	-0.86570	4.99110	-1.52470
H	-4.40520	4.82360	-2.11690
H	-6.78830	4.42200	-1.73580
H	-7.61030	3.81310	0.53040
H	-5.99810	3.63480	2.42200
H	-4.44560	-2.13750	-4.63510
H	-6.81400	-1.76000	-4.15080
H	-7.62040	0.50690	-3.52680
H	-6.00890	2.40470	-3.41910
H	-2.42520	-2.26540	-4.62650
H	-0.91410	-1.53630	-4.92850
H	-0.00900	-5.77780	-1.88960
H	-0.05740	-6.00230	-0.16550
H	-2.37300	-4.72130	2.26390
H	-0.85790	-4.99140	1.53220
H	-4.39630	-4.83950	2.13750
H	-6.78250	-4.44760	1.76520
H	-7.61540	-3.84250	-0.49800
H	-6.01090	-3.65790	-2.39560
H	-2.40380	2.25320	4.63730
H	-0.88960	1.52750	4.93190
H	-4.42360	2.12080	4.65440
H	-6.79320	1.73840	4.18000
H	-7.59730	-0.52970	3.55740
H	-5.98210	-2.42380	3.44140
H	7.28180	1.97060	-2.70190
H	7.39230	3.73610	-2.70590
H	7.50320	2.84000	-4.22600
H	5.38690	5.13330	-3.72630
H	4.04440	4.26050	-4.47990
H	5.64700	4.20620	-5.20920
H	7.48910	2.82820	2.76820
H	7.19230	2.19060	4.39150
H	7.50860	3.91590	4.16130
H	4.22460	4.80270	2.59330
H	5.66600	4.38810	1.66290
H	5.80480	5.40580	3.10290
H	7.49320	-2.73140	2.80070
H	7.20660	-4.35290	2.15370
H	7.52410	-4.13080	3.87990
H	4.23040	-2.58870	4.77940
H	5.66630	-1.64780	4.36920
H	5.81380	-3.09250	5.37900
H	7.47550	-2.82160	-2.79260
H	7.17330	-2.18470	-4.41520
H	7.49180	-3.90970	-4.18550
H	4.21320	-4.79860	-2.60680
H	5.65810	-4.38350	-1.68190
H	5.79160	-5.40120	-3.12240

Crown-*out* Gas phase

E = -4675.55263832 H

188

C	-2.37930	2.23970	1.01370
C	-3.19480	2.51960	-0.09420
C	-4.05180	1.55620	-0.64930
C	-4.06250	0.27390	-0.08210
C	-3.24700	-0.05680	1.01070
C	-2.41920	0.94040	1.52070
O	-3.20390	3.76440	-0.68400
O	-4.82670	-0.73290	-0.62160
H	-1.78870	0.69940	2.36720
C	-3.29200	-1.47180	1.59350
C	-2.21280	-2.37470	0.98760
C	-2.49080	-3.18460	-0.12440
C	-1.52880	-4.04440	-0.67720
C	-0.25040	-4.06600	-0.10070
C	0.07570	-3.26120	1.00100
C	-0.91990	-2.43050	1.50950
O	-3.73170	-3.18150	-0.72280
H	-0.68320	-1.81280	2.36760
C	1.48170	-3.31800	1.60640
C	2.38340	-2.23340	1.00270
C	3.19510	-2.51040	-0.10930
C	4.05040	-1.54620	-0.66470
C	4.06390	-0.26490	-0.09460
C	3.25280	0.06260	1.00150
C	2.42550	-0.93520	1.51240
O	3.20360	-3.75410	-0.70170
O	4.82950	0.74170	-0.63210
H	1.80260	-0.69390	2.36510
C	3.30660	1.47050	1.60410
C	2.22320	2.37410	1.00090
C	2.49920	3.18100	-0.11500
C	1.53700	4.04000	-0.66870
C	0.25890	4.06350	-0.09170
C	-0.06710	3.25820	1.00910
C	0.92810	2.42630	1.51740
O	3.74000	3.18210	-0.71290
O	-0.74600	4.83370	-0.62590
H	0.68740	1.80830	2.37370
C	-1.47240	3.32000	1.61620
C	-3.30190	-1.47410	3.13920
C	1.45120	-3.35320	3.15760
H	1.89950	-4.28050	1.30480
C	3.34360	1.44120	3.15540
H	4.26920	1.88790	1.30160
C	-1.43970	3.35490	3.16730
H	-1.88610	4.28460	1.31550
H	-4.08500	-0.77840	3.46450
H	-2.36290	-1.07030	3.53140
C	-3.55110	-2.84420	3.80000
H	0.52510	-3.85700	3.45110
H	1.39070	-2.34470	3.58100
C	2.61630	-4.11150	3.82790
H	3.84900	0.51590	3.44830
H	2.33580	1.37940	3.58040
C	4.10170	2.60730	3.82440
H	-0.51310	3.85880	3.45890
H	-1.37870	2.34620	3.59030
C	-2.60370	4.11270	3.84020
H	-4.25410	-1.88400	1.29060
C	1.85290	4.84530	-1.92140
H	-4.15560	-2.29360	-0.68950
H	2.31490	-4.17750	-0.68300
H	5.66310	0.42100	-1.07160
H	4.16530	2.29440	-0.68580
H	-0.42170	5.66260	-1.07130
H	-2.31420	4.18580	-0.66720
O	0.75480	-4.83570	-0.63520
C	-4.86580	1.87610	-1.89510
H	-5.66180	-0.41160	-1.05740
C	-1.84600	-4.85310	-1.92720
H	0.43040	-5.66650	-1.07670
C	-6.30400	2.33050	-1.69510

C	-6.66620	3.66570	-1.60290
C	-8.08600	4.01310	-1.56650
C	-9.01810	2.96670	-1.56420
O	-8.63160	1.66390	-1.58140
C	-7.29930	1.30650	-1.64220
O	-7.08670	0.09670	-1.64270
N	-5.74570	4.65700	-1.59230
C	-8.58300	5.32910	-1.55230
C	-9.94430	5.58100	-1.52800
C	-10.85190	4.51580	-1.51680
C	-10.39190	3.20950	-1.53630
C	-2.31330	-6.28830	-1.73310
C	-3.65180	-6.64080	-1.65200
C	-4.00980	-8.05820	-1.61850
C	-2.97030	-8.99790	-1.60900
O	-1.66470	-8.62100	-1.61670
C	-1.29710	-7.29120	-1.67250
O	-0.08570	-7.08790	-1.66250
C	-5.32950	-8.54570	-1.61300
C	-5.59140	-9.90510	-1.59060
C	-4.53290	-10.82050	-1.57250
C	-3.22330	-10.37000	-1.58300
N	-4.63620	-5.71330	-1.64920
C	4.86280	-1.86480	-1.91190
C	6.30130	-2.31970	-1.71380
C	9.01510	-2.95790	-1.58590
O	8.62950	-1.65490	-1.60150
C	7.29730	-1.29660	-1.66050
O	7.08580	-0.08650	-1.65840
C	6.66270	-3.65540	-1.62390
C	8.08240	-4.00380	-1.58890
N	5.74160	-4.64580	-1.61420
C	8.57870	-5.32010	-1.57630
C	9.93980	-5.57280	-1.55280
C	10.84800	-4.50820	-1.54080
C	10.38890	-3.20160	-1.55880
C	2.31890	6.28190	-1.73360
C	2.97430	8.99270	-1.62440
O	1.66900	8.61480	-1.62890
C	1.30220	7.28450	-1.67760
O	0.09100	7.08050	-1.66470
C	3.65730	6.63590	-1.65540
C	4.01440	8.05360	-1.63000
N	4.64240	5.70910	-1.64810
C	5.33370	8.54210	-1.62880
C	5.59480	9.90170	-1.61400
C	4.53570	10.81650	-1.59940
C	3.22640	10.36510	-1.60610
H	0.95750	4.87320	-2.54680
H	2.61150	4.30340	-2.48810
H	-4.89090	0.98560	-2.52760
H	-4.33250	2.64300	-2.45870
H	-0.95150	-4.88400	-2.55360
H	-2.60460	-4.31230	-2.49510
H	-6.04340	5.59000	-1.36560
H	-7.90220	6.17140	-1.57680
H	-10.30400	6.60270	-1.52030
H	-11.91780	4.70980	-1.49600
H	-11.06590	2.36230	-1.53180
H	-6.16680	-7.85900	-1.64250
H	-6.61570	-10.25740	-1.58960
H	-4.73480	-11.88490	-1.55320
H	-2.38100	-11.05000	-1.57280
H	-5.57390	-6.00380	-1.43300
H	4.88710	-0.97380	-2.54370
H	4.32880	-2.63130	-2.47550
H	6.03860	-5.57950	-1.38960
H	7.89740	-6.16210	-1.60150
H	10.29890	-6.59480	-1.54630
H	11.91390	-4.70290	-1.52060
H	11.06330	-2.35480	-1.55370
H	5.57930	6.00190	-1.43170
H	6.17150	7.85570	-1.65610
H	6.61890	10.25470	-1.61630
H	4.73700	11.88120	-1.58610
H	2.38360	11.04460	-1.59860
C	-3.45540	-2.71260	5.32700

C	-4.89810	-3.46150	3.39520
C	-2.32000	4.27850	5.34070
C	-3.98060	3.46880	3.62680
C	4.26890	2.32830	5.32560
C	3.45830	3.98370	3.60710
C	2.33600	-4.27840	5.32890
C	3.99280	-3.46770	3.61180
H	-2.75880	-3.52700	3.47200
H	2.64300	-5.11570	3.38250
H	5.10590	2.63340	3.37880
H	-2.63130	5.11730	3.39570
H	-2.48420	-2.31220	5.63350
H	-4.22940	-2.03970	5.71250
H	-3.58570	-3.68210	5.81680
H	-5.72790	-2.79060	3.64480
H	-4.94800	-3.67390	2.32470
H	-5.06430	-4.40520	3.92330
H	-2.27660	3.30400	5.83970
H	-1.36670	4.78580	5.51610
H	-3.10580	4.86330	5.82800
H	-4.24280	3.39580	2.57050
H	-4.00720	2.45620	4.04250
H	-4.75750	4.05470	4.12810
H	3.29490	2.28640	5.82560
H	4.77630	1.37560	5.50350
H	4.85410	3.11560	5.80990
H	3.38160	4.24130	2.54980
H	2.44720	4.01300	4.02670
H	4.04640	4.76240	4.10290
H	2.29390	-3.30420	5.82870
H	1.38290	-4.78540	5.50600
H	3.12270	-4.86370	5.81400
H	4.25140	-3.39110	2.55480
H	4.02120	-2.45660	4.03140
H	4.77120	-4.05530	4.10850
H	-4.77990	4.43340	-1.35290
H	-4.40760	-4.74900	-1.40870
H	4.77610	-4.42170	-1.37370
H	4.41420	4.74640	-1.40010

Crown-*out* $CHCl_3$

E -4675.5802007 H

188

C	2.39280	-2.22050	1.00380
C	3.20910	-2.49270	-0.10620
C	4.06240	-1.52360	-0.65810
C	4.06690	-0.24300	-0.08610
C	3.24710	0.08150	1.00620
C	2.42440	-0.92190	1.51400
O	3.22630	-3.73490	-0.69960
O	4.83130	0.76800	-0.61700
H	1.79230	-0.68620	2.36070
C	3.28240	1.49530	1.59380
C	2.19650	2.39150	0.98880
C	2.46800	3.20370	-0.12420
C	1.50000	4.05930	-0.67440
C	0.22240	4.07240	-0.09530
C	-0.09880	3.26230	1.00470
C	0.90330	2.43800	1.51230
O	3.70630	3.21020	-0.72580
H	0.67160	1.81870	2.37050
C	-1.50580	3.30650	1.60980
C	-2.39820	2.21540	1.00300
C	-3.21150	2.48740	-0.10990
C	-4.06260	1.51860	-0.66480
C	-4.06940	0.23760	-0.09300
C	-3.25390	-0.08630	1.00180
C	-2.43210	0.91660	1.51290
O	-3.22950	3.73040	-0.70170
O	-4.83550	-0.77170	-0.62450
H	-1.80750	0.67890	2.36530
C	-3.29700	-1.49560	1.60290
C	-2.20740	-2.38990	0.99630

C	-2.47870	-3.19850	-0.12030
C	-1.51160	-4.05250	-0.67420
C	-0.23290	-4.06740	-0.09740
C	0.08930	-3.25870	1.00270
C	-0.91150	-2.43340	1.51240
O	-3.71920	-3.20970	-0.71720
O	0.77590	-4.83490	-0.62810
H	-0.67450	-1.81420	2.36880
C	1.49610	-3.30890	1.60800
C	3.29150	1.49030	3.13980
C	-1.47800	3.33710	3.16130
H	-1.93160	4.26590	1.30990
C	-3.33010	-1.47070	3.15450
H	-4.25670	-1.91970	1.30150
C	1.46780	-3.34170	3.15940
H	1.91780	-4.26970	1.30690
H	4.07580	0.79450	3.46170
H	2.35300	1.08350	3.52910
C	3.54200	2.85670	3.80880
H	-0.55280	3.83900	3.46030
H	-1.42040	2.32710	3.58080
C	-2.64400	4.09560	3.82970
H	-3.83320	-0.54620	3.45360
H	-2.32090	-1.41280	3.57600
C	-4.08940	-2.63770	3.82010
H	0.54220	-3.84380	3.45680
H	1.41040	-2.33230	3.58030
C	2.63300	-4.10150	3.82770
H	4.24160	1.91550	1.29310
C	-1.82440	-4.86490	-1.92370
H	4.14150	2.32890	-0.68580
H	-2.34740	4.16520	-0.67430
H	-5.65630	-0.45260	-1.09070
H	-4.15410	-2.32770	-0.68380
H	0.45550	-5.65220	-1.09960
H	2.34250	-4.16650	-0.67490
O	-0.78690	4.84120	-0.62320
C	4.88230	-1.83650	-1.90240
H	5.65360	0.45020	-1.08140
C	1.81080	4.87440	-1.92240
H	-0.46630	5.66160	-1.08930
C	6.32890	-2.26400	-1.69980
C	6.71500	-3.59580	-1.59420
C	8.14110	-3.91800	-1.55580
C	9.05500	-2.85660	-1.57910
O	8.64240	-1.55860	-1.61770
C	7.30630	-1.22910	-1.67380
O	7.07570	-0.01660	-1.69690
N	5.81370	-4.59720	-1.56650
C	8.66070	-5.22540	-1.51150
C	10.02670	-5.45080	-1.48920
C	10.91590	-4.36940	-1.50900
C	10.43310	-3.07160	-1.55440
C	2.25020	6.31830	-1.72490
C	3.58540	6.69550	-1.63090
C	3.91730	8.11940	-1.59600
C	2.86190	9.04050	-1.61120
O	1.56090	8.63660	-1.63890
C	1.22210	7.30270	-1.69160
O	0.00790	7.08020	-1.70540
C	5.22850	8.63030	-1.56240
C	5.46320	9.99480	-1.54270
C	4.38770	10.89120	-1.55450
C	3.08640	10.41710	-1.58910
N	4.58100	5.78760	-1.61140
C	-4.87960	1.83200	-1.91100
C	-6.32660	2.26090	-1.71310
C	-9.05290	2.85580	-1.60360
O	-8.64110	1.55750	-1.63810
C	-7.30510	1.22700	-1.68920
O	-7.07550	0.01420	-1.70980
C	-6.71220	3.59320	-1.61140
C	-8.13810	3.91660	-1.57900
N	-5.81020	4.59400	-1.58240
C	-8.65690	5.22450	-1.53940
C	-10.02280	5.45100	-1.52270
C	-10.91280	4.37030	-1.54360

C	-10.43080	3.07200	-1.58440
C	-2.25860	-6.31100	-1.73030
C	-2.86280	-9.03540	-1.62770
O	-1.56310	-8.62800	-1.66040
C	-1.22800	-7.29300	-1.70790
O	-0.01440	-7.06740	-1.72670
C	-3.59230	-6.69220	-1.63080
C	-3.92050	-8.11710	-1.60190
N	-4.59000	-5.78690	-1.60070
C	-5.23020	-8.63150	-1.56430
C	-5.46130	-9.99670	-1.55070
C	-4.38360	-10.89030	-1.57280
C	-3.08370	-10.41270	-1.61170
H	-0.93620	-4.87300	-2.55990
H	-2.59910	-4.33850	-2.48290
H	4.89000	-0.95020	-2.54120
H	4.36210	-2.61500	-2.46210
H	0.92040	4.88750	-2.55550
H	2.58160	4.34750	-2.48640
H	6.11900	-5.53710	-1.38020
H	7.99670	-6.08080	-1.50420
H	10.40490	-6.46510	-1.45740
H	11.98500	-4.54400	-1.49020
H	11.09430	-2.21460	-1.57150
H	6.07940	7.96060	-1.56120
H	6.48020	10.36620	-1.51930
H	4.56960	11.95910	-1.53790
H	2.23360	11.08400	-1.59970
H	5.52490	6.08650	-1.43550
H	-4.88640	0.94550	-2.54960
H	-4.35730	2.60990	-2.46960
H	-6.11560	5.53420	-1.39800
H	-7.99220	6.07940	-1.53160
H	-10.40040	6.46560	-1.49460
H	-11.98180	4.54580	-1.52920
H	-11.09260	2.21540	-1.60230
H	-5.53160	-6.08950	-1.41850
H	-6.08280	-7.96400	-1.55540
H	-6.47730	-10.37090	-1.52390
H	-4.56280	-11.95870	-1.56080
H	-2.22940	-11.07740	-1.63040
C	3.44120	2.71660	5.33480
C	4.89250	3.47160	3.41290
C	2.36180	-4.25150	5.33230
C	4.01240	-3.46950	3.59580
C	-4.24320	-2.36840	5.32470
C	-3.45520	-4.01610	3.58820
C	-2.37390	4.24490	5.33460
C	-4.02280	3.46290	3.59640
H	2.75240	3.54400	3.48320
H	-2.66120	5.10440	3.39460
H	-5.09700	-2.65550	3.38220
H	2.64970	-5.11000	3.39210
H	2.46870	2.31510	5.63550
H	4.21390	2.04050	5.71760
H	3.57150	3.68360	5.82940
H	5.71800	2.79480	3.66090
H	4.94810	3.69160	2.34410
H	5.05990	4.41060	3.94870
H	2.33020	-3.27200	5.82250
H	1.40590	-4.74980	5.51930
H	3.14730	-4.83860	5.81710
H	4.26990	-3.41870	2.53660
H	4.04740	-2.44980	3.99360
H	4.78800	-4.05210	4.10280
H	-3.26480	-2.33510	5.81700
H	-4.74390	-1.41380	5.51160
H	-4.82950	-3.15570	5.80750
H	-3.39850	-4.27120	2.52870
H	-2.43760	-4.05100	3.99140
H	-4.03950	-4.79330	4.09060
H	-2.34170	3.26510	5.82420
H	-1.41850	4.74370	5.52250
H	-3.16020	4.83100	5.81920
H	-4.27850	3.40990	2.53680
H	-4.05800	2.44390	3.99630
H	-4.79950	4.04590	4.10110

H	4.83850	-4.39480	-1.35420
H	4.37390	4.81400	-1.39680
H	-4.83640	4.39070	-1.36450
H	-4.38390	-4.81410	-1.38140

Crown-*out* DMSO

E = -4675.59302281

188

**C**	**-2.77600**	**1.72370**	**0.99040**
**C**	**-3.63410**	**1.83930**	**-0.11610**
**C**	**-4.29090**	**0.72710**	**-0.66780**
**C**	**-4.04760**	**-0.53320**	**-0.10210**
**C**	**-3.17560**	**-0.69980**	**0.98600**
**C**	**-2.55860**	**0.44110**	**1.49680**
**O**	**-3.89200**	**3.05630**	**-0.70410**
**O**	**-4.60750**	**-1.66940**	**-0.63450**
**H**	**-1.89450**	**0.32850**	**2.34430**
**C**	**-2.93630**	**-2.09730**	**1.56510**
**C**	**-1.69830**	**-2.76240**	**0.95370**
**C**	**-1.80860**	**-3.60540**	**-0.16470**
**C**	**-0.69480**	**-4.26180**	**-0.71370**
**C**	**0.56130**	**-4.03500**	**-0.13090**
**C**	**0.72130**	**-3.18240**	**0.97270**
**C**	**-0.42070**	**-2.56630**	**1.48180**
**O**	**-3.01990**	**-3.84390**	**-0.77250**
**H**	**-0.31320**	**-1.92280**	**2.34650**
**C**	**2.10970**	**-2.96000**	**1.58140**
**C**	**2.77800**	**-1.71760**	**0.97710**
**C**	**3.63320**	**-1.82780**	**-0.13270**
**C**	**4.28990**	**-0.71370**	**-0.68000**
**C**	**4.04930**	**0.54430**	**-0.10710**
**C**	**3.18060**	**0.70560**	**0.98330**
**C**	**2.56330**	**-0.43720**	**1.49040**
**O**	**3.89030**	**-3.04200**	**-0.72720**
**O**	**4.61270**	**1.68180**	**-0.63290**
**H**	**1.90590**	**-0.32540**	**2.34400**
**C**	**2.94970**	**2.09630**	**1.58540**
**C**	**1.70790**	**2.76210**	**0.97690**
**C**	**1.81740**	**3.60370**	**-0.14360**
**C**	**0.70390**	**4.25860**	**-0.69460**
**C**	**-0.55280**	**4.03280**	**-0.11270**
**C**	**-0.71290**	**3.17980**	**0.99000**
**C**	**0.42870**	**2.56190**	**1.49900**
**O**	**3.02940**	**3.84950**	**-0.74680**
**O**	**-1.68970**	**4.59610**	**-0.64000**
**H**	**0.31740**	**1.91700**	**2.36190**
**C**	**-2.10230**	**2.96090**	**1.59840**
**C**	**-2.94390**	**-2.10580**	**3.11150**
**C**	**2.08630**	**-2.99650**	**3.13310**
**H**	**2.71130**	**-3.81990**	**1.28060**
**C**	**2.98480**	**2.07820**	**3.13730**
**H**	**3.80900**	**2.69900**	**1.28470**
**C**	**-2.07900**	**2.99160**	**3.15020**
**H**	**-2.69980**	**3.82470**	**1.30080**
**H**	**-3.85020**	**-1.58180**	**3.43880**
**H**	**-2.10400**	**-1.52510**	**3.50500**
**C**	**-2.92010**	**-3.50010**	**3.76910**
**H**	**1.26980**	**-3.66090**	**3.43160**
**H**	**1.84260**	**-2.01540**	**3.55340**
**C**	**3.37210**	**-3.52770**	**3.80120**
**H**	**3.65220**	**1.26530**	**3.43850**
**H**	**2.00460**	**1.83140**	**3.55800**
**C**	**3.51080**	**3.36790**	**3.80190**
**H**	**-1.26190**	**3.65400**	**3.45120**
**H**	**-1.83640**	**2.00860**	**3.56680**
**C**	**-3.36430**	**3.52110**	**3.82050**
**H**	**-3.79610**	**-2.69320**	**1.26080**
**C**	**0.85460**	**5.11910**	**-1.94220**
**H**	**-3.62440**	**-3.07060**	**-0.71290**
**H**	**3.11110**	**-3.64140**	**-0.69590**
**H**	**5.47880**	**1.52610**	**-1.10260**
**H**	**3.63490**	**3.07600**	**-0.69630**

H	-1.53010	5.45480	-1.12190
H	-3.11010	3.65240	-0.67420
O	1.69860	-4.59810	-0.65740
C	-5.17200	0.88470	-1.89980
H	-5.47450	-1.51290	-1.10190
C	-0.84520	-5.12670	-1.95800
H	1.53980	-5.45960	-1.13450
C	-6.67190	1.01300	-1.67400
C	-7.31340	2.24160	-1.53880
C	-8.77420	2.27310	-1.47670
C	-9.45970	1.05190	-1.51210
O	-8.79660	-0.13810	-1.58370
C	-7.42430	-0.19280	-1.66050
O	-6.95810	-1.33760	-1.71250
N	-6.62890	3.39930	-1.50040
C	-9.54220	3.45030	-1.39670
C	-10.92530	3.39800	-1.35360
C	-11.58240	2.16180	-1.38730
C	-10.85220	0.98690	-1.46660
C	-0.99000	-6.62820	-1.75190
C	-2.22560	-7.26080	-1.64110
C	-2.26980	-8.72200	-1.59670
C	-1.05400	-9.41740	-1.62360
O	0.14220	-8.76350	-1.67130
C	0.20920	-7.39100	-1.73120
O	1.35850	-6.93410	-1.76290
C	-3.45420	-9.48110	-1.54150
C	-3.41400	-10.86500	-1.51380
C	-2.18290	-11.53200	-1.53850
C	-1.00110	-10.81070	-1.59350
N	-3.37820	-6.56740	-1.61050
C	5.17100	-0.86590	-1.91270
C	6.67100	-0.99750	-1.68830
C	9.45920	-1.04120	-1.53000
O	8.79770	0.15000	-1.59490
C	7.42530	0.20710	-1.66980
O	6.96090	1.35290	-1.71590
C	7.31110	-2.22770	-1.56060
C	8.77200	-2.26150	-1.50000
N	6.62530	-3.38470	-1.52770
C	9.53840	-3.44020	-1.42670
C	10.92160	-3.39010	-1.38460
C	11.58040	-2.15470	-1.41280
C	10.85180	-0.97840	-1.48550
C	0.99630	6.62220	-1.74560
C	1.05600	9.41280	-1.64210
O	-0.13920	8.75660	-1.68310
C	-0.20400	7.38350	-1.73190
O	-1.35270	6.92470	-1.76040
C	2.23090	7.25790	-1.64010
C	2.27280	8.71960	-1.60950
N	3.38440	6.56650	-1.60280
C	3.45610	9.48080	-1.56270
C	3.41390	10.86480	-1.54800
C	2.18180	11.52970	-1.57780
C	1.00090	10.80630	-1.62500
H	-0.01610	4.95420	-2.58130
H	1.71870	4.75640	-2.50020
H	-5.01190	0.02230	-2.55130
H	-4.82530	1.75750	-2.45430
H	0.02670	-4.96690	-2.59680
H	-1.70770	-4.76520	-2.51920
H	-7.11010	4.26230	-1.31230
H	-9.06270	4.42070	-1.37530
H	-11.49690	4.31590	-1.29410
H	-12.66430	2.11980	-1.35230
H	-11.33140	0.01650	-1.49450
H	-4.42070	-8.99360	-1.52740
H	-4.33720	-11.42940	-1.47320
H	-2.15030	-12.61450	-1.51550
H	-0.03430	-11.29770	-1.61400
H	-4.24790	-7.04340	-1.44110
H	5.01190	0.00020	-2.55950
H	4.82310	-1.73530	-2.47190
H	7.10560	-4.24930	-1.34460
H	9.05750	-4.41000	-1.40960
H	11.49190	-4.30910	-1.33020

H	12.66240	-2.11440	-1.37860
H	11.33230	-0.00850	-1.50900
H	4.25280	7.04550	-1.43480
H	4.42330	8.99470	-1.54550
H	4.33630	11.43100	-1.51380
H	2.14760	12.61240	-1.56500
H	0.03350	11.29160	-1.64930
C	-2.85170	-3.35410	5.29640
C	-4.12120	-4.36790	3.36530
C	-3.12920	3.69530	5.32860
C	-4.60720	2.65550	3.57120
C	3.69150	3.13840	5.31000
C	2.63930	4.60640	3.55160
C	3.13750	-3.70860	5.30850
C	4.61420	-2.66020	3.55510
H	-2.00890	-4.01450	3.44190
H	3.56870	-4.52090	3.37450
H	4.50360	3.56670	3.37530
H	-3.56020	4.51620	3.39810
H	-1.97760	-2.77170	5.60270
H	-3.74320	-2.84540	5.68010
H	-2.79100	-4.33160	5.78360
H	-5.06460	-3.87250	3.62130
H	-4.13450	-4.58310	2.29410
H	-4.09630	-5.32720	3.89050
H	-2.92550	2.72980	5.80480
H	-2.27720	4.35230	5.52710
H	-4.00800	4.12720	5.81630
H	-4.85030	2.58050	2.50990
H	-4.45880	1.63880	3.95020
H	-5.47540	3.08010	4.08480
H	2.72880	2.93140	5.79030
H	4.35340	2.29020	5.50880
H	4.12080	4.02080	5.79350
H	2.55720	4.84420	2.48960
H	1.62540	4.45590	3.93730
H	3.06360	5.47860	4.05870
H	2.93250	-2.74540	5.78880
H	2.28650	-4.36760	5.50440
H	4.01700	-4.14120	5.79430
H	4.85590	-2.57900	2.49380
H	4.46570	-1.64580	3.94010
H	5.48320	-3.08710	4.06520
H	-5.62790	3.39570	-1.31810
H	-3.36870	-5.56880	-1.41560
H	5.62460	-3.38050	-1.34320
H	3.37530	5.57020	-1.39550
